# A General Approach for Haplotype Phasing across the Full Spectrum of Relatedness

**DOI:** 10.1371/journal.pgen.1004234

**Published:** 2014-04-17

**Authors:** Jared O'Connell, Deepti Gurdasani, Olivier Delaneau, Nicola Pirastu, Sheila Ulivi, Massimiliano Cocca, Michela Traglia, Jie Huang, Jennifer E. Huffman, Igor Rudan, Ruth McQuillan, Ross M. Fraser, Harry Campbell, Ozren Polasek, Gershim Asiki, Kenneth Ekoru, Caroline Hayward, Alan F. Wright, Veronique Vitart, Pau Navarro, Jean-Francois Zagury, James F. Wilson, Daniela Toniolo, Paolo Gasparini, Nicole Soranzo, Manjinder S. Sandhu, Jonathan Marchini

**Affiliations:** 1 Wellcome Trust Centre for Human Genetics, University of Oxford, Oxford, United Kingdom; 2 Department of Statistics, University of Oxford, Oxford, United Kingdom; 3 Wellcome Trust Sanger Institute, Hinxton, United Kingdom; 4 Department of Public Health and Primary Care, University of Cambridge, Cambridge, United Kingdom; 5 Institute for Maternal and Child Health - IRCCS Burlo Garofolo, University of Trieste, Trieste, Italy; 6 Institute for Maternal and Child Health - IRCCS Burlo Garofolo, Trieste, Italy; 7 Division of Genetics and Cell Biology, San Raffaele Scientific Institute, Milano, Italy; 8 MRC Human Genetics Unit, MRC Institute of Genetics and Molecular Medicine, University of Edinburgh, Edinburgh, United Kingdom; 9 Centre for Population Health Sciences, University of Edinburgh, Edinburgh, United Kingdom; 10 Faculty of Medicine, University of Split, Split, Croatia; 11 Medical Research Council/Uganda Virus Research Institute (MRC/UVRI), Uganda Research Unit on AIDS, Entebbe, Uganda; 12 Laboratoire Génomique, Bioinformatique, et Applications (EA4627), Conservatoire National des Arts et Métiers, Paris, France; Georgia Institute of Technology, United States of America

## Abstract

Many existing cohorts contain a range of relatedness between genotyped individuals, either by design or by chance. Haplotype estimation in such cohorts is a central step in many downstream analyses. Using genotypes from six cohorts from isolated populations and two cohorts from non-isolated populations, we have investigated the performance of different phasing methods designed for nominally ‘unrelated’ individuals. We find that SHAPEIT2 produces much lower switch error rates in all cohorts compared to other methods, including those designed specifically for isolated populations. In particular, when large amounts of IBD sharing is present, SHAPEIT2 infers close to perfect haplotypes. Based on these results we have developed a general strategy for phasing cohorts with any level of implicit or explicit relatedness between individuals. First SHAPEIT2 is run ignoring *all* explicit family information. We then apply a novel HMM method (duoHMM) to combine the SHAPEIT2 haplotypes with any family information to infer the inheritance pattern of each meiosis at all sites across each chromosome. This allows the correction of switch errors, detection of recombination events and genotyping errors. We show that the method detects numbers of recombination events that align very well with expectations based on genetic maps, and that it infers far fewer spurious recombination events than Merlin. The method can also detect genotyping errors and infer recombination events in otherwise uninformative families, such as trios and duos. The detected recombination events can be used in association scans for recombination phenotypes. The method provides a simple and unified approach to haplotype estimation, that will be of interest to researchers in the fields of human, animal and plant genetics.

## Author Summary

Every individual carries two copies of each chromosome (haplotypes), one from each of their parents, that consist of a long sequence of alleles. Modern genotyping technologies do not measure haplotypes directly, but the combined sum (or genotype) of alleles at each site. Statistical methods are needed to infer (or phase) the haplotypes from the observed genotypes. Haplotype estimation is a key first step of many disease and population genetic studies. Much recent work in this area has focused on phasing in cohorts of nominally unrelated individuals. So called ‘long range phasing’ is a relatively recent concept for phasing individuals with intermediate levels of relatedness, such as cohorts taken from population isolates. Methods also exist for phasing genotypes for individuals within explicit pedigrees. Whilst high quality phasing techniques are available for each of these demographic scenarios, to date, no single method is applicable to all three. In this paper, we present a general approach for phasing cohorts that contain any level of relatedness between the study individuals. We demonstrate high levels of accuracy in all demographic scenarios, as well as the ability to detect (Mendelian consistent) genotyping error and recombination events in duos and trios, the first method with such a capability.

## Introduction

The estimation of haplotypes from SNP genotypes, commonly referred to as ‘phasing’, is a well studied problem in the literature. Existing approaches can be categorised according to the type of cohort each method is designed to phase, and the level of relatedness between the individuals in that cohort. Much of the recent literature is devoted to phasing nominally *unrelated* (or distantly related) individuals. Currently, the most accurate methods use hidden Markov models (HMMs) to model local haplotype sharing between individuals [1], [2], and take advantage of linkage disequilibrium (LD). Some of these methods can also handle mother-father-child trios and parent-child duos [2]–[6], for more complex pedigrees there are several general pedigree analysis software packages [7]–[10].

However such methods face several limitations; Lander-Green algorithm based approaches have computational and space complexity that scale exponentially with sample size; they can be sensitive to genotyping error and they can only phase sites where at least one member of the pedigree is not heterozygous. The last point is particularly crucial, as it means the haplotypes will not be ‘complete’ and cannot be easily used in pre-phasing and imputation which is now a standard part GWAS pipelines [11]. If founders in these pedigrees have been sequenced with the aim of imputing sequenced variants from founders into descendants who have been assayed on microarrays, then a pedigree phasing method that overcomes these issues will be especially useful.

The task of phasing in isolated populations is some what of a special case, as individuals from such populations exhibit much higher levels of relatedness, and will tend to share much longer stretches of sequence identically by descent (IBD) than a pair of unrelated individuals from a non-isolated population. Kong at al.(2008) [12] proposed a method in which surrogate parents are identified for each individual in a given region of the genome. These surrogate parents allow the haplotypes to be determined with high accuracy using Mendelian inheritance rules, effectively as if the true parents had been observed and the family could be phased as a trio. More recently, a model based version of this approach called Systematic Long Range Phasing (SLRP) has been proposed [13]. Both of these papers demonstrated accurate haplotype estimates within IBD regions, but suffer from the problem that phase can only be inferred for genomic regions where IBD sharing is detected. Even in IBD regions, if a site is heterozygous in all individuals, the phase at that particular locus cannot be inferred.

So far in the literature there has been very little investigation of the performance of methods for phasing in isolated populations. In addition, many GWAS cohorts consist of a range of relatedness between the study individuals. Some cohorts contain mixtures of pedigrees, weakly or cryptically related individuals and more distantly related individuals. Methods for carrying out association studies using related individuals have recently been re-discovered in the literature as a powerful approach, with the additional benefit of implicitly avoiding confounding due to population structure [14]–[16]. In addition, explicit detection of tracts of IBD between pairs of individuals is becoming more widely used for detection of disease genes [17]–[20] and for population genetic analyses [21], [22]. More generally, isolated populations offer promise for interrogating common complex diseases [23]. For many such cohorts phasing will be a first step in performing imputation from a reference panel [11] or as part of an IBD detection analysis, so it is interesting to consider the performance of alternative phasing methods.

We recently compared several methods all designed to phase nominally unrelated samples (SHAPEIT2 [2], SHAPEIT1 [5], Beagle [4], HAPI-UR [6], Impute2 [24], MaCH [25], fastPHASE [26]) and found that SHAPEIT2 was the most accurate method in this setting. In this paper we examine the performance of these methods at increasing levels of relatedness between individuals. To do this we used cohorts from six different isolated populations (and two additional cohorts from non-isolated populations). Each of these cohorts contain some extended pedigrees allowing us to assess performance on both nominally unrelated individuals and on explicitly related samples.

For cohorts with explicitly related samples we introduce a new hidden Markov model (which we call duoHMM) that can estimate the inheritance pattern between between the haplotypes of each parent-child duo. This method can be used to visualise the inheritance status across a chromosome, correct phasing errors that are inconsistent with pedigree information, and detect genotyping errors. We show that after applying this adjustment, SHAPEIT2's haplotypes are accurate enough that we can detect explicit recombination events between parent-child pairs. Applying this method to the SHAPEIT2 inferred haplotypes provides the most accurate performance in the extended pedigree setting. Using our method we are able to demonstrate that the recombination events that we infer from otherwise uninformative duos and trios can add power to association scans for recombination phenotypes. Specifically, at the established *PRDM9* locus we are able to show that including these extra recombination events increases the signal of association for a hot spot usage phenotype. Overall, the combination of SHAPEIT2 and duoHMM provides a very general method for accurate phasing of cohorts with any levels of implicit or explicit relatedness between individuals.

SHAPEIT2 and duoHMM are available from the website: http://www.stats.ox.ac.uk/marchini/software/gwas/gwas.html

## Materials and Methods

### Real datasets

To provide a comprehensive assessment of the accuracy of methods we analysed eight different cohorts that vary in the extent of the relatedness between individuals. The cohorts are summarised in Table S1, six of these are considered to be from isolated populations. The Orkney Complex Disease Study (ORCADES) is an ongoing study in the isolated Scottish archipelago of Orkney [27]. The CROATIA-VIS (Vis) and CROATIA-KORCULA (Korcula) studies contain individuals recruited from the Dalmation islands of Vis and Korcula [27], [28]. The INGI-Val Borbera population is a collection of 1,664 genotyped individuals collected in the Val Borbera region, a geographically isolated valley located within the Appennine Mountains in Northwest Italy. The valley is inhabited by about 3,000 descendants from the original population, living in seven villages along the valley and in the mountains [29]. The INGI-FVG Cohort is a collection of six different isolated villages in the Friuli Venezia Giulia region of northern Italy [30]. The INGI-CARL cohort contains individuals from Carlantino, a small isolated village in the province of Foggia in southern Italy [30]. The CROATIA-Split (Split) cohort contains individuals from the Croatian city of Split [31]. Finally, a large sample from the Ugandan General Population Cohort (GPC) [32], covering residents of 25 villages in south-Western Uganda were analysed. These final two cohorts are not considered to be isolated and hence are useful as control samples of unrelated individuals. Each of these cohorts contain pedigrees of varying sizes (see Table S1) which can be used to evaluate phasing accuracy. The GPC cohort was genotyped using the Illumina Human OMNI 2.5S chip. All the other cohorts were genotyped using either the Illumina HumanHap300 or HumanCNV370 chips.

In addition to quality control (QC) performed on each cohort by their respective research groups, we applied stringent filters to remove genotypes inconsistent with pedigree structure. Firstly, we ran Pedstats [33] to detect any genotypes that violated Mendelian constraints, and these loci were marked as missing for all individuals in a pedigree where violations were found. Loci that produced Mendel violations for 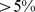 of samples were filtered for *all* individuals in a cohort. Secondly, Merlin's error detection algorithm was used on all pedigrees, and genotypes which were unlikely were also flagged as missing. This final set of genotypes were used as input in all subsequent analyses.

All software was run as per instructions in their respective manuals. The computation times for each method for the largest experiment conducted on European cohorts are summarised in Figure S1, a more comprehensive study of running times was conducted in the original SHAPEIT2 paper [2].

#### Creating validation haplotypes

We phased the pedigrees in each cohort using Merlin (version 1.1.2), which produces the most likely haplotypes given the pedigree structure using the Lander-Green algorithm. The phasing occurs one pedigree at a time and no information is shared between pedigrees. These haplotypes should be highly accurate and hence suitable for validation purposes. The accuracy of the haplotypes will increase with pedigree size, so in some of our experiments we only use those haplotypes from larger pedigrees.

Merlin can only phase loci where at least one pedigree member is homozygous. We found that 50.16% of heterozygote sites could be phased for duos, 77.79% for trios and 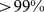 sites for pedigrees of size 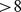 (Figure S2). Running times for Merlin increase exponentially as pedigree size increases (see Figure S3) but in general were not excessive on these data sets (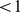 hour per cohort).

#### Haplotype accuracy in founder individuals

We merged the pedigree founders with individuals who were not in any explicit pedigree for each cohort, that is, all non-founders from pedigrees were excluded. This gave us a sample of (nominally) unrelated individuals that we phased using each of the methods. We applied SLRP (version 09f0f52), SHAPEIT2 (r613), Beagle (version 3.3.2) and HAPI-UR (version 1.01) to these founder data sets. We then calculated the switch error (SE) [34] of the haplotype estimates for the pedigree founders, treating the Merlin haplotypes as the truth. This evaluation pipeline is visualised in Figure S4.

SLRP does not produce whole chromosome haplotypes. It only phases regions of the genome where IBD sharing is detected, and can only resolve the phase of heterozygous sites when at least one of the individuals sharing IBD is homozygous at those loci. This complicates the calculation of SE between methods. We treated each of the IBD segments inferred by SLRP separately and evaluated SE within these regions. We refer to this metric as the “within IBD SE”, using it to evaluate SLRP's performance against methods that phase every site. We also calculated the SE of the SHAPEIT2, Beagle and HAPI-UR haplotypes across the whole of chromosome 10. We also report the yield of SLRP, defined as percentage of genotypes that are phased.

#### Haplotype accuracy in explicitly related individuals

Individuals in pedigrees obviously share large amounts of their genome IBD. Algorithms that have the ability to exploit IBD sharing in distantly related individuals may also work well on explicitly related individuals. Hence, we also evaluated the accuracy of SHAPEIT2, SLRP, HAPI-UR and Beagle applied to the full cohorts described here, with the full extended pedigrees included. We ran each of the methods using *no* information regarding relatedness of samples. We calculated SE for each method on the haplotypes of any individual in a pedigree larger than a mother-father-child pedigree, using the Merlin haplotypes as truth.

SHAPEIT2, Beagle and HAPI-UR all provide functionality to phase parent-child duos and mother-father-child trios, by constraining the possible haplotypes to those consistent with the *transmitted* and *untransmitted* haplotypes of each parent (the child having each of the parents' transmitted haplotypes). This approach will produce very accurate haplotypes although will return the recombined haplotypes for each parent, rather than the true parental haplotypes. Since only several recombinations occur per chromosome, this is not introducing a substantial amount of error in the context of pre-phasing/imputation but is obviously problematic for researchers wishing to study recombination.

Larger pedigrees could be divided into subsets of duos and trios but often there will exist no subdivision that allows all samples to exploit a parental relationship. For example, a family with two parents and two siblings may be divided into two duos, but partitioning a nuclear family with three children means at least one child will be phased without using parental information. There is no obvious optimum way to partition pedigrees of arbitrary size and structure. We investigated a simple method where we enumerate every possible partitioning of a pedigree into duos/trios and choose the partition that minimises the number of individuals that are not included in a duo/trio (many partitions often share the same minimum in which case one is picked at random). We applied this partitioning to the datasets and then ran Beagle (since it was the next most competitive method) taking the implied duo and trio information into account. We refer to this as the Beagle duo/trio method. Beagle was found to use substantially more memory in this setting (over 150 GB for the GPC cohort) which may be problematic for some researchers. This issue is noted in the Beagle manual and relates to missing data in parent-offspring duos and trios.

On duos and trios this method will agree perfectly with Merlin at sites that Merlin can phase. This introduces a possible confounding effect when using the Merlin haplotypes as the truth, as any errors in the Merlin haplotypes will not be detectable when compared to the Beagle duo/trio method. We show below using simulated data that Merlin is quite sensitive to genotyping error and that this does result in elevated switch errors. For this reason we only consider pedigrees that are more complex than a parent-child duo or father-mother-child trio when comparing methods. Larger pedigrees also give Merlin better ability to remove genotyping errors yielding more accurate validation haplotypes.

### Using pedigree information to improve phase, infer recombination events and detect genotyping error

The results below show that SHAPEIT2 can implicitly leverage IBD sharing and hence phase a pedigree accurately without any relationships specified. Additional use of explicit relationships is likely to lead to even greater improvements. The Lander-Green algorithm is traditionally the method of choice for phasing pedigrees but has several limitations described previously. We developed a simple HMM applicable to the SHAPEIT2 haplotypes that corrects phasing errors that are inconsistent with pedigree information. The method focuses separately on each parent-child duo and this circumvents several issues with the Lander-Green algorithm, namely;

- *complexity*: our HMM has a constant number of hidden states (4) and possible transitions between states (16) per meiosis, so our method scales as 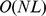 where 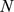 is the number of non-founders (the method runs on each parent separately) and 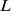 is the number of markers. This compares well to the 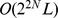 scaling for a naïve Lander-Green implementation.
- *heterozygous markers*: markers that are heterozygous throughout a pedigree will be phased via leveraging population haplotypes
- *sensitivity to genotyping error*: the low computational complexity of the model allows us to accommodate genotype uncertainty

We describe the model and several useful applications of it below. We refer to this framework as the duoHMM in later sections of the paper.

#### Duo HMM

Let 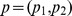 and 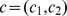 denote a pair of observed (ordered) parental and child haplotypes respectively. Here 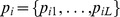 denotes the 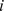
th parental haplotype at the 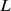 sites across a chromosome. The same notation is used for the 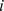
th child haplotype 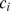. There are 4 possible patterns of gene flow between the parent and child. The true pattern of gene flow will remain constant over long stretches of a chromosome due to the low rate of recombination in any given meiosis. We use 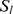 to denote the pattern of gene flow at the 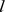
th locus, where 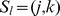 means that the parents 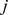
th haplotype and the child's 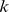
th haplotype are identical by decent (IBD). Here 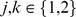, so there are just 4 possible inheritance patterns, which we denote 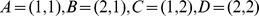. The true inheritance states 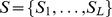 are unobserved across each chromosome and we wish to infer them from our imperfect observations of the parental and child haplotypes 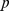 and 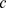. The intuition behind our approach is that true recombination events and SEs will cause changes to the pattern of gene flow as we move along a chromosome. Since we expect just a few true recombination events the SEs will tend to dominate. Thus we can think of the observed pattern of gene flow as the superposition of two point processes: one with a low rate dictated by true recombinations, and a second process with a rate relating to SEs. Our aim is to deconvolve these two processes to detect the true recombination events and correct SEs.

To carry out this inference we have developed an HMM that allows for SEs in the parental and child haplotypes. We use 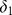 and 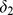 to denote the probability of a SE on the parental or child haplotypes between two adjacent markers respectively. We also use 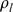 to denote the probability of a recombination occurring between markers 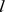 and 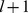. Specifically we use 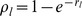 where 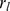 is the genetic distance between markers 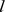 and 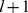. We use the genetic distances from the HapMap LD based map [35] (which are inherently sex averaged) and scale them to the sex-specific genetic lengths from the deCODE 2002 map according to the sex of the parent.

**Figure 1:**
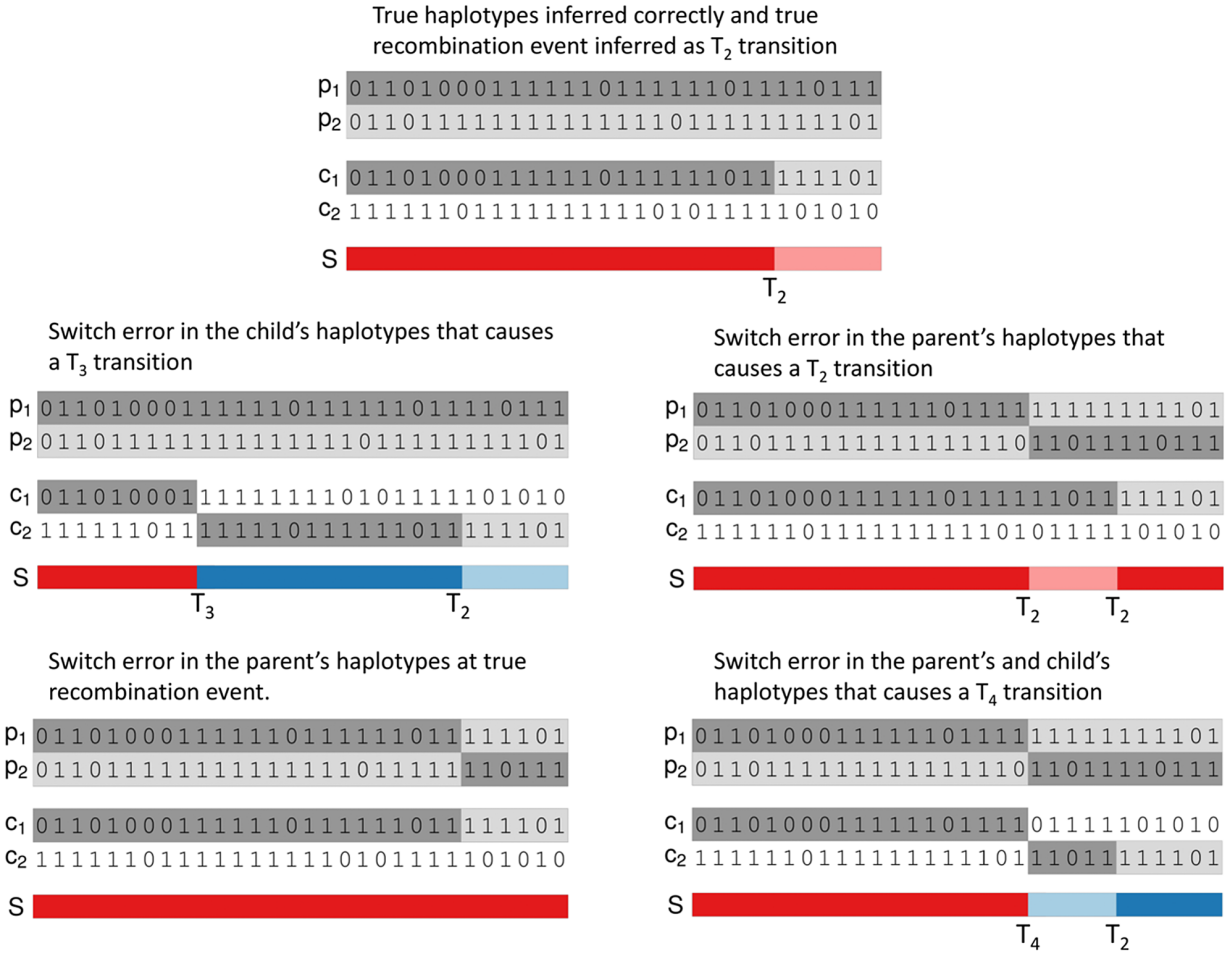
Examples of inferred haplotypes with true recombination events and SEs. In each examples 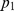, 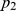, 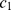 and 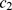 denotes the two parental and child haplotypes and 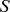 denotes the pattern of gene flow. Top: Correctly inferred haplotypes in a region of a true recombination event that causes a 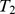 transition in the duo HMM. The other 4 examples in the figure add SEs to these true parental and child haplotypes. Middle left: addition of a SE in the child's haplotypes that causes a 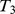 transition. Middle right: addition of a SE in the parent's haplotypes that causes a 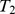 transition. Bottom left: addition of a SE in the parent's haplotypes at the site of the recombination event that causes the 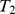 transition to be missed. Bottom right: addition of a SE in both the child's and parent's haplotypes at the same position that causes a 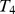 transition.

The initial states of the Markov model are given by 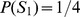. The transition rates on the IBD states 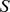 are then given by
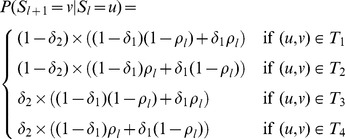
The sets 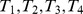 denote the different types of transition that can occur. The set 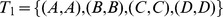 contains the transitions where no change in gene flow occurs. The set 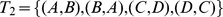 are the transitions with a change only to which of the parent's haplotypes are IBD. The set 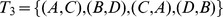 are the transitions with change only to which of the child's haplotypes are IBD. The set 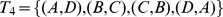 are the transitions with a change to both of child and parental IBD haplotypes. Figure 1 shows examples of how true recombination events and SEs in parental or child haplotypes lead to changes in the inheritance pattern in terms of 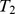, 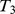 and 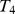 events.

We accommodate genotyping error by allowing for errors in the emission part of the HMM. We model the observed haplotypes at the 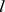
th locus conditional upon the inheritance state 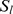 as follows
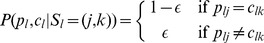
Our full model can then be written down as
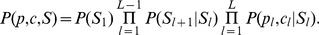
This method can be applied to any set of estimated haplotypes from parent-child pairs. We run one iteration of the Forward-Backward algorithm [36] to estimate 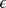, 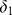 and 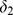. Since there is little uncertainty in the state path this was found to be adequate for convergence. This estimation is carried out on each duo separately. Since the HMM has just four states the computation involved is negligible.

We applied the duo HMM to the Beagle and SHAPEIT2 haplotypes from each cohort and examined the Viterbi paths for each duo. The Viterbi path is the most likely underlying state sequence, given the observed data [36]. We split duos according to the sex of the parent as we know that the rate of recombination events is higher in females than in males [37].

#### Correcting haplotypes

We now describe how to use our model to adjust haplotypes so that they are consistent with a given pedigree structure. After estimating parameters, we run the Viterbi algorithm to find the most likely state sequence. There are sixteen possible state transitions in our model. The eight transitions in the sets 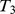 and 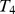 imply a SE in the child haplotypes, so when we observe one of these transitions in the Viterbi sequence we infer a SE in the child. The eight transitions in the sets 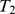 and 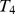 imply either a SE or a recombination event in the parental haplotypes. Inferring whether a recombination or a SE has occurred in the parental haplotypes is difficult. When more than one sibling is present in a pedigree, we can correct probable parental SEs via identifying the minimum recombinant haplotypes for the family. When one of the 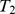 or 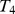 transitions is present in the same location for the majority of siblings, this is most likely a SE on the parental haplotypes and they can be corrected accordingly (see Figure S5 for an example of this process). This is not strictly the maximum-likelihood solution, but the minimum-recombinant and maximum likelihood solutions often yield the same result [38]. When we infer a SE in either a parent or a child we correct the haplotypes by switching the haplotype phase of all loci proceeding the SE. This procedure is carried out left to right along the sequence.

Corrections are applied sequentially ‘down’ through each pedigree. For example, in a three generation (grandparent-parent-child) pedigree we first apply the method to those duos containing grandparents. Any corrections made to the parents haplotypes are used when processing duos involving those parents and their children. This removes any (detectable) SEs for individuals that have parents, before their descendants are phased. We applied our method of correcting haplotypes to all of the chromosome 10 haplotypes produced by SHAPEIT2, Beagle and HAPI-UR in all cohorts and evaluated the improvements in accuracy as well as the number of corrections that were required.

#### Detecting recombination events

Once all the haplotypes have been corrected the duoHMM is re-run in order to infer recombination events. We do this by calculating the probability of a recombination event between markers. A transition between the parental haplotypes corresponds to either a SE or a genuine recombination. A recombination event can only be resolved down to the region between its two flanking heterozygous markers in the parent. We use 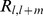 to be the indicator variable of a recombination event between heterozygous markers 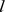 and 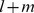. We evaluate the posterior probability of such a recombination event as
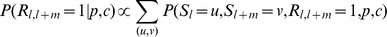
where
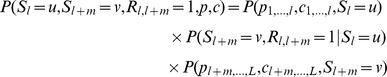
The first and last probabilities can be calculated from the forward-backward algorithm, and the transition rates that include a recombination event are as follows
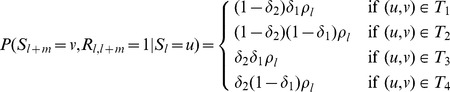
Note since the loci between 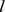 and 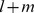 are homozygous the emission probability is the same regardless of state, hence we do not require this term in the calculation. A recombination event is inferred when
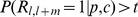
for some threshold 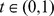. In the analysis in this paper we have used 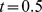. To calculate these probabilities we tried using SHAPEIT2's most likely (pedigree corrected) haplotypes, or sampling haplotypes from SHAPEIT2's diploid graph (again correcting for pedigree structure) and repeatedly calculating recombination probabilities and averaging the resulting maps. We find that both these methods produce similar results (data not shown) and we only report results from the sampling based approach.

We applied this method separately to all the individuals in each of the eight cohorts. We then compared the regions where our HMM detected a recombination event to the recombination locations found in Merlin's output (the Viterbi solution to the Lander-Green algorithm). To reliably detect recombination events, a Lander-Green implementation requires either a nuclear family with at least three children or a three generation pedigree [39], [40]. Hence we only evaluate meioses that meet these criteria in the real data sets, referring to these as *informative* meioses.

#### Detecting genotyping error

We can also use this model to detect genotyping error at locus 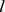, by summing over the posterior probabilities of inheritance states that have inconsistent haplotypes. We use the indicator variable 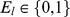 to denote the absence/presence of a genotyping error at locus 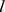 in a duo. Then we have
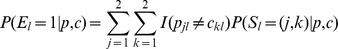
where 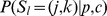 is the posterior probability of inheritance pattern 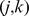 and can be efficiently calculated from the HMM model. This is the probability of a genotyping error occurring in at least one member of the duo given the observed haplotypes. We show on simulated and real data that masking genotypes with 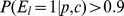 yields a drop in switch error rates suggesting that this is an effective error detection method.

#### Using detected recombinations for association scans of hotspot usage

To demonstrate the utility of our recombination detection method we conducted association testing between genetic variants in the *PRDM9* region (chr5:23007723–24028706) and the “hotspot usage” phenotype described in Coop et al. (2008) [39]. Substantial association in this region was also found in Kong et al. (2010) [12] and Hinch (2011) [41]. We calculated the same phenotype as Coop et al. (2008), the proportion of crossover events, 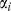, that occur in a recombination hotspot for individual 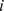 (the parent). This value was corrected for the probability that events occur in one of these hotspot regions by chance via simulation.

The accuracy with which 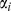 is measured increases with the number of crossovers observed for that parent, hence parents with more observed crossovers should be given higher weighting (large nuclear families are advantageous in this situation). We weighted individuals by creating pseudo-counts of hotspot events 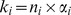 where 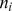 is the number of crossover events observed for parent 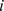. We then fit a standard Binomial Generalised Linear Model (GLM) with 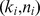 as the response and the genetic dosage at each SNP as the covariate. We then performed a likelihood ratio test between this model of association and the ‘null’ model where no genetic variant is included. Variants were imputed from the 1000 Genomes March 2012 reference panel and filtered such that all variants had 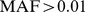 and 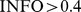 in all cohorts.

The use of the Binomial GLM allows us to leverage parents who are part of typically uninformative meioses, where it is unlikely the majority of crossover events were detected. Such individuals are simply down weighted in our association testing.

### Simulation study

In our real data experiments we use haplotypes inferred by Merlin as the ‘true’ haplotypes for our methods comparison. In the Results section we show that SHAPEIT2 phases extended pedigrees with close to perfect concordance with the haplotypes produced by Merlin (typically 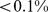 average SE). This level of discordance is of a similar order to both the number of recombination events, and genotyping error [42] which the Lander-Green algorithm is known to be sensitive to. Whilst we have applied standard quality control procedures (including Merlin's error checking) to these data, genotyping errors are likely to still be present. Hence at least some of this discordance may be in fact due to errors in Merlin haplotypes. We also compare the recombination events detected by Merlin in extended families to those detected by our duoHMM approach. Any discordance between these crossover callsets may also be due (in part) to Merlin errors. We also wanted to investigate the ability of our method to call crossover events in duos and trios which cannot be done with the Lander-Green algorithm. For these reasons we created several simulated datasets to investigate these issues.

We utilised male chromosome X haplotypes as the basis for these simulated datasets. Since males only have one copy of chromosome X, phase is unambiguously known. As in previous phasing studies [2], [43], [44], two male X chromosomes were combined to create a pseudo autosomal diploid founder individual where the true underlying haplotypes are known. We then randomly mate these new diploid individuals to produce offspring with recombined haplotypes. Crossover events were simulated as a Poisson process on the genetic lengths from the HapMap Chromosome X genetic map for females and the same map scaled by 0.605 for males (difference in rates estimated from 2002 deCODE Map).

In all experiments, we applied a simple rejection sampling scheme to avoid large amounts of consanguinity in our new diploid individuals and their offspring. The X chromosomes used to create pedigree founders were sampled such that no pair of chromosomes came from pairs of males with genome wide relatedness 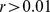 [45]. We conducted these experiments using the 1071 (607 females and 464 males) nominally unrelated individuals from the Val Borbera cohort. This allowed us to create up 232 to diploid individuals with known haplotypes.

#### A simulated dataset with extended pedigrees

We wished to investigate accuracy on pedigrees that are collected as part of a larger cohort, hence we simulated pedigrees with the same structures as those observed in the Val Borbera cohort. We only used those pedigrees having “informative” meioses (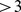 siblings or three generations) and generated founder individuals using male X chromosome data. These simulated founders were then “mated” to create descendants (and the descendants were also mated in cases of three generation pedigrees). There were 65 such pedigrees, with 199 founders although only 108 of these founders were assayed. We carried out two sets of simulations: one realistic scenario where we attempt to emulate the type of data collected in practice and one ideal scenario which should be advantageous for Merlin.

In the realistic scenario we simulated genotyping errors based on a confusion matrix (Table S2) from a previous study [42], that was estimated by comparing genotypes called on both an Affymetrix Axiom chip and a Illumina Omni 2.5S chip on 1000 Genomes individuals. There were many cases where founders were missing in practice. We also removed the 91 ungenotyped founders leaving 108 genotyped founders and a total of 314 individuals in pedigrees. Finally these extended pedigrees were merged with the 607 female chromosome X data (and the remaining 33 pseudo-autosomal males not in a pedigree) to create a cohort of 954 individuals containing 314 individuals in pedigrees and 640 unrelated individuals. We created 10 versions of this simulated dataset. In the ideal scenario we do not add any genotyping error and do not remove founders (leaving all 199 founders in the data giving us a data set with 1045 individuals (405 within a pedigree).

We use the realistic dataset to compare three different versions of our method for phasing extended pedigrees. First we applied SHAPEIT2 ignoring all pedigree information. Secondly, we applied our duoHMM method to this set of haplotypes to correct SEs. We also used the duoHMM output to identify positions where there was strong evidence of genotyping error, and we investigate the effect of excluding these sites for accuracy comparisons. Finally, we applied our method of partitioning the extended pedigrees into trios and duos and then ran Beagle using this level of family information. We also evaluate Merlin's haplotype accuracy on this simulated data.

We also ran Merlin and our duoHMM method on these datasets to detect recombination events on all informative meioses which allowed us to investigate the sensitivity and specificity of the methods in both a realistic and ideal scenario. In the realistic and ideal simulations, there were 183 and 212 informative meioses containing a total of 2422 and 3131 crossover events (across all simulations) on which to evaluate accuracy.

#### A simulated dataset of uninformative duos

The ability to detect recombination events is enhanced if an individual is part of a large pedigree. Such pedigrees will contain parent-child relationships where the parent's heterozygous sites can be phased independently of that child's loci (such as from a grandparent or another child) this allows changes in the pattern of inheritance (recombination events) to be detected. Previous work on detecting recombination events in pedigrees have used such informative pedigrees [37], [39], [41], [46]. We wanted to assess the power of our method to detect recombination events in uninformative duos.

We created a simulated dataset of 116 mother-father-child trios using the 464 chromosome X haplotypes from the Val Borbera cohort. These trios were merged with the diploid female chromosome X data to increase sample size.

We created a second version of this simulated dataset by first removing individuals from the Val Borbera dataset such that no pair of individuals had a genome wide relatedness 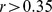 in the remaining data. This reduced the dataset from 1071 to 778 individuals (440 females and 338 males) allowing us to simulate a dataset with 84 mother-father-child trios, merged together with the original 440 females. By removing closely related individuals from the cohort we create a simulated dataset more like a non-isolated population. Detection of recombination events will be harder in this setting due to reduced levels of IBD sharing.

The merging, mating and recombination was simulated ten times (each simulation analysed separately) creating 2270 and 3190 recombination events to evaluate sensitivity and specificity of our method. We then attempted to detect the recombination events using the pipeline previously described in the Methods section.

## Results

### Levels of relatedness within each cohort

**Figure 2:**
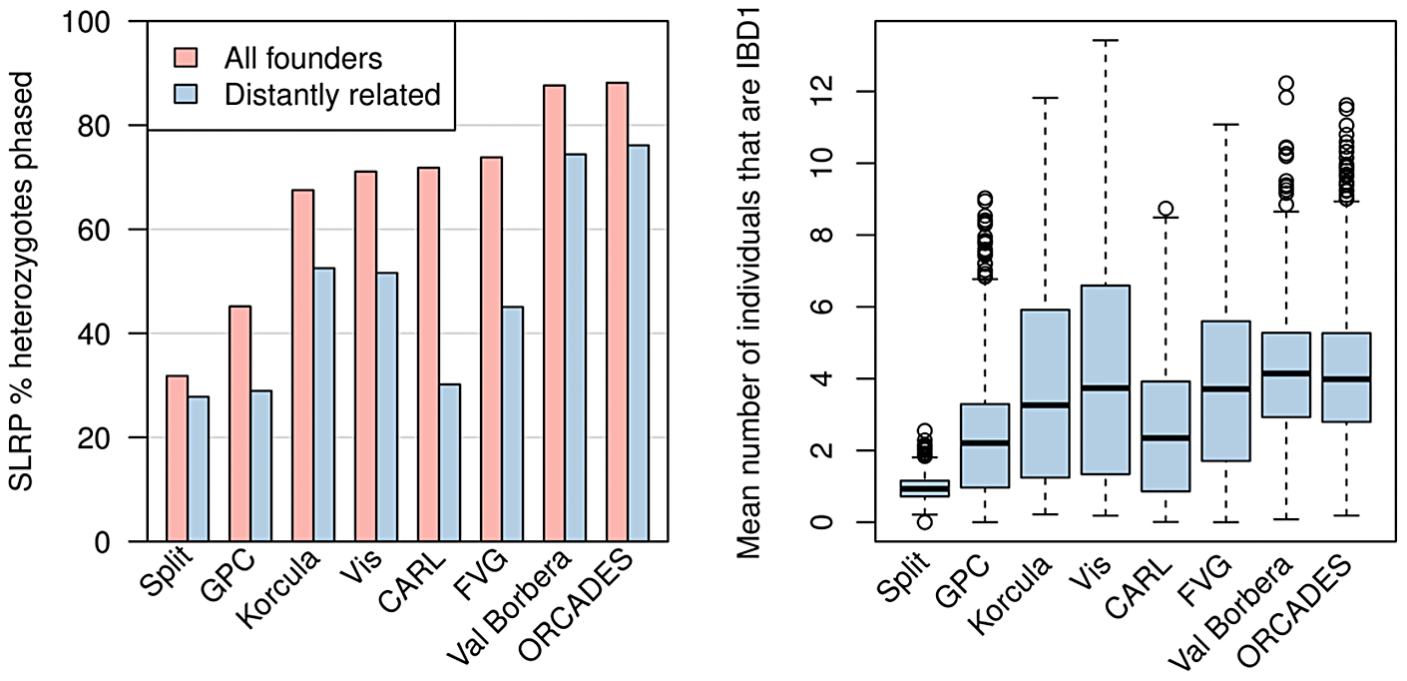
Summary of IBD sharing in cohorts. Left: The proportion of heterozygote sites phased by SLRP for all individuals (pink) and when individuals with close relatives (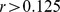) are removed (blue). Right: The distributions of the average number of “surrogate” parents for each cohort when closely related pairs (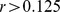) are removed.

Figure 2 (left) shows the proportion of heterozygote sites phased by SLRP, which we refer to as the yield. SLRP's yield ranged from 31.82% for the Split cohort to 88.15% for the ORCADES cohort. Split and GPC were the only cohorts with less than 60% yield demonstrating low levels of IBD sharing between individuals in these cohorts. Following Palin et al. [13] we also examined individuals who were not “closely” related by excluding all individuals with a realised relatedness [45] of 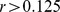. We found the yield was substantially lower in the CARL and FVG cohorts demonstrating some of the IBD sharing present was between closely related individuals rather than distant cousins in these cohorts. All other cohorts did not exhibit as large a drop in yield after removing closely related individuals, highlighting the large amounts of IBD sharing between more distantly related individuals in these cohorts.

Similar to Kong et al. (2008) [12], we took each individual in turn and at each locus we calculated the number of other individuals that share an IBD segment (excluding closely related individuals with relatedness 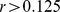). We then took the average across all loci on chromosome 10 for each individual and plotted the distribution of this average IBD sharing in Figure 2 (right). The average IBD sharing is a function of both the sample size and the amount of relatedness between individuals in the population. Split again clearly has very small amounts of IBD sharing whilst the other cohorts have broadly similar distributions. It is notable that all cohorts have some individuals with 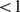 surrogate parent on average while some individuals have 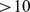 surrogate parents.

### Haplotype accuracy in founder individuals

Table 1 shows the SE rates for SHAPEIT2, SLRP, Beagle and HAPI-UR when run on the founder individuals of each cohort, both within and outside SLRP IBD segments. SHAPEIT2 consistently produced the most accurate haplotypes of all methods within IBD regions. SHAPEIT2 had a mean SE rate of between 0.14% (ORCADES) and 0.75% (CARL), the next closest method was SLRP with SE rates between 0.28% (GPC) and 1.99% (Split), followed by Beagle with SE rates between 0.30% (GPC) and 4.38% (Split). HAPI-UR had high SE rates ranging between 0.35% (GPC) and 8.30% (Split). The GPC cohort stands out here, as all methods perform very accurately (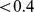
% SE) which can be explained by the larger sample size of this cohort (2,676 individuals in total) and the much denser chip (Illumina Human OMNI2.5S) used to genotype this cohort. It is interesting that SHAPEIT2 seems to have the highest SE rates on the CARL cohort, which has only the second lowest level of relatedness between founders (Figure 2 (right)). Figure S6 (left) shows the SLRP IBD SE rate against the SHAPEIT2 rate for each individual in the Val Borbera cohort. This highlights that both methods produce SE rates close to zero on many individuals but SHAPEIT2 is generally more accurate.

**Table 1:** Switch error rates for samples containing nominally unrelated individuals.

Cohort	CARL	FVG	GPC	KOR	ORC	SPL	VB	VIS
Chip	370K	370K	2.5S	370K	300K	370K	370K	300K
N validation individuals	130	274	419	118	201	50	481	150
SLRP Yield	71.82	73.82	45.17	67.52	88.15	31.82	87.63	71.09
SHAPEIT2 (IBD)	0.75	0.21	0.16	0.18	0.14	0.60	0.17	0.19
SLRP (IBD)	1.15	0.40	0.28	0.45	0.33	1.99	0.33	0.43
Beagle (IBD)	2.57	1.18	0.30	1.25	0.94	4.38	1.07	1.30
HAPI-UR 3× (IBD)	5.43	2.25	0.35	2.55	2.59	8.30	1.93	2.94
SHAPEIT2 (No IBD)	5.03	4.10	0.51	3.11	2.43	3.35	2.74	3.65
Beagle (No IBD)	7.73	5.72	0.84	5.55	5.05	6.00	5.03	6.36
HAPI-UR 3× (No IBD)	15.97	10.31	0.82	10.20	9.94	12.09	7.92	12.47
SHAPEIT2	2.85	1.81	0.28	1.05	0.49	2.65	0.62	1.16
Beagle	5.31	3.11	0.49	2.53	1.78	5.57	1.88	2.78
HAPI-UR 3×	11.21	5.83	0.50	4.82	4.20	11.10	3.21	5.77

All individuals not explicitly related in the defined pedigrees were phased. We calculate overall SE rate (All), for chromosome 10 (this is not possible for SLRP) as well as SE rates within (IBD) and outside (no IBD) SLRP detected IBD regions. SHAPEIT2 consistently produces the most accurate haplotypes. Chip abbreviations: 370K - Illumina HumanHap 370CNV. 300K - Illumina HumanHap 300, 2.5S - Illumina Omni 2.5S.

When calculating SE rate across the whole of chromosome 10 (not just in IBD regions), SHAPEIT2 also has the lowest error rate, ranging from 0.28% (GPC) to 2.65% (Split cohort) as opposed to 0.49% and 5.57% for Beagle and 0.50% and 11.10% for HAPI-UR. Switch error rates for SLRP cannot be evaluated across the whole of chromosome 10 due to the method only producing partially phased haplotypes. We observe that *all* methods perform relatively better within IBD regions than across the whole chromosome. However, the difference for SHAPEIT2 is much larger than for Beagle and HAPI-UR. These results suggest that whilst none of these methods explicitly model IBD sharing, its presence tends to be exploited implicitly, and particularly so in SHAPEIT2. Figure S6 (right) plots the SHAPEIT2 SE rate *within* IBD regions (detected by SLRP) against the rate *outside* these regions. Switch error is clearly close to zero when IBD sharing is present and has a rate more comparable to non-isolated populations when no IBD sharing is present.

### Haplotype accuracy in extended pedigrees

#### Treating all individuals as unrelated

Table 2 shows the SE rates for SHAPEIT2, SLRP, Beagle and HAPI-UR when run on all individuals in each cohort and ignoring any of the information about explicit family relationships between individuals. We calculated SE for each method on the haplotypes of any individual within an extended pedigree more complex than a simple duo or trio, using the Merlin haplotypes as truth.

**Table 2:** Switch error (SE) rates for different methods applied to extended pedigrees.

Cohort	CARL	FVG	GPC	KOR	ORC	SPL	VB	VIS
Chip	370K	370K	2.5S	370K	300K	370K	370K	300K
N validation individuals	228	460	616	118	300	38	739	154
SLRP Yield	90.870	96.094	72.576	96.319	98.344	81.523	98.613	97.818
SHAPEIT2 (IBD)	0.209	0.086	0.155	0.058	0.066	0.090	0.073	0.075
SLRP (IBD)	0.683	0.319	0.284	0.486	0.301	1.075	0.387	0.116
Beagle (IBD)	1.211	0.825	0.253	0.970	0.353	4.073	0.492	0.923
HAPI-UR 3× (IBD)	2.773	1.445	0.293	1.755	1.072	7.599	0.860	1.952
SHAPEIT2 (No IBD)	0.768	0.424	0.305	0.105	1.217	0.112	0.423	0.162
Beagle (No IBD)	3.893	3.338	0.626	3.211	3.741	4.293	2.462	3.673
HAPI-UR 3× (No IBD)	8.150	5.698	0.709	6.186	5.898	8.747	3.544	7.233
SHAPEIT2	0.241	0.097	0.166	0.059	0.083	0.093	0.078	0.076
Beagle	1.362	0.907	0.279	1.034	0.403	4.096	0.516	0.973
HAPI-UR 3×	3.074	1.582	0.322	1.880	1.141	7.722	0.892	2.049
SHAPEIT2+duoHMM	0.232	0.092	0.160	0.058	0.079	0.091	0.073	0.073
Beagle+duoHMM	0.934	0.643	0.234	0.789	0.255	3.200	0.318	0.703
HAPI-UR 3×+duoHMM	2.134	1.108	0.263	1.415	0.640	6.193	0.502	1.494
Beagle Duo/Trio	0.445	0.265	0.166	0.113	0.151	0.595	0.175	0.129
Masked SHAPEIT2+duoHMM	0.088	0.056	0.149	0.047	0.052	0.060	0.045	0.060
Masked Beagle Duo/Trio	0.360	0.238	0.157	0.111	0.146	0.584	0.165	0.127

We evaluate SE for individuals who are members of a complex pedigree (pedigrees that are larger than a parent-child duo and father-mother-child trio). The first row is the number of individuals from each cohort in such pedigrees. The second row shows the yield of SLRP when applied to each cohort. Rows 3–6 show the SE for SHAPEIT2, SLRP, Beagle and HAPI-UR within SLRP detected IBD regions. Rows 7–9 show the SE for SHAPEIT2, Beagle and HAPI-UR outside SLRP detected IBD regions. Rows 10–12 show the overall SE for SHAPEIT2, Beagle and HAPI-UR. Rows 13–15 show the overall SE for SHAPEIT2, Beagle and HAPI-UR haplotypes after correction with the duoHMM method. Row 16 show the overall SE for Beagle applied to pedigrees partitioned into duos and trios where possible. Rows 17–18 show the switch error rate for the SHAPEIT2+duoHMM and Beagle Duo/Trio phasing *after* masking genotypes flagged as erroneous by the duoHMM.

All of the methods have lower SE rates than when run on just the founders of the pedigrees (Table 1) but the improvement for SHAPEIT2 is most striking. We find that SHAPEIT2 achieves between 0.059% (Korcula) and 0.241% (CARL) SE rate on individuals within pedigrees. SLRP achieves a very high yield in most cohorts (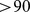
% except in Split and GPC) for individuals within pedigrees and improved accuracy within the IBD regions it detects. Both Beagle and HAPI-UR (3×) are also more accurate in this setting as well but do not obtain the same gains as SHAPEIT2.

#### duoHMM corrected haplotypes

**Figure 3:**
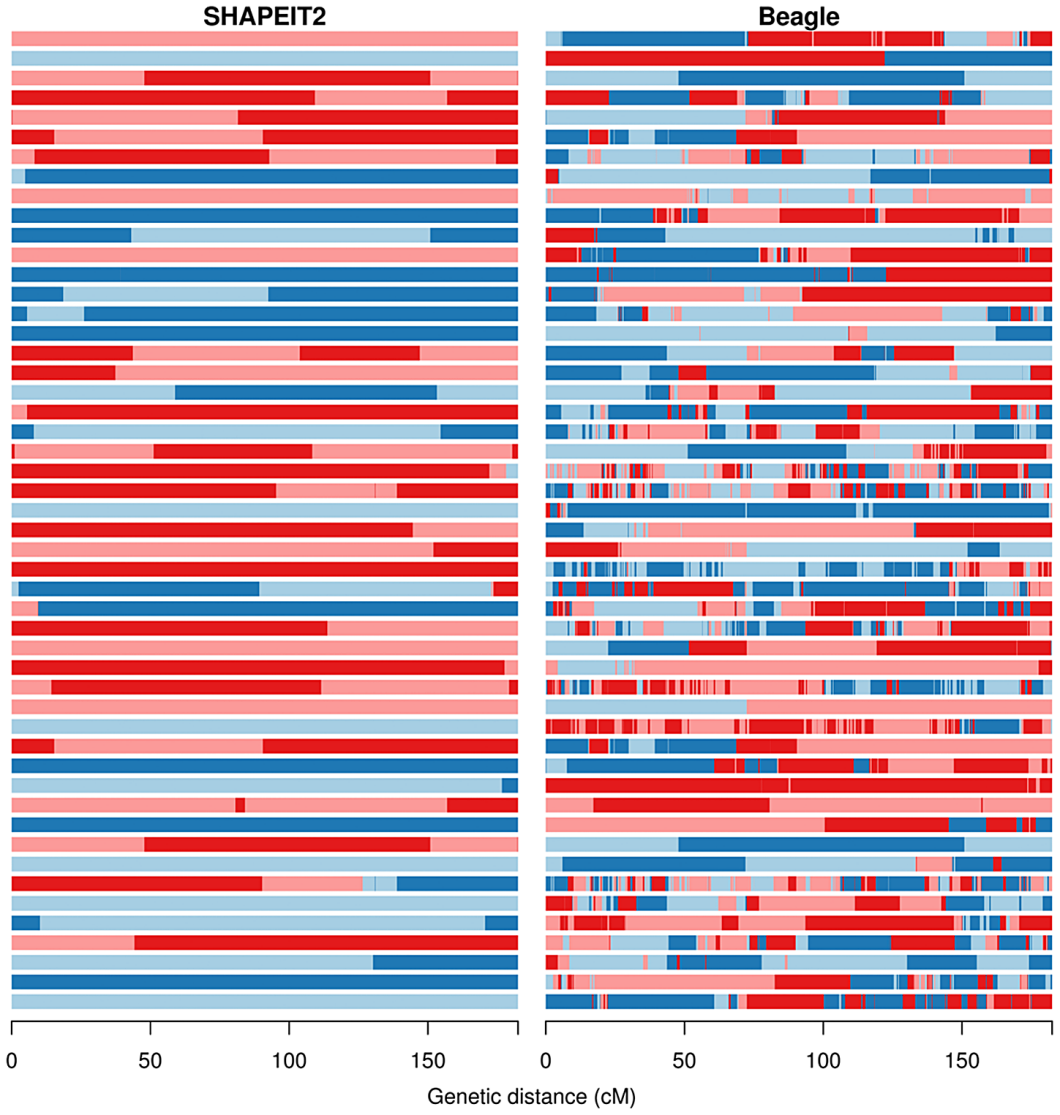
The duo HMM Viterbi paths for 50 father-child duos from the Val Borbera cohort on chromosome 10. The four possible IBD states (A, B, C, D) are shown using colours pale blue, dark blue, light red and dark red respectively. The left and right panels show the results of the duo HMM applied to the SHAPEIT2 and Beagle haplotypes respectively. Changes between a blue and red colour correspond to a 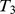 or 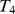 transition, both of which imply a SE in the child. Changes of colour between light and dark blue or between light and dark red correspond to 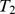 transitions, which correspond to a change on IBD state in the parent, and could be caused by a recombination or a SE in the parent. The x-axis shows the sex-averaged genetic distance across the chromosome in centiMorgans.

We applied our duoHMM method to the SHAPEIT2, Beagle and HAPI-UR haplotypes that were estimated ignoring all family information. To give a sense of the output of applying this model Figure 3 shows the Viterbi paths of 50 male parent duos from the Val Borbera cohort for both SHAPEIT2 and Beagle. The four possible IBD states 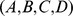 are shown using colours pale blue, dark blue, light red and dark red respectively. Changes between a blue and red colour correspond to a 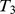 or 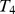 transition, both of which imply a SE in the child. Changes of colour between light and dark blue or between light and dark red correspond to 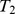 transitions, which correspond to a change on IBD state in the parent, and could be caused by a recombination or a SE in the parent. Figure 1 provides examples that can be helpful in interpreting Figure 3. Figures S7, S8, S9, S10, S11, S12, S13, S14, S15, S16, S17, S18, S19, S20, S21, S22 show the Viterbi paths of male parent and female parent duos for all of the cohorts.

Figure 3 shows a striking difference between the output on the SHAPEIT2 and Beagle haplotypes. The SHAPEIT2 paths have very few transitions of all types, and when transitions occur they are predominantly 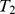 transitions. The figure shows only father-child duos and chromosome 10 has been estimated to have a genetic length of 1.34 Morgans for paternal meioses [37]. The numbers of 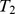 transitions in the 50 duos in Figure 3 looks reasonably consistent with this genetic length, suggesting that the 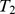 transitions are indeed true recombination events. We note that there are some duos with no transitions. This is a possible outcome of a meiosis and is more likely to occur on the shorter chromosomes, and can also be the product of undetected recombination events.

The Beagle haplotypes contain many more 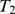, 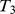 and 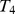 transitions. In the Val Borbera cohort when we compared the SHAPEIT2 and Beagle haplotypes to those estimated by Merlin we found that SHAPEIT2 produced 4,613 SEs in 1,074 individuals corresponding to 4.3 switches per individual, whereas Beagle produced 29,681 switches or 27.6 switches per individual. These numbers seem consistent with what we observe in Figure 3. The higher rate of SEs in the Beagle haplotypes cause a large number of changes in estimated IBD state.

Table 3 shows the mean number of state transitions in paternal and maternal duos for each cohort for SHAPEIT2 and Beagle. Note that 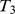 and 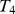 transitions are biologically impossible as they represent a change in which child haplotype the parent is transmitting genetic material to. The SHAPEIT2 haplotypes typically have 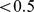 of these transitions occurring per duo whilst Beagle ranges from 7.49 to 97.17 for 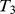 transitions.

**Table 3:** Summary of DuoHMM state transitions for each cohort.

			SHAPEIT2						Beagle					
	Duos		Paternal			Maternal			Paternal			Maternal		
	P	M	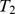	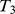	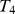	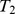	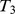	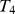	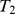	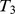	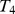	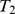	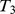	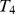
CARL	72	120	1.47	0.26	0.00	2.73	0.72	0.07	34.35	24.19	0.83	50.99	25.09	1.07
FVG	163	289	1.29	0.26	0.02	2.25	0.42	0.03	30.17	19.23	0.73	27.06	13.99	0.43
GPC	228	462	2.99	1.37	0.02	4.26	1.64	0.04	11.79	10.31	0.17	16.95	10.10	0.15
KOR	49	104	0.71	0.04	0.00	1.15	0.00	0.00	32.84	24.35	1.24	42.68	25.52	1.38
OR C	148	187	0.90	0.00	0.00	2.00	0.04	0.00	9.16	9.49	0.31	11.41	7.49	0.22
SPL	18	35	0.17	0.00	0.00	1.20	0.00	0.00	122.33	97.17	6.17	128.20	88.69	6.54
VB	303	479	1.46	0.31	0.02	2.25	0.38	0.05	13.89	14.82	0.33	15.35	13.53	0.32
VIS	68	130	1.29	0.16	0.03	2.07	0.49	0.02	19.74	18.56	0.60	43.25	15.15	0.72

The mean number of switches occurring (excluding 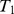) found by the Viterbi path through our four state HMM for SHAPEIT2 and Beagle maximum likelihood haplotypes for chromosome 10 for paternal (P) and maternal (M) duos. SHAPEIT2 has very few impossible transitions (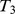 and 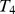) and the number possible recombinations (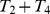) are much closer to the genetic length of chromosome 10 than Beagle. The 2002 deCODE map gives the chromosome 10 genetic length as 1.34 and 2.18 Morgans for males and females respectively.

Transitions 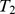 and 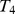 may correspond to crossover events or SEs in the parental haplotypes. The 2002 deCODE map gives the chromosome 10 genetic length as 1.34 and 2.18 Morgans for males and females respectively [37]. The mean numbers of 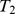 or 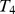 transitions in the SHAPEIT2 show rough agreement with the expected number of recombinations on chromosome 10 in most cohorts. For example, in the VIS cohort we observe 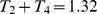 and 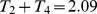 transitions in males and females respectively. The female rates in CARL are higher than we might expect at 2.8. Both the male and female rates are lower than we might expect in the Korcula and Split cohort. This is likely due to insufficient information in the data to infer the true parental haplotype and hence we are seeing a parental SE at the recombination event, that is, we are inferring the *transmitted* haplotypes.

Figure S5 shows the results of applying our duo HMM on the SHAPEIT2 haplotypes of all father-child duos in a three sibling family before and after using our correction method. Before correction (Figure S5 (left)), the first father-child duo exhibits no evidence of recombination (change in colour between dark and light red) and these two individuals share a whole haplotype across the whole chromosome. The other father-child duos show evidence of 3 and 4 recombination events respectively, with one event being shared in common at 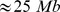. Our method infers a parental SE at this location. This has the effect (Figure S5 (right)) of reducing the total number of inferred recombination events across the 3 duos from 0, 3 and 4 to 1,2 and 3, which seems more realistic.

Table 2 shows the mean SE rate after applying haplotype corrections. The corrections lead to a consistent but small improvement for the SHAPEIT2 haplotypes, the largest improvement being a 0.009% decrease in SE for the CARL cohort. These results further highlight the very high quality haplotype estimates that SHAPEIT2 produces despite the fact it is ignoring all explicit pedigree information.

We also find the duoHMM method is beneficial to Beagle and HAPI-UR when used to correct those haplotypes. For example the SE rate for HAPI-UR drops from 7.722% to 6.193% for the Split cohort. Table S3 gives the average number and type of correction applied to each cohort for each method. SHAPEIT2's haplotypes require less than 0.5 corrections on average for chromosome 10, this is consistent with the very small improvement in SE.

#### Partitioning pedigrees into Duos/Trios

Table 2 also shows the haplotype accuracy for pedigrees that were partitioned into duos and trios and then phased accordingly using Beagle (denoted as Beagle Duo/Trio). Not surprisingly, adding the duo/trio relationships yields a substantial improvement to the Beagle haplotypes across all cohorts; for example we see a drop from 1.362% SE to 0.445% SE in CARL. However they are still consistently less accurate than SHAPEIT2's haplotypes (even though SHAPEIT2 is not using any relationships).

**Figure 4:**
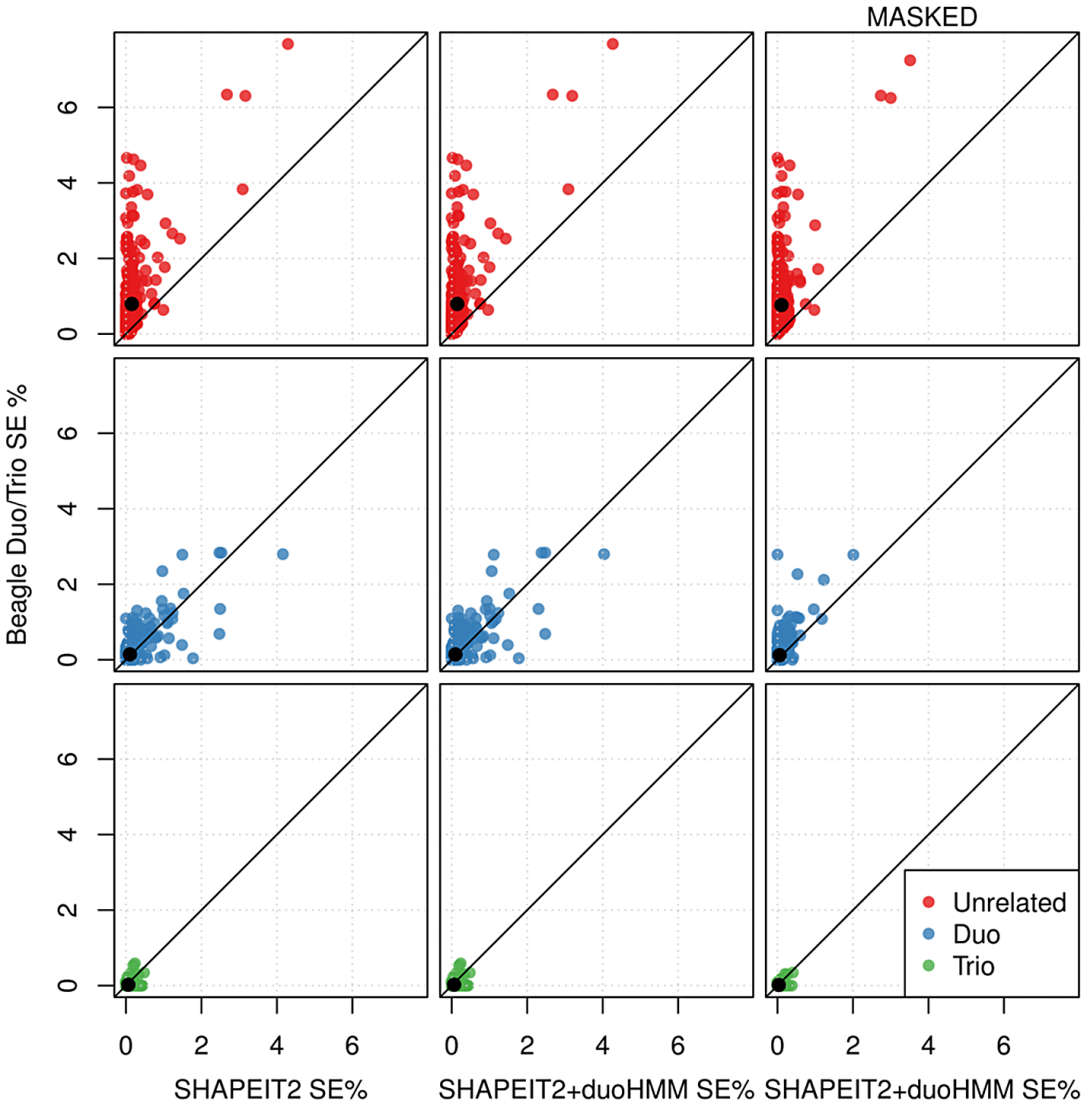
Switch error rates for individuals in extended pedigrees for different phasing pipelines across all European cohorts (chromosome 10). Points are coloured according to what relationship was used by Beagle to phase that individual (red meaning no relationships were used). Left: Beagle using duo/trio phasing versus SHAPEIT2 using no relationships. Centre: Beagle using duo/trio phasing versus SHAPEIT2+duoHMM using no relationships. Right: Beagle using duo/trio phasing versus SHAPEIT2+duoHMM using no relationships when masking loci flagged as probable genotyping errors by the duoHMM. Switch error is reduced for both methods suggesting the masking is sensible.

When using the Beagle Duo/Trio method some individuals will not be phased as part of a duo or trio, for example one of the children in a three sibling nuclear family. Figure 4 (top and centre left) plots the SE for such “unrelated” individuals for Beagle Duo/Trio versus SHAPEIT2 and Beagle Duo/Trio versus SHAPEIT2+duoHMM respectively. These plots show that these individuals are phased much more accurately using our methods. Duos and trios are phased almost equally well by all methods.

We used our SHAPEIT2+duoHMM method to flag sites of possible genotyping error and then re-calculated the SE rates for SHAPEIT2+duoHMM and the Beagle Duo/Trio method excluding these sites. The results are shown in the last two rows of Table 2. Comparing these results to the unmasked results we see that the reduction in SE is greatest for SHAPEIT2+duoHMM, suggesting that genotyping error causes the results of Beagle Duo/Trio to appear better than they are. The results from our simulation study (described next) corroborate this point. Figure 4 (right) shows in more detail the effect of masking genotyping errors, and that SHAPEIT2+duoHMM outperforms Beagle Duo/Trio for individuals that were phased as ‘unrelated’ by Beagle.

#### Results on the simulated dataset with extended pedigrees

The simulated pedigree data allows us to evaluate the confounding effect of genotype errors in the Merlin and duo/trio phased haplotypes.

Figure S23 (top right) plots the SE of SHAPEIT2+duoHMM versus Merlin on simulated data with realistic levels of genotyping error. SHAPEIT2+duoHMM is generally more accurate (average of 0.033% versus 0.215% for Merlin on sites resolved by Merlin). Without any genotyping error (Figure S23 bottom right) the performance is much improved for Merlin (SHAPEIT2+duoHMM SE = 0.005%, Merlin = 0.021%).

Figure S24 plots the SE rates on the simulated pedigrees for Beagle Duo/Trio SE rates of all individuals versus those of SHAPEIT2 (left) and SHAPEIT2+duoHMM (centre). The plot shows that the most accurate haplotypes are attained by the SHAPEIT2+duoHMM approach, and that the duo/trio constrained phasing can be susceptible to genotyping error. The SE rates of SHAPEIT2, SHAPEIT2+duoHMM and Beagle Duo/Trio were 0.104%, 0.065% and 0.269% respectively. When we removed sites flagged as genotyping errors by our method the SE rates were 0.073%, 0.034% and 0.231% respectively, suggesting that the masking can remove switch errors caused by genotyping errors.

Overall, these results suggest that at least some of the observed discordance in the analysis of real data sets is due to errors in the Merlin haplotypes caused by genotyping error. The true error levels are likely to be lower but the masking can help to remove the confounding effect.

### Detecting recombination events

#### Informative pedigrees

We evaluated the sensitivity and specificity of our recombination detection routine as well as Merlin on our simulated data. When Merlin flags a crossover event between two markers, we extend the region this event may have occurred to the two flanking (phase resolved) heterozygous markers on the relevant parent. Our DuoHMM routine already produces the probability of a crossover event occurring between adjacent heterozygous markers on a parent. If an event occurs within one of these regions it is correctly detected, if not the flagged region is a false positive.

Figure S23 shows the true positive rate (TPR) against the false discovery rate (FDR) for 2422 realistic (3131 ideal) simulated crossover events from 1830 (2120 ideal) informative meioses. In the simulations with realistic levels of genotyping error (Figure S23 top left), Merlin detects 90.57% of events but with a substantial FDR of 62.48% while SHAPEIT2 has a FDR of 2.89% at the same rate of detection. SHAPEIT2 has the additional advantage of having a probability associated with each event, which can be thresholded. For example, by setting a threshold of 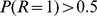 we can achieve a FDR = 3.78% and TPR = 92.4% or 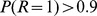 for FDR = 0.58% and TPR = 69.45%. In the absence of genotyping error (Figure S23 bottom left) the performance of Merlin is improved (FDR = 3.48% and TPR = 95.66%) but SHAPEIT2 is marginally better (FDR = 0.71% and TPR = 95.75% at 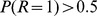).

**Figure 5:**
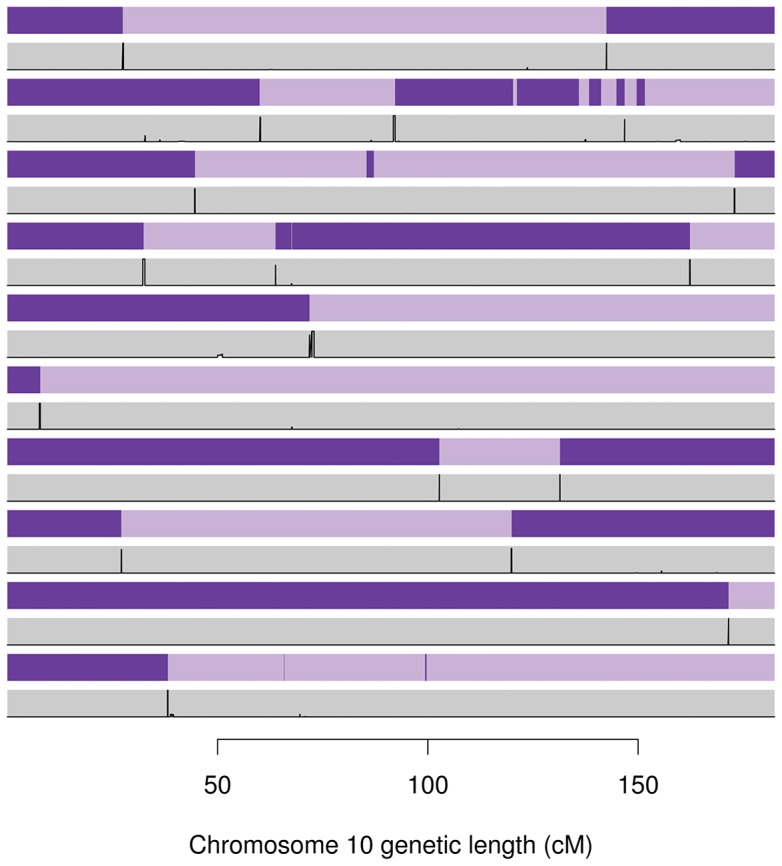
Inferred gene flow by Merlin (purple) and our method (grey) for ten informative meioses on chromosome 10 taken from Val Borbera cohort pedigrees. The light and dark purple represent genetic material from the grand-paternal and grand-maternal chromosomes (as inferred by Merlin's Viterbi algorithm), hence changing from light to dark implies a a recombination event. The grey rectangles contain the posterior probability (in black) of recombination from our method. The two methods broadly agree, although Merlin has inferred a number of implausibly small cross over events.

Figure 5 compares the gene flow (and hence recombination) inferred by Merlin and the recombination probabilities inferred by our method for 10 informative meioses between parent-child duos from the Val Borbera cohort on chromosome 10. This figure shows very good agreement between our estimated probabilities and the events inferred by Merlin. However, these examples highlight that Merlin does infer some rather implausible, sporadic events in some meioses (even after running Merlin's error detection).

Table 4 reports the percentage of Merlin recombinations that fall within a recombination region found by our method, and the percentage of our recombination regions that contain a Merlin recombination event. Percentages are given for each cohort separately. These results show only a rough concordance between Merlin and our method, with between 41% and 61% of Merlin recombinations detected by SHAPEIT2 and 80% to 89% of SHAPEIT2's recombination events concordant with Merlin. This table also shows the mean number of recombination events found by each method in maternal and paternal meioses. For comparison, the frequently cited deCODE 2002 genetic map estimated an average of 25.9 and 42.81 autosomal recombinations per paternal and maternal meioses respectively. The average number of recombinations for Merlin was substantially inflated across most cohorts (53 to 105 for maternal and 31 to 79 for paternal events) whilst SHAPEIT2's were in a more reasonable range (25 to 29 for paternal events and 41 to 47 for maternal events). Figure S25 plots the number of events found per meiosis by SHAPEIT2+duoHMM versus Merlin; there is obvious correlation but Merlin is typically reporting a much larger number of events than SHAPEIT2+duoHMM.

**Table 4:** Comparison of recombination detection using our method and Merlin for all informative paternal (P) and maternal (M) meioses events in each cohort.

	Meioses		SHAPEIT2 concordance (%)	Merlin concordance (%)	Merlin		SHAPEIT2	
Cohort	P	M			P	M	P	M
CARL	24	47	80.40	41.28	63.08	74.57	25.46	41.81
FVG	50	96	88.97	60.65	34.90	61.04	25.02	42.23
GPC	69	75	88.23	36.60	79.32	105.48	29.22	47.07
KOR	4	11	80.59	57.16	39.75	63.64	28.75	44.82
ORC	40	45	86.33	50.89	30.85	85.11	25.73	43.47
VB	72	104	88.72	52.26	55.19	61.07	25.17	40.91
VIS	12	14	82.81	58.84	43.75	52.57	26.83	41.00

SHAPEIT2 concordance is the percentage of SHAPEIT2 crossover events that intersected a Merlin crossover event, the proceeding column is vice versa. We also provide the mean number of paternal/maternal recombination events detected for informative meioses by Merlin and SHAPEIT2. For comparison, the frequently cited deCODE 2002 genetic map estimated an average of 42.81 and 25.9 autosomal recombinations per paternal and maternal meioses respectively. SHAPEIT2's estimates are consistently closer to the deCODE values which are considered to be of high quality.

**Figure 6:**
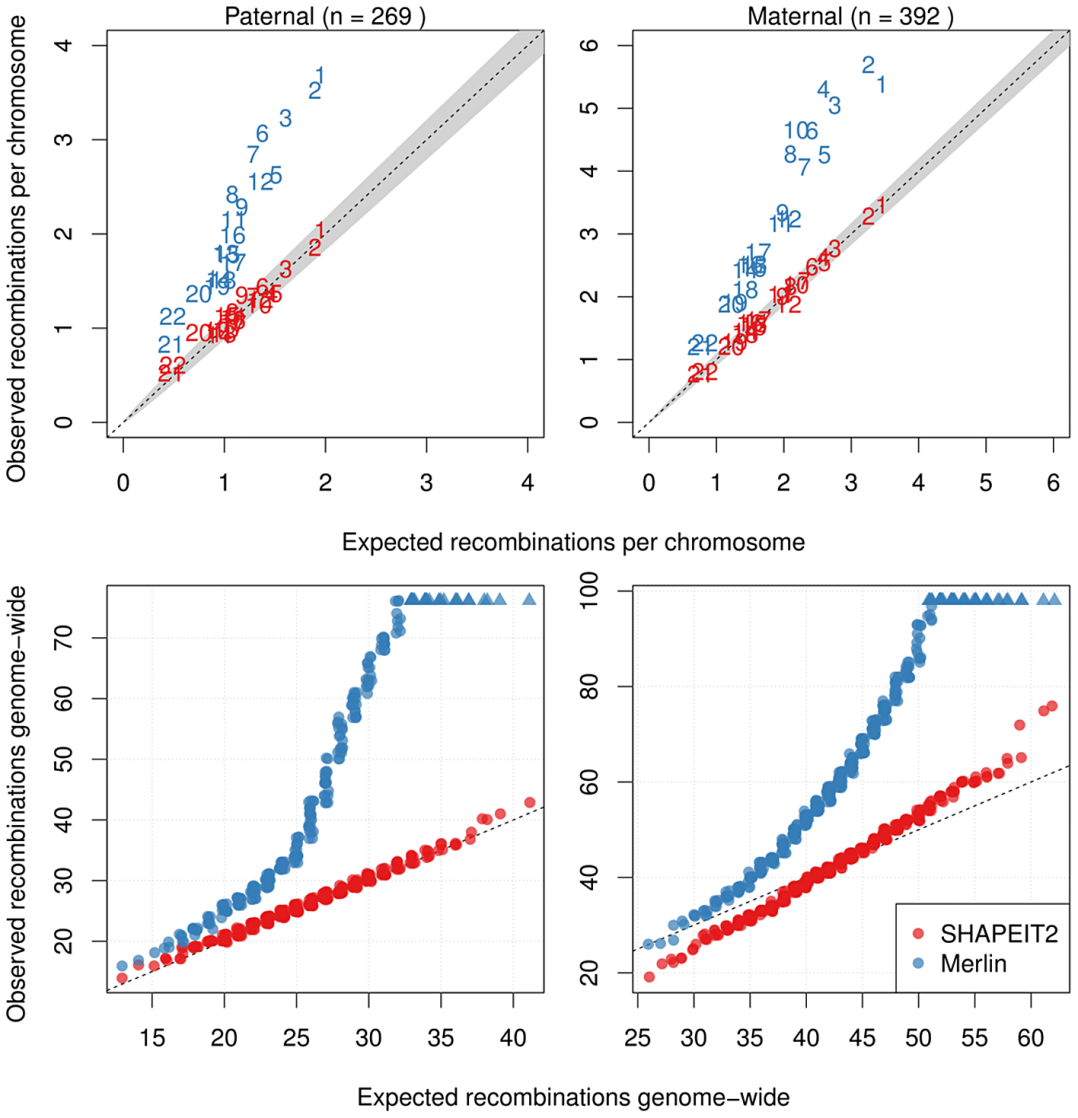
Distributions of the number of detected crossovers for all cohorts. Only duos that were part of an informative pedigree were used. Top: The mean number of recombinations per meiosis (for all informative duos from all cohorts) found for each chromosome against the expected number (from the 2002 deCODE map) for paternal meioses (left) and maternal meioses (right). Merlin's values are substantially inflated whilst SHAPEIT2's are more consistent with the well known deCODE map genetic lengths. Bottom: Q-Q plots for the observed against expected number of recombinations estimated by each method for paternal meioses (left) and maternal meioses (right). For the expected distribution of recombination rates, a Poisson distribution using the genetic lengths from the 2002 deCODE Map was used (with rate parameter 42.81 and 25.9 for maternal and paternal recombinations respectively). SHAPEIT2's rates are less inflated than those of the Merlin.

Figure 6 compares the distribution of the number of recombination events found by Merlin and our method to what we would expect according to the 2002 deCODE family based map. Figure 6 (top) plots the observed against expected number of recombinations in paternal and maternal meioses for each chromosome. The results from SHAPEIT2 are well calibrated against the expectation, whereas the Merlin haplotypes exhibit elevated levels of recombination. Figure 6 (bottom) shows QQ-plots comparing the observed and expected number of genome-wide recombinations in paternal and maternal meioses for each duo when using a Poisson distribution with rates 25.9 (paternal) and 42.81 (maternal) as our expected distribution. SHAPEIT2 rates are well calibrated against the expectation for paternal meioses, with some over-dispersion present in maternal meioses compared to the Poisson model. Figures S26, S27, S28, S29, S30, S31, S32 show these QQ-plots for each of the other cohorts separately.

Figure S33 shows the average fine-scale recombination rate as a function of distance from the inferred recombination events inferred by both SHAPEIT2 and Merlin (black), only SHAPEIT2 (red) and only Merlin (blue). The distribution of recombination rates around the SHAPEIT2-only crossovers is close to the distribution of crossovers found by both methods, whilst the recombination rates near Merlin events are on average lower.

These results on both simulated and real data point to elevated false discovery rates for recombination detection with Merlin, corroborating what has previously been reported in the literature [39] whereas the SHAPEIT2+duoHMM method can constrain FDR whilst still detecting a substantial proportion of true crossover events.

#### Uninformative duos

**Figure 7:**
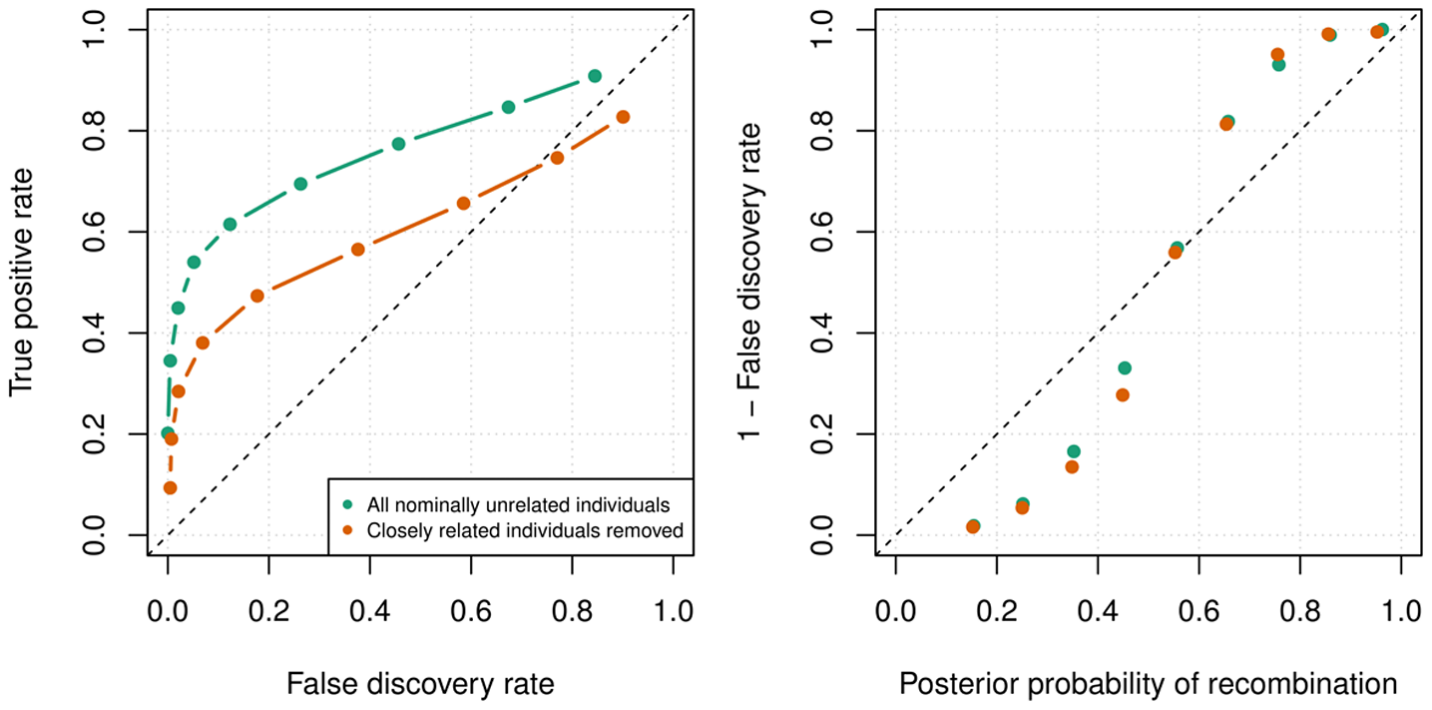
Recombination detection accuracy in uninformative duos simulated from chromosome X data in the Val Borbera cohort. The green values are for a cohort with nominally unrelated individuals and the orange values are for a cohort that has been filtered such that no individuals are closely related (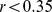). Left: The ROC curves for recombination detection in uninformative duos for our duo HMM using the SHAPEIT2 haplotypes. Right: The average number of correct detections against the average posterior probability. Setting a high probability threshold ensures a very low false discovery rate.

Figure 7 (left) shows ROC curves for detecting recombination events applied to our simulated uninformative meioses. We found that recombination events could be detected with low false discovery rates, but the power of the method to detect recombination events is clearly limited by the demography of the sample. When closely related individuals were not removed we see that 53.51% of events could be detected with a false discovery rate of 5%, but when closely related individuals were filtered we could only detect 34.10% of events with 5% false discovery rate (posterior probability threshold of 0.7). Importantly, the posterior probabilities of a recombination event appear roughly calibrated (Figure 7 right) so by setting a high probability threshold, researchers can be confident they are detecting true events with our method.

#### Using detected recombinations for association scans of hotspot usage

Figure S34 (left) shows the signal of association in the *PRDM9* region for a meta analysis of all the European cohorts (GPC excluded). When only the 618 informative parents were used (top) we found a minimum P-value of 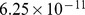 at SNP rs2162866. The addition of 466 individuals lead to a modest increase in signal (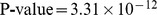) at the same SNP. Figure S34 (right) plots the 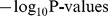 when all individuals are used against the 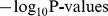 when only informative individuals are used, demonstrating a consistent increase in signal in the region. This suggests that our method is indeed detecting true recombination events.

## Discussion

Long range phasing has been a topic of interest since its inception by Kong (2008) [12] and has great potential for the analysis of genomic data, particularly as cohort sizes increase and hence more IBD sharing becomes present between individuals. Whilst the deCODE project has generated some excellent results, they have the advantage of an extremely powerful data set containing substantial amounts of IBD sharing which allows a rule based approach to long range phasing that yields very accurate haplotypes. This is not a luxury available to many research groups. We demonstrate that SHAPEIT2 implicitly performs this very accurate long range phasing when possible, whilst still leveraging LD when it is not.

Using eight cohorts from isolated and non-isolated populations, all containing explicitly related individuals, we have carried out a comprehensive evaluation of approaches for haplotype estimation in the presence of IBD sharing. We compared approaches that are specifically focused on estimation of haplotypes in isolated samples (SLRP) and others (SHAPEIT2, Beagle and HAPI-UR) that were designed predominantly for cohorts of nominally unrelated individuals. Our experiments show that the SHAPEIT2 method provides high quality haplotypes that are more accurate than those estimated by SLRP, whereas Beagle and HAPI-UR produce results that are worse than SLRP. We find that the SE rates of SHAPEIT2 are a fraction of a percent in all cohorts, whereas the approaches BEAGLE and HAPI-UR produce SEs that are an order of magnitude larger.

A big disadvantage of existing pedigree analysis software is the inability to leverage wider cohort information to resolve sites that are heterozygous throughout a particular pedigree. Hence there is a need for software that can fully leverage the relatedness within pedigrees for accurate phase whilst overcoming the limitations of traditional pedigree analysis software. We propose a two-stage approach in which SHAPEIT2 is first run ignoring *all* explicit family information. We then apply the duoHMM method to incorporate the pedigree information in a cohort to further increase the accuracy of haplotypes inferred. The duoHMM method infers the inheritance pattern in parent-child duos, detects genotyping errors and can correct switch errors.

We have found that the resulting haplotypes from our method are so accurate that we can infer recombination events in parent-child duos. We use the output of our duoHMM to estimate the probability that a recombination event occurs between each pair of heterozygous markers. When applied to all eight cohorts across whole chromosomes we find that the number of recombination events inferred by our method shows close agreement with the genetic length of each chromosome. We also find that the observed number of recombination events per individual closely matches what we expect to observe based on genetic map estimates. These results are also much better than those produced from Merlin, which shows elevated rates of recombination events across all chromosomes. On realistic simulated data our method (TPR = 92.4%, FDR = 3.78%) substantially outperforms Merlin (TPR = 90.57%, FDR = 62.48%).

An additional benefit of our method is that we can attempt to infer recombination events in trios and duos. Methods that explicitly phase trios and duos using the pedigree information cannot infer recombination events since they infer only the transmitted haplotypes of the parents. We evaluated this approach via simulation and found that we have 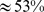 power to detect events at a 5% false discovery rate when the duo is phased in an isolated cohort that may contain close relatives. When close relatives are removed we have 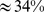 power to detect events at a 5% false discovery rate. Cohorts that contain explicit trios and duos could be phased using methods that explicitly use this information if desired although the ability to infer recombination events would be lost and parents would be estimated as a pair of transmitted and untransmitted haplotypes.

Using our method we are able to demonstrate that the recombination events that we infer from otherwise uninformative duos and trios can add power to association scans for recombination phenotypes. Specifically, at the established *PRDM9* locus we are able to show that including these extra recombination events increases the signal of association for a hot spot usage phenotype. The field of study of recombination continues to be very active [47]. Future studies will look at recombination in isolated populations in sub-saharan Africa. Our method will allow GWAS of recombination phenotypes to be carried out in these populations, extracting as much information as possible from the data.

Precisely determining why these large differences in performance between the methods exist is difficult. We suspect that the reason resides in the fact that within the SHAPEIT2 method the haplotypes of each individual are explicitly modelled as a mosaic of the underlying haplotypes of other individuals [48]. In other words the underlying haplotype sharing between two individuals can be explicitly captured by allowing each individual to ‘copy’ the haplotypes of another individual over a long stretch of sequence. BEAGLE takes a different approach by collapsing the haplotype information of the sample into a compact graph. Each individual's haplotypes are then updated within the method conditional upon this graph. Thus no direct comparison between pairs of individuals is made and thus the information regarding long stretches of shared sequence between individuals is lost. These comments also apply to HAPI-UR which uses a different graph to encode the haplotypes of the samples. Our results are consistent across a range of cohorts with differing levels of relatedness. Most of these cohorts are isolated cohorts but the Split and GPC cohorts contain levels of relatedness that might be expected in a GWAS cohort.

In this paper we have focused exclusively on genetic data from human samples but our methods may also be useful in the fields of animal and plant genetics where cohorts with high levels of relatedness are prevalent [49]. This method may also find utility in studies that aim to locate IBD segments between individuals. Based on these results we might suggest that a strategy of estimating haplotypes with SHAPEIT2 followed by application of the GERMLINE method [50] for IBD inference from haplotype data may provide an accurate and efficient solution. As cohorts increase in size, or as cohorts are combined, the chance of any individual sharing a close relative in the cohort increases. Methods such as SHAPEIT2, that can accurately leverage this IBD information when estimating haplotypes may help to extract the most information from such large cohorts.

Previous research has demonstrated SHAPEIT2's effectiveness for phasing cohorts of unrelated individuals, in this paper we demonstrate that SHAPEIT2 is in fact effective across the full spectrum of relatedness. This means that researchers with cohorts with any mixture of unrelated, distantly related or directly related individuals have a flexible tool available which can exploit all of these degrees of relatedness for very accurate haplotype estimates.

## Supporting Information

Figure S1Computation time in hours for phasing chromosome 10 for each of the *full* cohorts run as unrelated (second column of Table 4). All jobs were run on an Intel Xeon CPU E5-2690 (2.90 GHz) CPU with 256 GB of RAM. This is the total compute time for three runs of HAPI-UR as specified in the manual. Beagle was ran *without* the lowmem option for better performance. Whilst no parallelism was employed here, options exists for exploiting multiple processors with varying degrees of difficulty for each piece of software. SHAPEIT2 and SLRP have the ability to run on 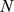 threads resulting in an approximately 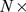 reduction in computation time. HAPI-UR 3× can be run as three simultaneous processes rather than sequentially. Most simply, the genome can be partitioned into chunks which are phased separately with the results being ligated.(TIFF)

Figure S2 The proportion of heterozygote sites phased by Merlin against the size of the pedigree (all cohorts). Note pedigrees of the same size may have different structures. For example some pedigrees of size three are a parent and two children (as opposed to a mother-father-child trio).
(TIFF)

Figure S3Computational performance of Merlin on simulated nuclear families of increasing size. Computation time (left) and memory usage (right) for Merlin's haplotyping routine applied to simulated nuclear families with increasing numbers of siblings assayed at 16,297 loci on chromosome 10 (Intel Xeon CPU E5-2690 2.90 GHz with 256 GB RAM). For pedigrees with 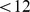 non-founders, Merlin's computation time is negligible but the method will clearly become intractable for larger pedigrees.(TIFF)

Figure S4 Schematic of phasing evaluation pipeline. This figure shows a toy example to illustrate the way in which we have used mixtures of pedigrees and unrelated samples to assess the performance of different methods. The figure shows two families of size 3 and 4 respectively and 2 unrelated samples. We used Merlin to phase the two families (blue), providing accurate haplotypes in the founders. We then ran each of the methods SHAPEIT2, Beagle and SLRP on the data from the founders and the unrelated samples (pink). The haplotypes estimated in the founder individuals was then compared to the Merlin phased haplotypes from these samples.
(TIFF)

Figure S5Haplotype correction example using the DuoHMM. The Duo HMM Viterbi paths for a three father-child duos from a nuclear family (ie. 3 siblings) from the FVG cohort on chromosome 10. The four possible IBD states (A, B, C, D) are shown using colours pale blue, dark blue, light red and dark red respectively (although states A and B do not occur in this example). The left panel shows the path prior to any corrections, and the right panel after a minimum recombinant correction is applied. The second and third sibling initially had a 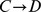 transition at around 25 mb, this is more likely a recombination event in the first child hence the parental haplotypes are switched after this point. The panel on the right has the corrected haplotypes, the number of recombination events required to explain the observed data has been reduced.(TIFF)

Figure S6 Evaluation of SHAPEIT2 and SLRP accuracy in IBD regions for the Val Borbera cohort. Left: The SHAPEIT2 switch error rates (within IBD regions) against the SLRP rates for each founder individual in the Val Borbera cohort. Both methods achieve low error rates but SHAPEIT2 has lower rates for most individuals. Right: The switch error rate for SHAPEIT2 *within* SLRP IBD regions against the rate *outside* SLRP IBD regions. Haplotypes are far more accurate when IBD sharing is present.
(TIFF)

Figure S7The duo HMM Viterbi paths for 50 mother-child duos from the CARL cohort on chromosome 10. The four possible IBD states (A, B, C, D) are shown using colours pale blue, dark blue, light red and dark red respectively. The left and right panels show the results of the duo HMM applied to the SHAPEIT2 and Beagle haplotypes respectively. Changes between a blue and red colour correspond to a 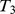 or 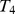 transition, both of which imply a switch error in the child. Changes of colour between light and dark blue or between light and dark red correspond to 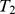 transitions, which correspond to a change on IBD state in the parent, and could be caused by a recombination or a switch error in the parent. The x-axis shows the sex-averaged genetic distance across the chromosome in centiMorgans.(TIFF)

Figure S8The duo HMM Viterbi paths for 50 father-child duos from the CARL cohort on chromosome 10. The four possible IBD states (A, B, C, D) are shown using colours pale blue, dark blue, light red and dark red respectively. The left and right panels show the results of the duo HMM applied to the SHAPEIT2 and Beagle haplotypes respectively. Changes between a blue and red colour correspond to a 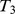 or 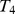 transition, both of which imply a switch error in the child. Changes of colour between light and dark blue or between light and dark red correspond to 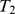 transitions, which correspond to a change on IBD state in the parent, and could be caused by a recombination or a switch error in the parent. The x-axis shows the sex-averaged genetic distance across the chromosome in centiMorgans.(TIFF)

Figure S9The duo HMM Viterbi paths for 50 mother-child duos from the FVG cohort on chromosome 10. The four possible IBD states (A, B, C, D) are shown using colours pale blue, dark blue, light red and dark red respectively. The left and right panels show the results of the duo HMM applied to the SHAPEIT2 and Beagle haplotypes respectively. Changes between a blue and red colour correspond to a 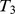 or 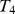 transition, both of which imply a switch error in the child. Changes of colour between light and dark blue or between light and dark red correspond to 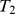 transitions, which correspond to a change on IBD state in the parent, and could be caused by a recombination or a switch error in the parent. The x-axis shows the sex-averaged genetic distance across the chromosome in centiMorgans.(TIFF)

Figure S10The duo HMM Viterbi paths for 50 father-child duos from the FVG cohort on chromosome 10. The four possible IBD states (A, B, C, D) are shown using colours pale blue, dark blue, light red and dark red respectively. The left and right panels show the results of the duo HMM applied to the SHAPEIT2 and Beagle haplotypes respectively. Changes between a blue and red colour correspond to a 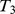 or 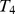 transition, both of which imply a switch error in the child. Changes of colour between light and dark blue or between light and dark red correspond to 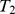 transitions, which correspond to a change on IBD state in the parent, and could be caused by a recombination or a switch error in the parent. The x-axis shows the sex-averaged genetic distance across the chromosome in centiMorgans.(TIFF)

Figure S11The duo HMM Viterbi paths for 50 mother-child duos from the CROATIA-Korcula cohort on chromosome 10. The four possible IBD states (A, B, C, D) are shown using colours pale blue, dark blue, light red and dark red respectively. The left and right panels show the results of the duo HMM applied to the SHAPEIT2 and Beagle haplotypes respectively. Changes between a blue and red colour correspond to a 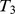 or 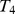 transition, both of which imply a switch error in the child. Changes of colour between light and dark blue or between light and dark red correspond to 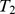 transitions, which correspond to a change on IBD state in the parent, and could be caused by a recombination or a switch error in the parent. The x-axis shows the sex-averaged genetic distance across the chromosome in centiMorgans.(TIFF)

Figure S12The duo HMM Viterbi paths for 50 father-child duos from the CROATIA-Korcula cohort on chromosome 10. The four possible IBD states (A, B, C, D) are shown using colours pale blue, dark blue, light red and dark red respectively. The left and right panels show the results of the duo HMM applied to the SHAPEIT2 and Beagle haplotypes respectively. Changes between a blue and red colour correspond to a 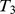 or 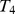 transition, both of which imply a switch error in the child. Changes of colour between light and dark blue or between light and dark red correspond to 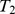 transitions, which correspond to a change on IBD state in the parent, and could be caused by a recombination or a switch error in the parent. The x-axis shows the sex-averaged genetic distance across the chromosome in centiMorgans.(TIFF)

Figure S13The duo HMM Viterbi paths for 50 mother-child duos from the ORCADES cohort on chromosome 10. The four possible IBD states (A, B, C, D) are shown using colours pale blue, dark blue, light red and dark red respectively. The left and right panels show the results of the duo HMM applied to the SHAPEIT2 and Beagle haplotypes respectively. Changes between a blue and red colour correspond to a 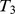 or 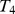 transition, both of which imply a switch error in the child. Changes of colour between light and dark blue or between light and dark red correspond to 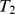 transitions, which correspond to a change on IBD state in the parent, and could be caused by a recombination or a switch error in the parent. The x-axis shows the sex-averaged genetic distance across the chromosome in centiMorgans.(TIFF)

Figure S14The duo HMM Viterbi paths for 50 father-child duos from the ORCADES cohort on chromosome 10. The four possible IBD states (A, B, C, D) are shown using colours pale blue, dark blue, light red and dark red respectively. The left and right panels show the results of the duo HMM applied to the SHAPEIT2 and Beagle haplotypes respectively. Changes between a blue and red colour correspond to a 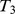 or 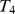 transition, both of which imply a switch error in the child. Changes of colour between light and dark blue or between light and dark red correspond to 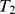 transitions, which correspond to a change on IBD state in the parent, and could be caused by a recombination or a switch error in the parent. The x-axis shows the sex-averaged genetic distance across the chromosome in centiMorgans.(TIFF)

Figure S15The duo HMM Viterbi paths for 50 mother-child duos from the CROATIA-Split cohort on chromosome 10. The four possible IBD states (A, B, C, D) are shown using colours pale blue, dark blue, light red and dark red respectively. The left and right panels show the results of the duo HMM applied to the SHAPEIT2 and Beagle haplotypes respectively. Changes between a blue and red colour correspond to a 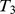 or 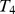 transition, both of which imply a switch error in the child. Changes of colour between light and dark blue or between light and dark red correspond to 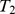 transitions, which correspond to a change on IBD state in the parent, and could be caused by a recombination or a switch error in the parent. The x-axis shows the sex-averaged genetic distance across the chromosome in centiMorgans.(TIFF)

Figure S16The duo HMM Viterbi paths for 50 father-child duos from the CROATIA-Split cohort on chromosome 10. The four possible IBD states (A, B, C, D) are shown using colours pale blue, dark blue, light red and dark red respectively. The left and right panels show the results of the duo HMM applied to the SHAPEIT2 and Beagle haplotypes respectively. Changes between a blue and red colour correspond to a 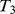 or 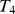 transition, both of which imply a switch error in the child. Changes of colour between light and dark blue or between light and dark red correspond to 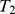 transitions, which correspond to a change on IBD state in the parent, and could be caused by a recombination or a switch error in the parent. The x-axis shows the sex-averaged genetic distance across the chromosome in centiMorgans.(TIFF)

Figure S17The duo HMM Viterbi paths for 50 mother-child duos from the Val Borbera cohort on chromosome 10. The four possible IBD states (A, B, C, D) are shown using colours pale blue, dark blue, light red and dark red respectively. The left and right panels show the results of the duo HMM applied to the SHAPEIT2 and Beagle haplotypes respectively. Changes between a blue and red colour correspond to a 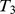 or 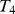 transition, both of which imply a switch error in the child. Changes of colour between light and dark blue or between light and dark red correspond to 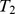 transitions, which correspond to a change on IBD state in the parent, and could be caused by a recombination or a switch error in the parent. The x-axis shows the sex-averaged genetic distance across the chromosome in centiMorgans.(TIFF)

Figure S18The duo HMM Viterbi paths for 50 father-child duos from the Val Borbera cohort on chromosome 10. The four possible IBD states (A, B, C, D) are shown using colours pale blue, dark blue, light red and dark red respectively. The left and right panels show the results of the duo HMM applied to the SHAPEIT2 and Beagle haplotypes respectively. Changes between a blue and red colour correspond to a 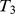 or 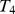 transition, both of which imply a switch error in the child. Changes of colour between light and dark blue or between light and dark red correspond to 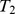 transitions, which correspond to a change on IBD state in the parent, and could be caused by a recombination or a switch error in the parent. The x-axis shows the sex-averaged genetic distance across the chromosome in centiMorgans.(TIFF)

Figure S19The duo HMM Viterbi paths for 50 mother-child duos from the CROATIA-Vis cohort on chromosome 10. The four possible IBD states (A, B, C, D) are shown using colours pale blue, dark blue, light red and dark red respectively. The left and right panels show the results of the duo HMM applied to the SHAPEIT2 and Beagle haplotypes respectively. Changes between a blue and red colour correspond to a 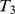 or 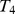 transition, both of which imply a switch error in the child. Changes of colour between light and dark blue or between light and dark red correspond to 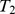 transitions, which correspond to a change on IBD state in the parent, and could be caused by a recombination or a switch error in the parent. The x-axis shows the sex-averaged genetic distance across the chromosome in centiMorgans.(TIFF)

Figure S20The duo HMM Viterbi paths for 50 father-child duos from the CROATIA-Vis cohort on chromosome 10. The four possible IBD states (A, B, C, D) are shown using colours pale blue, dark blue, light red and dark red respectively. The left and right panels show the results of the duo HMM applied to the SHAPEIT2 and Beagle haplotypes respectively. Changes between a blue and red colour correspond to a 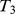 or 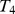 transition, both of which imply a switch error in the child. Changes of colour between light and dark blue or between light and dark red correspond to 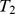 transitions, which correspond to a change on IBD state in the parent, and could be caused by a recombination or a switch error in the parent. The x-axis shows the sex-averaged genetic distance across the chromosome in centiMorgans.(TIFF)

Figure S21The duo HMM Viterbi paths for 50 mother-child duos from the GPC cohort on chromosome 10. The four possible IBD states (A, B, C, D) are shown using colours pale blue, dark blue, light red and dark red respectively. The left and right panels show the results of the duo HMM applied to the SHAPEIT2 and Beagle haplotypes respectively. Changes between a blue and red colour correspond to a 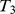 or 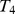 transition, both of which imply a switch error in the child. Changes of colour between light and dark blue or between light and dark red correspond to 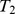 transitions, which correspond to a change on IBD state in the parent, and could be caused by a recombination or a switch error in the parent. The x-axis shows the sex-averaged genetic distance across the chromosome in centiMorgans.(TIFF)

Figure S22The duo HMM Viterbi paths for 50 father-child duos from the GPC cohort on chromosome 10. The four possible IBD states (A, B, C, D) are shown using colours pale blue, dark blue, light red and dark red respectively. The left and right panels show the results of the duo HMM applied to the SHAPEIT2 and Beagle haplotypes respectively. Changes between a blue and red colour correspond to a 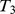 or 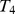 transition, both of which imply a switch error in the child. Changes of colour between light and dark blue or between light and dark red correspond to 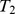 transitions, which correspond to a change on IBD state in the parent, and could be caused by a recombination or a switch error in the parent. The x-axis shows the sex-averaged genetic distance across the chromosome in centiMorgans.(TIFF)

Figure S23Detection of recombination in simulated extended pedigrees and comparison of SHAPEIT2 and Merlin haplotype accuracy for realistic data (top) and ideal data (bottom). Left: True positive rate (TPR) versus false discovery rate (FDR) for recombination detection using SHAPEIT2+duoHMM (red) versus Merlin (blue). Merlin detects 90.57% of crossovers with a substantial FDR of 62.48% whilst SHAPEIT2+duoHMM detects 92.40% of events with an FDR of 3.78% (the red point at 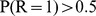). On ideal data SHAPEIT2+duoHMM achieve a 95.75% TPR and 0.71% FDR and Merlin has 95.66% and 3.48% respectively. Right: Switch error for SHAPEIT2 (DuoHMM corrected) versus Merlin. The black point is the mean for each method. Merlin had 0.215% (0.021% ideal scenario) switch error while SHAPEIT2 had a rate of 0.033% (0.005% ideal scenario).(TIFF)

Figure S24 Switch error results on simulated data for extended pedigrees. Points are coloured according to what family information was used by Beagle in duo/trio mode; red meaning an individual could not be included in a duo or trio. Left: Beagle duo/trio switch error rate (0.269% average SE) against switch error rate for SHAPEIT2 when all individuals are treated as unrelated (0.104% average SE). Centre: After applying the duoHMM haplotype corrections to SHAPEIT2 (0.065% average SE). Right: Switch error after masking genotypes flagged as erroneous by the SHAPEIT2+duoHMM, Beagle duo/trio phasing is reduced to 0.231% and SHAPEIT2+duoHMM to 0.034%.
(TIFF)

Figure S25 Genome-wide number of recombinations found per individual for SHAPEIT versus Merlin for all informative meioses in real data sets. The number of recombinations found by SHAPEIT2 against Merlin for each of 661 meioses (all informative duos from all cohorts). Maternal are in green and paternal purple. Triangles represent a truncated value. While there is correlation between the two methods, Merlin more frequently finds an implausible number of recombinations.
(TIFF)

Figure S26 Carlantino cohort recombination distributions. Q-Q plots for the observed against expected number of recombinations estimated by each method for paternal meioses (left) and maternal meioses (right), only duos that were part of an informative pedigree were used. For the expected distribution of recombination rates, a Poisson distribution using the genetic lengths from the 2002 deCODE Map was used (with rate parameter 42.81 and 25.9 for maternal and paternal recombinations respectively.)
(TIFF)

Figure S27 FVG cohort - recombination distributions. Q-Q plots for the observed against expected number of recombinations estimated by each method for paternal meioses (left) and maternal meioses (right), only duos that were part of an informative pedigree were used. For the expected distribution of recombination rates, a Poisson distribution using the genetic lengths from the 2002 deCODE Map was used (with rate parameter 42.81 and 25.9 for maternal and paternal recombinations respectively.)
(TIFF)

Figure S28 Korcula cohort - recombination distributions. Q-Q plots for the observed against expected number of recombinations estimated by each method for paternal meioses (left) and maternal meioses (right), only duos that were part of an informative pedigree were used. For the expected distribution of recombination rates, a Poisson distribution using the genetic lengths from the 2002 deCODE Map was used (with rate parameter 42.81 and 25.9 for maternal and paternal recombinations respectively.)
(TIFF)

Figure S29 ORCADES cohort - recombination distributions. Q-Q plots for the observed against expected number of recombinations estimated by each method for paternal meioses (left) and maternal meioses (right), only duos that were part of an informative pedigree were used. For the expected distribution of recombination rates, a Poisson distribution using the genetic lengths from the 2002 deCODE Map was used (with rate parameter 42.81 and 25.9 for maternal and paternal recombinations respectively.)
(TIFF)

Figure S30 Valborbera cohort - recombination distributions. Q-Q plots for the observed against expected number of recombinations estimated by each method for paternal meioses (left) and maternal meioses (right), only duos that were part of an informative pedigree were used. For the expected distribution of recombination rates, a Poisson distribution using the genetic lengths from the 2002 deCODE Map was used (with rate parameter 42.81 and 25.9 for maternal and paternal recombinations respectively.)
(TIFF)

Figure S31 Vis cohort - recombination distributions. Q-Q plots for the observed against expected number of recombinations estimated by each method for paternal meioses (left) and maternal meioses (right), only duos that were part of an informative pedigree were used. For the expected distribution of recombination rates, a Poisson distribution using the genetic lengths from the 2002 deCODE Map was used (with rate parameter 42.81 and 25.9 for maternal and paternal recombinations respectively.)
(TIFF)

Figure S32 GPC cohort - recombination distributions. Q-Q plots for the observed against expected number of recombinations estimated by each method for paternal meioses (left) and maternal meioses (right), only duos that were part of an informative pedigree were used. For the expected distribution of recombination rates, a Poisson distribution using the genetic lengths from the 2002 deCODE Map was used (with rate parameter 42.81 and 25.9 for maternal and paternal recombinations respectively.)
(TIFF)

Figure S33 Recombination rates in regions around crossover detections by each method. Average recombination rate (from the HapMap LD map) centred on the location (the average of the two flanking heterozygous positions) of 34344 crossovers found by both SHAPEIT2 and Merlin (black), 4127 crossovers found only by SHAPEIT2 (red) and 32580 crossovers found only by Merlin (blue). Events found only by Merlin are in regions with less recombination on average than those found by by SHAPEIT2 suggesting a higher false detection rate.
(TIFF)

Figure S34Association testing between *PRDM9* region and hotspot usage phenotype for European cohorts. Left: The 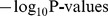 for the European meta-analysis for association between PRDM9 variants and the ‘hotspot usage’ phenotype. We used 618 informative and 466 uninformative parents in this analysis. Right: The 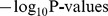 of this analysis plotted against the 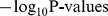 when only the 618 informative parents are used. The additional samples yield a modest increase in power.(TIFF)

Table S1 Frequencies of different pedigree sizes within each of the cohorts. Pedigrees of size 1 are individuals not part of an explicit pedigree. “Unrelated” is the sum of the number of pedigree founders and the number of individuals not in any pedigree. Note due to unspecified relationships, some of these individuals may still be closely related.
(PDF)

Table S2 The genotype confusion matrix used to simulate genotyping errors in our simulation studies. This is based on the discordance between Illumina Omni2.5S and Affymetrix Axiom chips on 1000 Genomes individuals. We took the Axiom genotypes as “truth” but halved the discordance and normalised the diagonal appropriately (the missing rate was left unchanged). This is to account for discordance that was actually due to Axiom chip errors.
(PDF)

Table S3 The average number of corrections applied to haplotypes for each method. ‘P’ and ‘M’ denotes when we correct a child's haplotypes using information from the parental (P) or maternal (M) haplotypes, to ensure consistent gene flow. ‘C’ denotes when multiple children were used to find the minimum recombinant parental haplotypes. Very few corrections are required for the SHAPEIT2 haplotypes compared to Beagle and HAPI-UR, this is also evident from the switch error improvements shown in Table 2. Cohort abbreviations: CARL - Carlantino, FVG - Friuli Venezia Giulia, GPC - Ugandan General Population Cohort, KOR - CROATIA-Korcula, ORC - Orkney Complex Disease Study, SPL - CROATIA-Split, VB - Val Borbera. VIS - CROATIA-Vis.
(PDF)
