# Supplementary figures and images for: A General Approach for Haplotype Phasing across the Full Spectrum of Relatedness
Source: PLoS Genet. 2014 Apr 17;10(4):e1004234. doi: 10.1371/journal.pgen.1004234 (PMC3990520; doi:10.1371/journal.pgen.1004234)

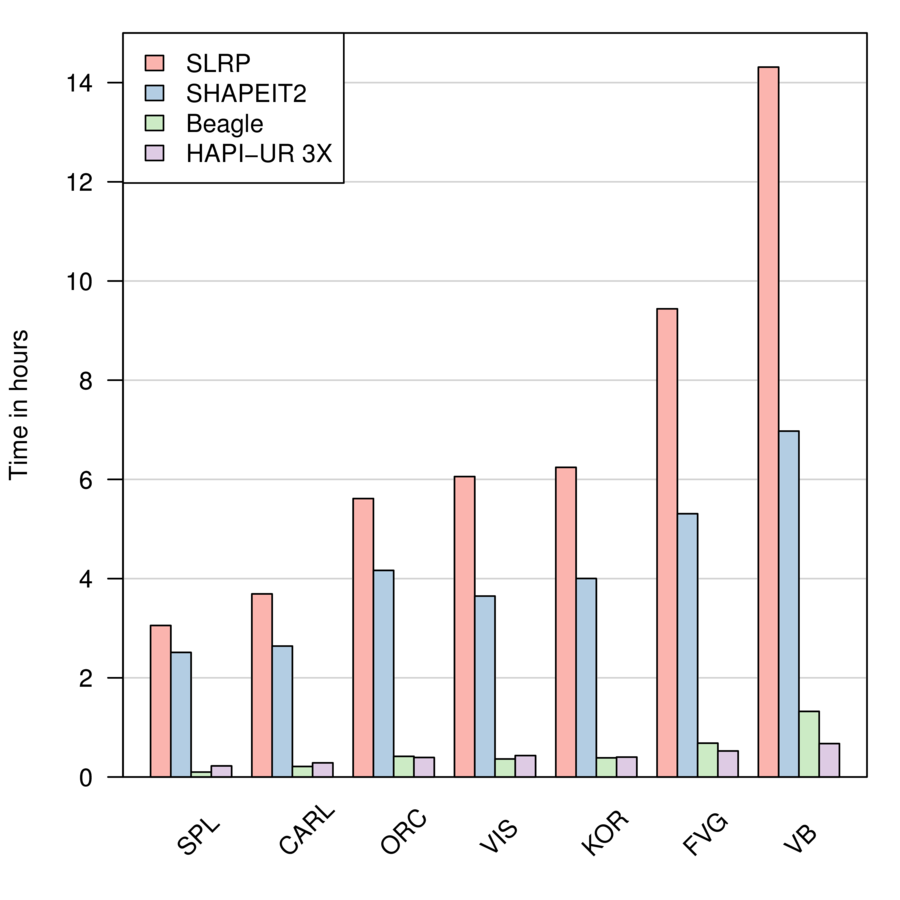

Supplement: Figure S1 — Computation time in hours for phasing chromosome 10 for each of the full cohorts run as unrelated (second column of Table 4). All jobs were run on an Intel Xeon CPU E5-2690 (2.90 GHz) CPU with 256 GB of RAM. This is the total compute time for three runs of HAPI-UR as specified in the manual. Beagle was ran without the lowmem option for better performance. Whilst no parallelism was employed here, options exists for exploiting multiple processors with varying degrees of difficulty for each piece of software. SHAPEIT2 and SLRP have the ability to run on threads resulting in an approximately reduction in computation time. HAPI-UR 3× can be run as three simultaneous processes rather than sequentially. Most simply, the genome can be partitioned into chunks which are phased separately with the results being ligated. (TIFF) [file pgen.1004234.s001.tif]

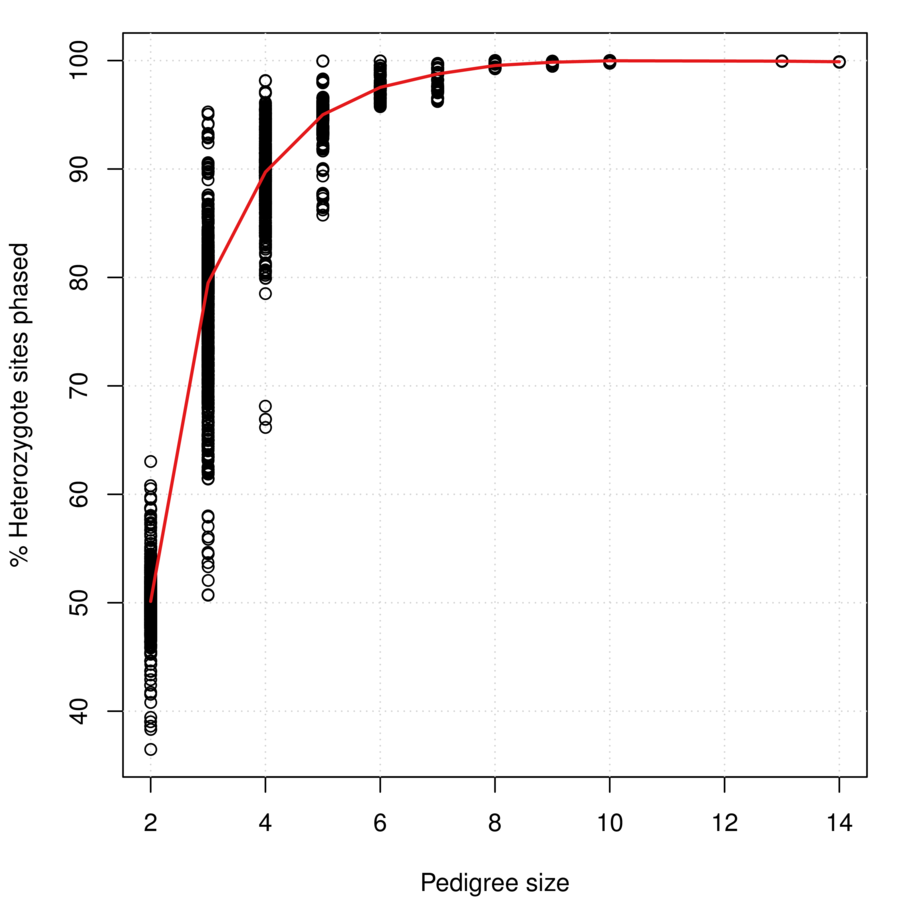

Supplement: Figure S2 — The proportion of heterozygote sites phased by Merlin against the size of the pedigree (all cohorts). Note pedigrees of the same size may have different structures. For example some pedigrees of size three are a parent and two children (as opposed to a mother-father-child trio). (TIFF) [file pgen.1004234.s002.tif]

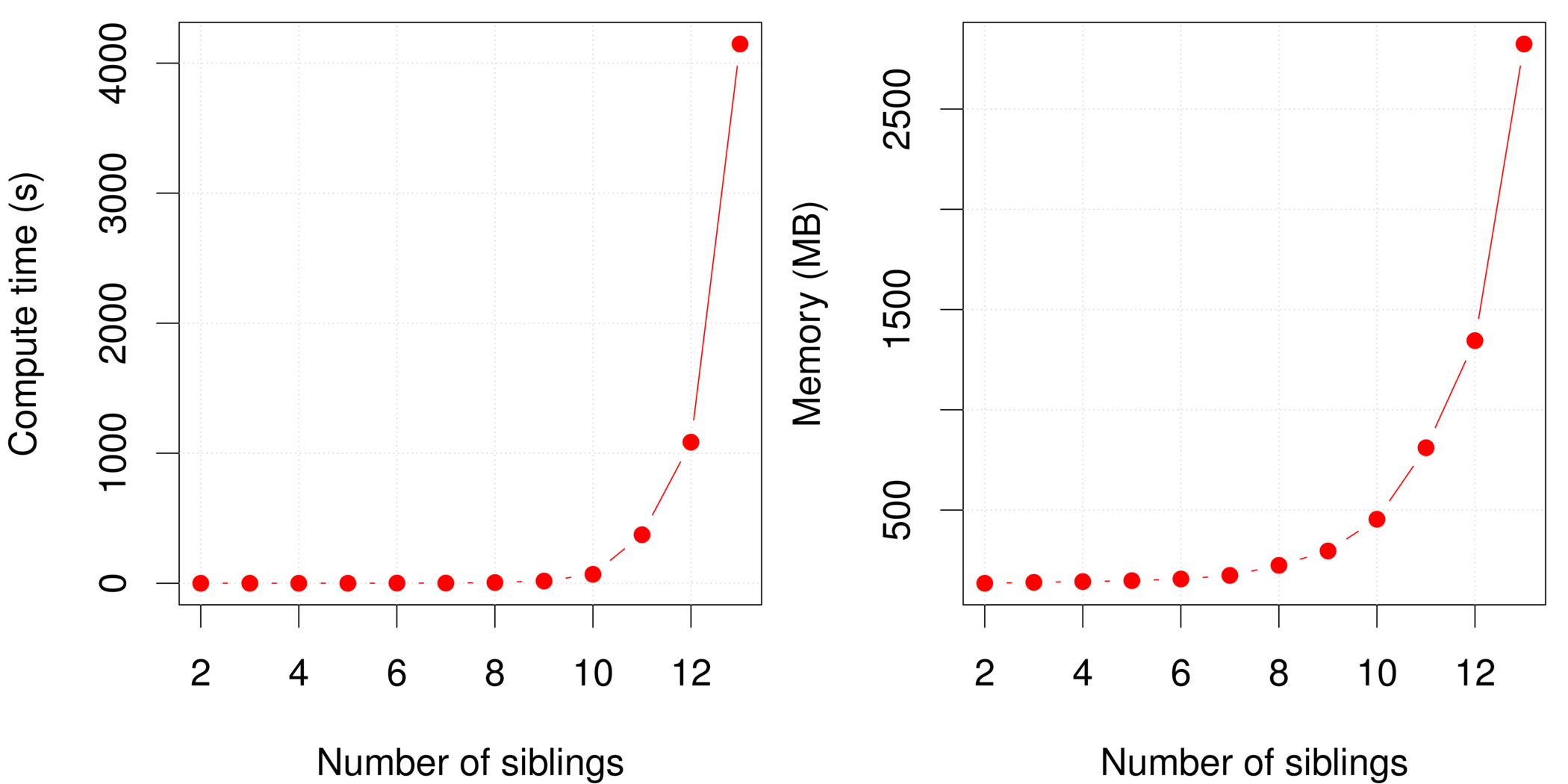

Supplement: Figure S3 — Computational performance of Merlin on simulated nuclear families of increasing size. Computation time (left) and memory usage (right) for Merlin's haplotyping routine applied to simulated nuclear families with increasing numbers of siblings assayed at 16,297 loci on chromosome 10 (Intel Xeon CPU E5-2690 2.90 GHz with 256 GB RAM). For pedigrees with non-founders, Merlin's computation time is negligible but the method will clearly become intractable for larger pedigrees. (TIFF) [file pgen.1004234.s003.tif]

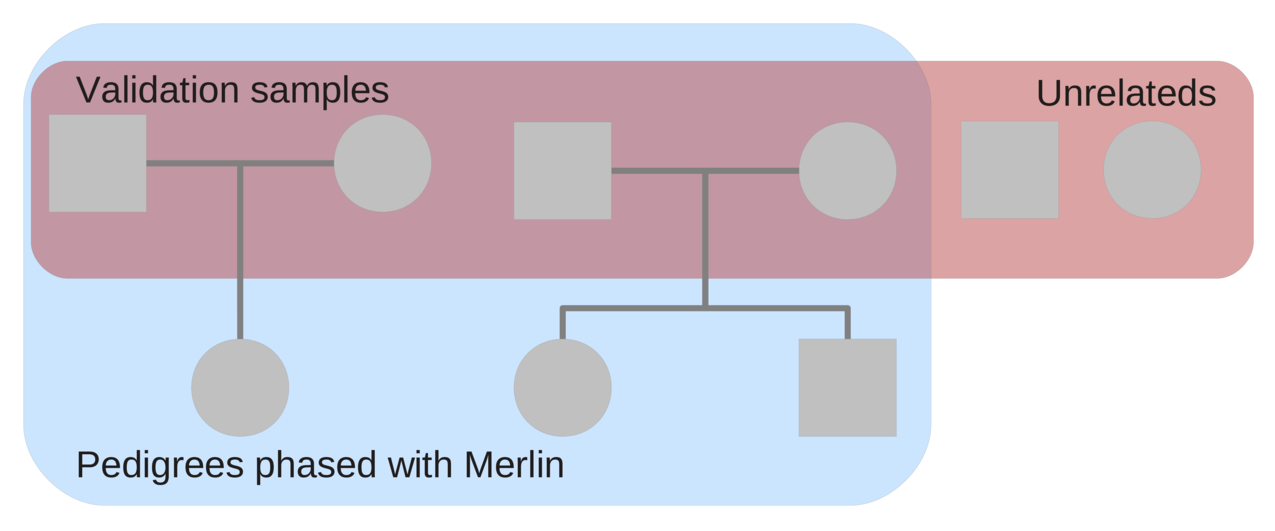

Supplement: Figure S4 — Schematic of phasing evaluation pipeline. This figure shows a toy example to illustrate the way in which we have used mixtures of pedigrees and unrelated samples to assess the performance of different methods. The figure shows two families of size 3 and 4 respectively and 2 unrelated samples. We used Merlin to phase the two families (blue), providing accurate haplotypes in the founders. We then ran each of the methods SHAPEIT2, Beagle and SLRP on the data from the founders and the unrelated samples (pink). The haplotypes estimated in the founder individuals was then compared to the Merlin phased haplotypes from these samples. (TIFF) [file pgen.1004234.s004.tif]

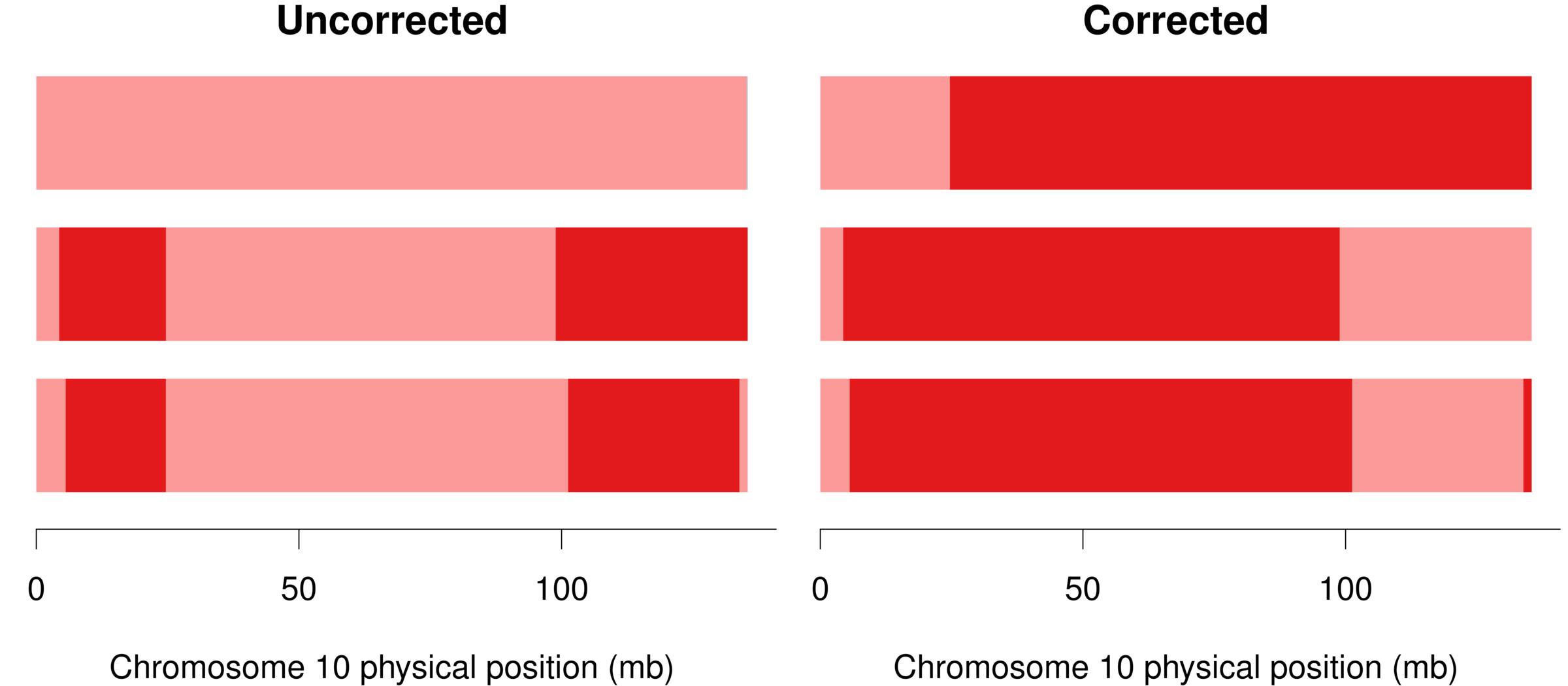

Supplement: Figure S5 — Haplotype correction example using the DuoHMM. The Duo HMM Viterbi paths for a three father-child duos from a nuclear family (ie. 3 siblings) from the FVG cohort on chromosome 10. The four possible IBD states (A, B, C, D) are shown using colours pale blue, dark blue, light red and dark red respectively (although states A and B do not occur in this example). The left panel shows the path prior to any corrections, and the right panel after a minimum recombinant correction is applied. The second and third sibling initially had a transition at around 25 mb, this is more likely a recombination event in the first child hence the parental haplotypes are switched after this point. The panel on the right has the corrected haplotypes, the number of recombination events required to explain the observed data has been reduced. (TIFF) [file pgen.1004234.s005.tif]

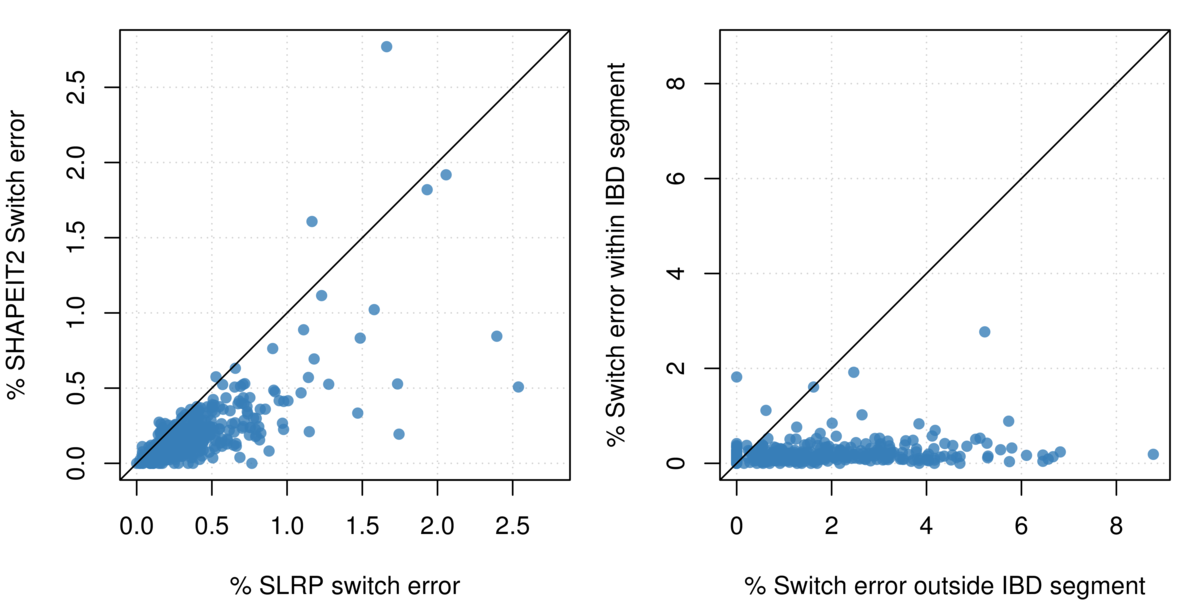

Supplement: Figure S6 — Evaluation of SHAPEIT2 and SLRP accuracy in IBD regions for the Val Borbera cohort. Left: The SHAPEIT2 switch error rates (within IBD regions) against the SLRP rates for each founder individual in the Val Borbera cohort. Both methods achieve low error rates but SHAPEIT2 has lower rates for most individuals. Right: The switch error rate for SHAPEIT2 within SLRP IBD regions against the rate outside SLRP IBD regions. Haplotypes are far more accurate when IBD sharing is present. (TIFF) [file pgen.1004234.s006.tif]

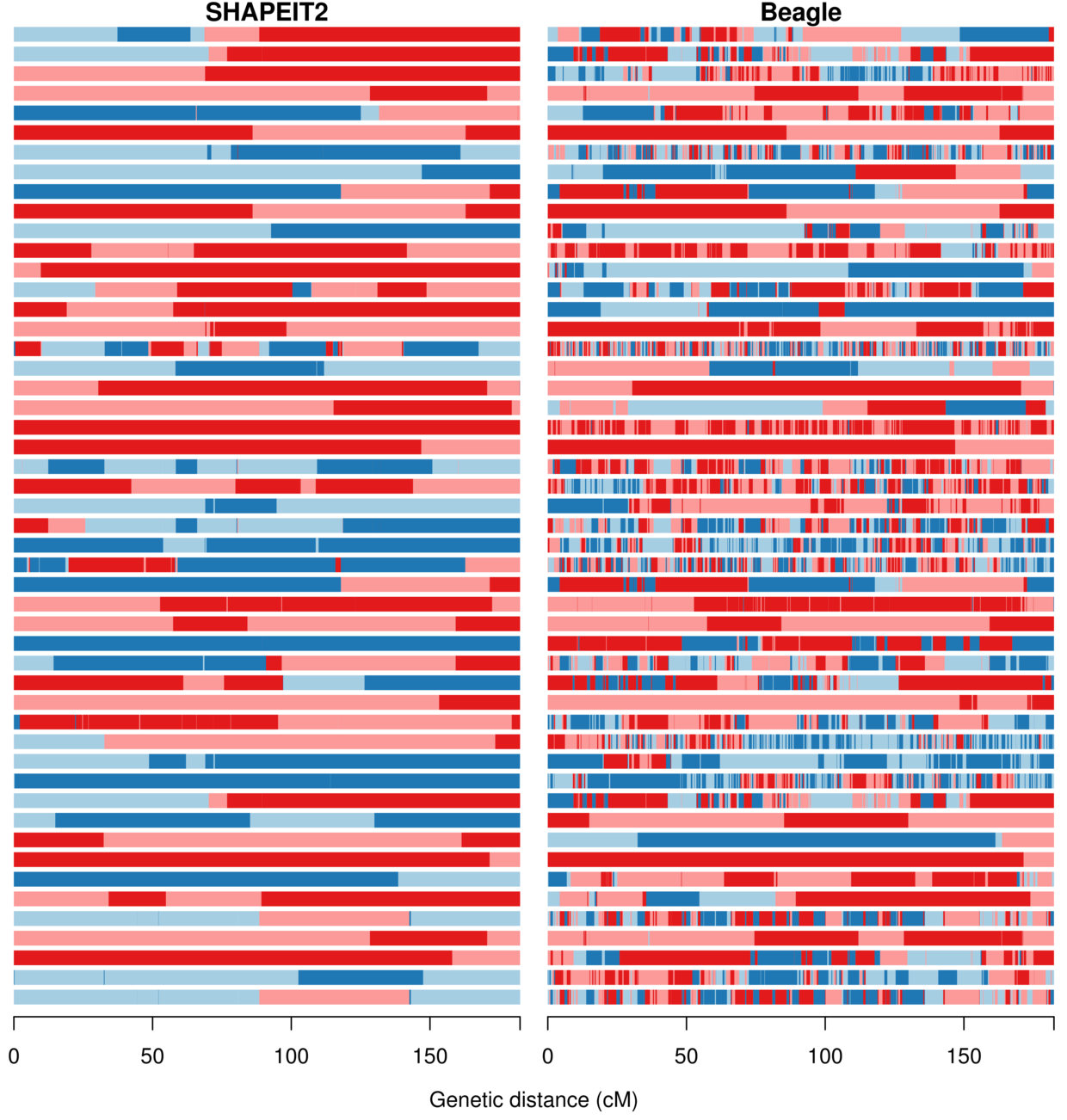

Supplement: Figure S7 — The duo HMM Viterbi paths for 50 mother-child duos from the CARL cohort on chromosome 10. The four possible IBD states (A, B, C, D) are shown using colours pale blue, dark blue, light red and dark red respectively. The left and right panels show the results of the duo HMM applied to the SHAPEIT2 and Beagle haplotypes respectively. Changes between a blue and red colour correspond to a or transition, both of which imply a switch error in the child. Changes of colour between light and dark blue or between light and dark red correspond to transitions, which correspond to a change on IBD state in the parent, and could be caused by a recombination or a switch error in the parent. The x-axis shows the sex-averaged genetic distance across the chromosome in centiMorgans. (TIFF) [file pgen.1004234.s007.tif]

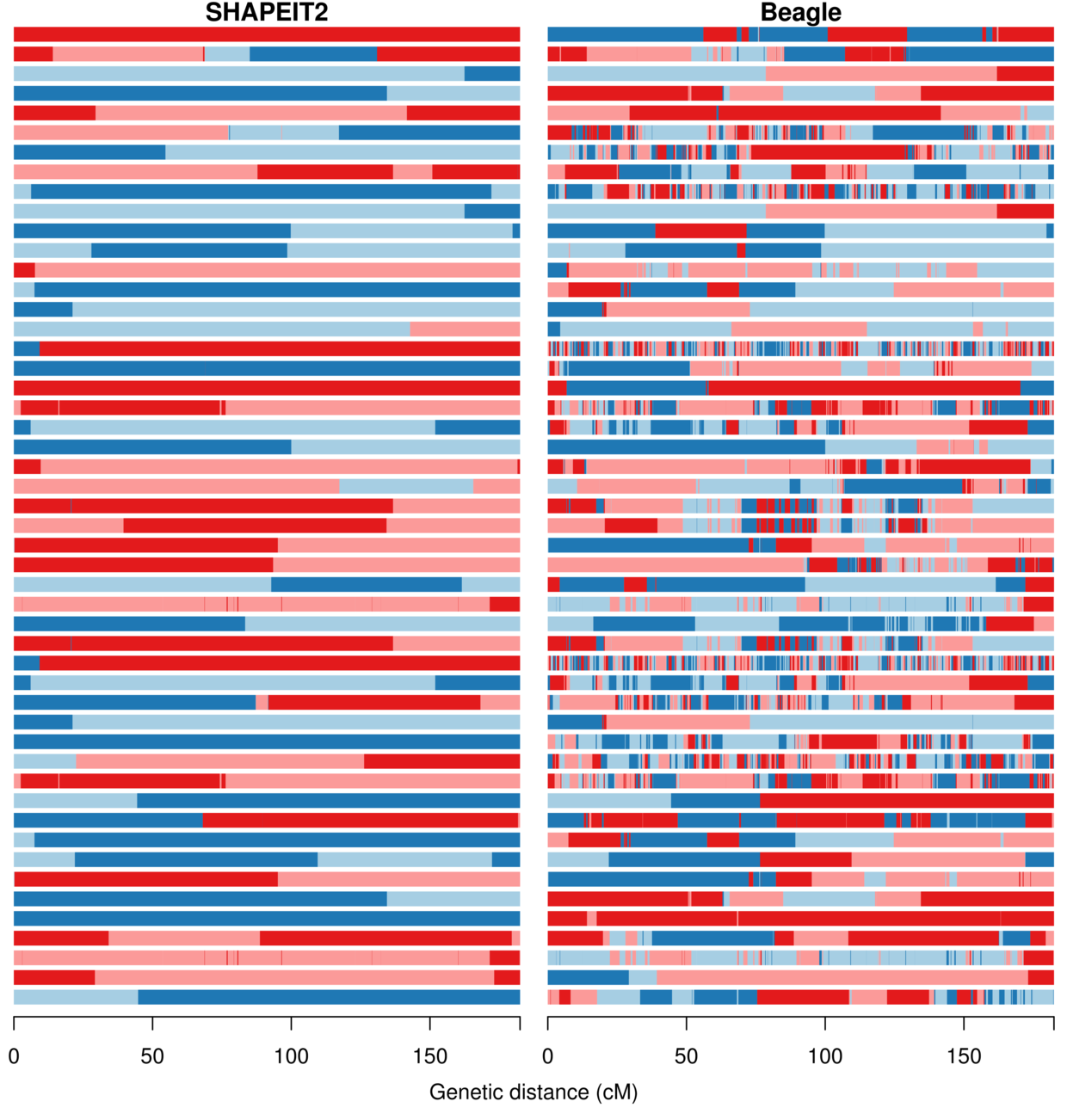

Supplement: Figure S8 — The duo HMM Viterbi paths for 50 father-child duos from the CARL cohort on chromosome 10. The four possible IBD states (A, B, C, D) are shown using colours pale blue, dark blue, light red and dark red respectively. The left and right panels show the results of the duo HMM applied to the SHAPEIT2 and Beagle haplotypes respectively. Changes between a blue and red colour correspond to a or transition, both of which imply a switch error in the child. Changes of colour between light and dark blue or between light and dark red correspond to transitions, which correspond to a change on IBD state in the parent, and could be caused by a recombination or a switch error in the parent. The x-axis shows the sex-averaged genetic distance across the chromosome in centiMorgans. (TIFF) [file pgen.1004234.s008.tif]

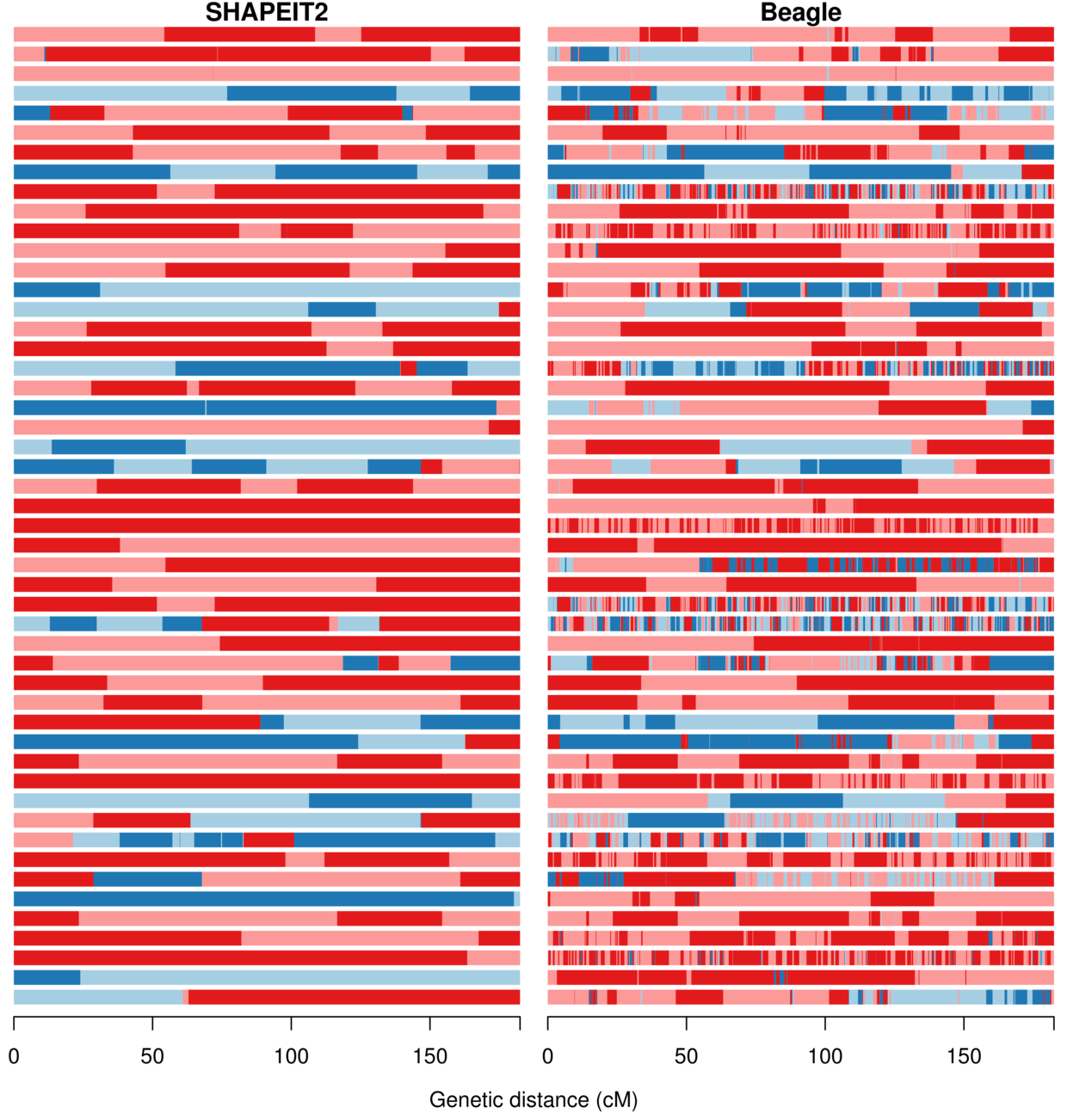

Supplement: Figure S9 — The duo HMM Viterbi paths for 50 mother-child duos from the FVG cohort on chromosome 10. The four possible IBD states (A, B, C, D) are shown using colours pale blue, dark blue, light red and dark red respectively. The left and right panels show the results of the duo HMM applied to the SHAPEIT2 and Beagle haplotypes respectively. Changes between a blue and red colour correspond to a or transition, both of which imply a switch error in the child. Changes of colour between light and dark blue or between light and dark red correspond to transitions, which correspond to a change on IBD state in the parent, and could be caused by a recombination or a switch error in the parent. The x-axis shows the sex-averaged genetic distance across the chromosome in centiMorgans. (TIFF) [file pgen.1004234.s009.tif]

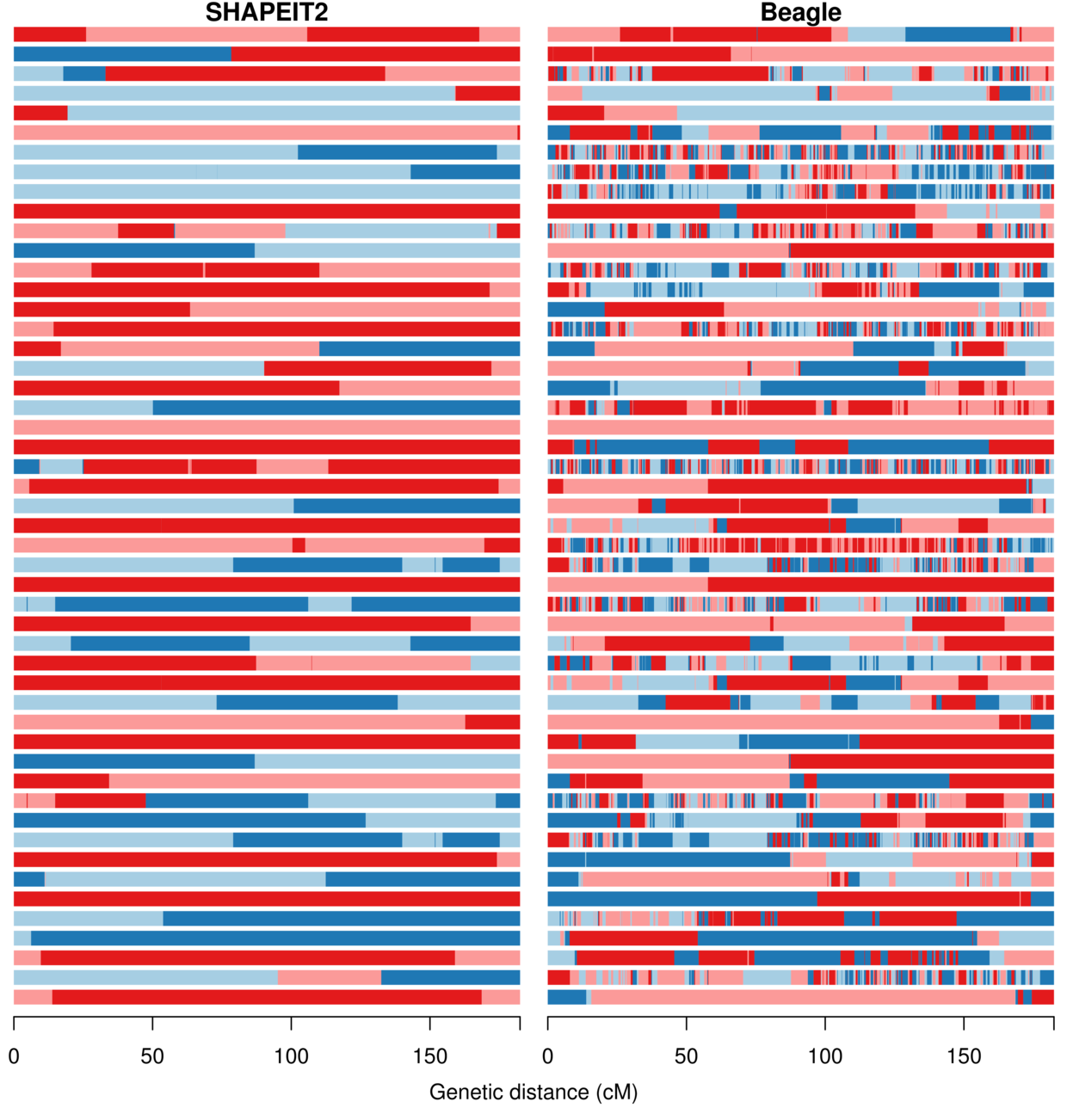

Supplement: Figure S10 — The duo HMM Viterbi paths for 50 father-child duos from the FVG cohort on chromosome 10. The four possible IBD states (A, B, C, D) are shown using colours pale blue, dark blue, light red and dark red respectively. The left and right panels show the results of the duo HMM applied to the SHAPEIT2 and Beagle haplotypes respectively. Changes between a blue and red colour correspond to a or transition, both of which imply a switch error in the child. Changes of colour between light and dark blue or between light and dark red correspond to transitions, which correspond to a change on IBD state in the parent, and could be caused by a recombination or a switch error in the parent. The x-axis shows the sex-averaged genetic distance across the chromosome in centiMorgans. (TIFF) [file pgen.1004234.s010.tif]

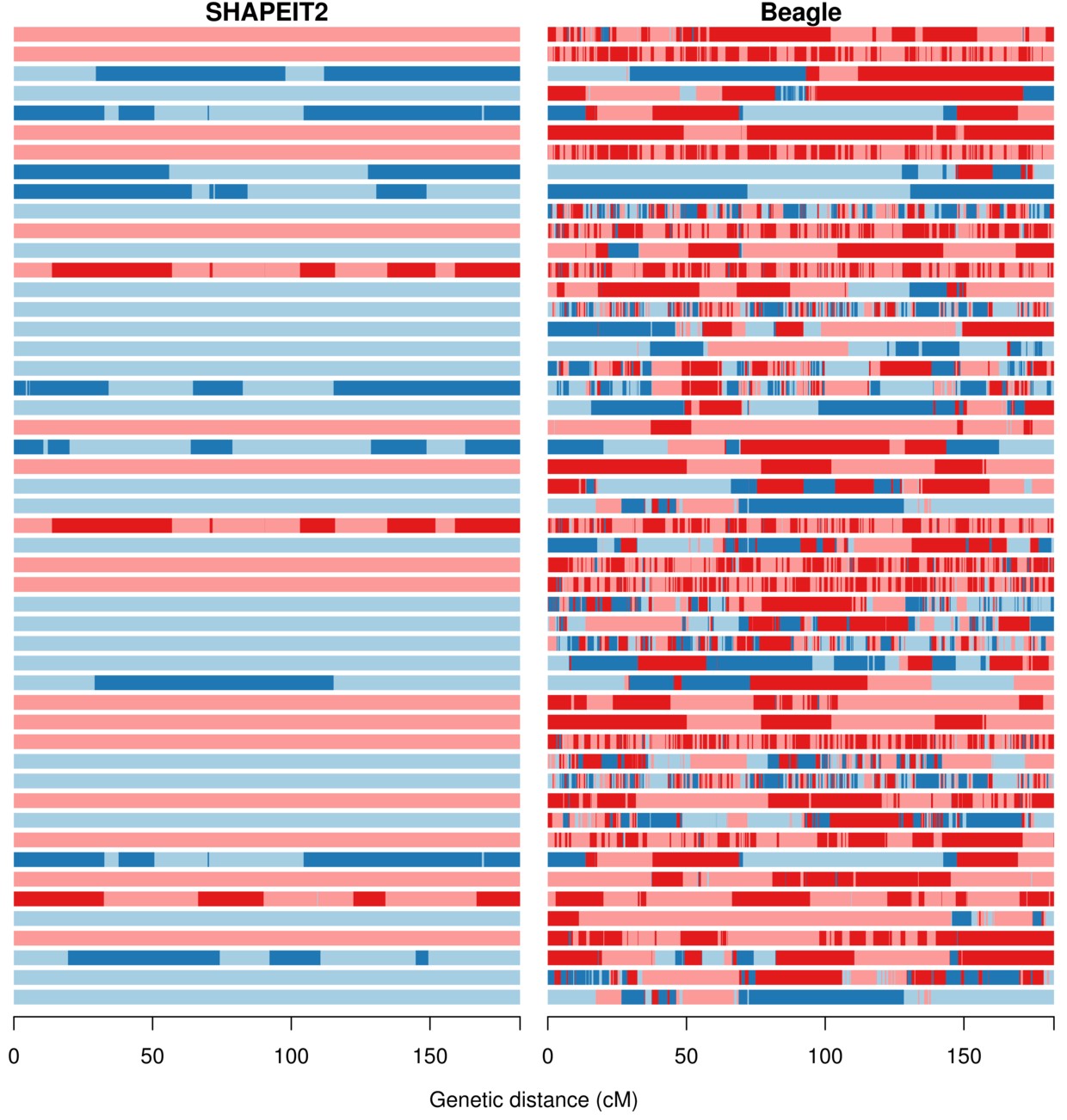

Supplement: Figure S11 — The duo HMM Viterbi paths for 50 mother-child duos from the CROATIA-Korcula cohort on chromosome 10. The four possible IBD states (A, B, C, D) are shown using colours pale blue, dark blue, light red and dark red respectively. The left and right panels show the results of the duo HMM applied to the SHAPEIT2 and Beagle haplotypes respectively. Changes between a blue and red colour correspond to a or transition, both of which imply a switch error in the child. Changes of colour between light and dark blue or between light and dark red correspond to transitions, which correspond to a change on IBD state in the parent, and could be caused by a recombination or a switch error in the parent. The x-axis shows the sex-averaged genetic distance across the chromosome in centiMorgans. (TIFF) [file pgen.1004234.s011.tif]

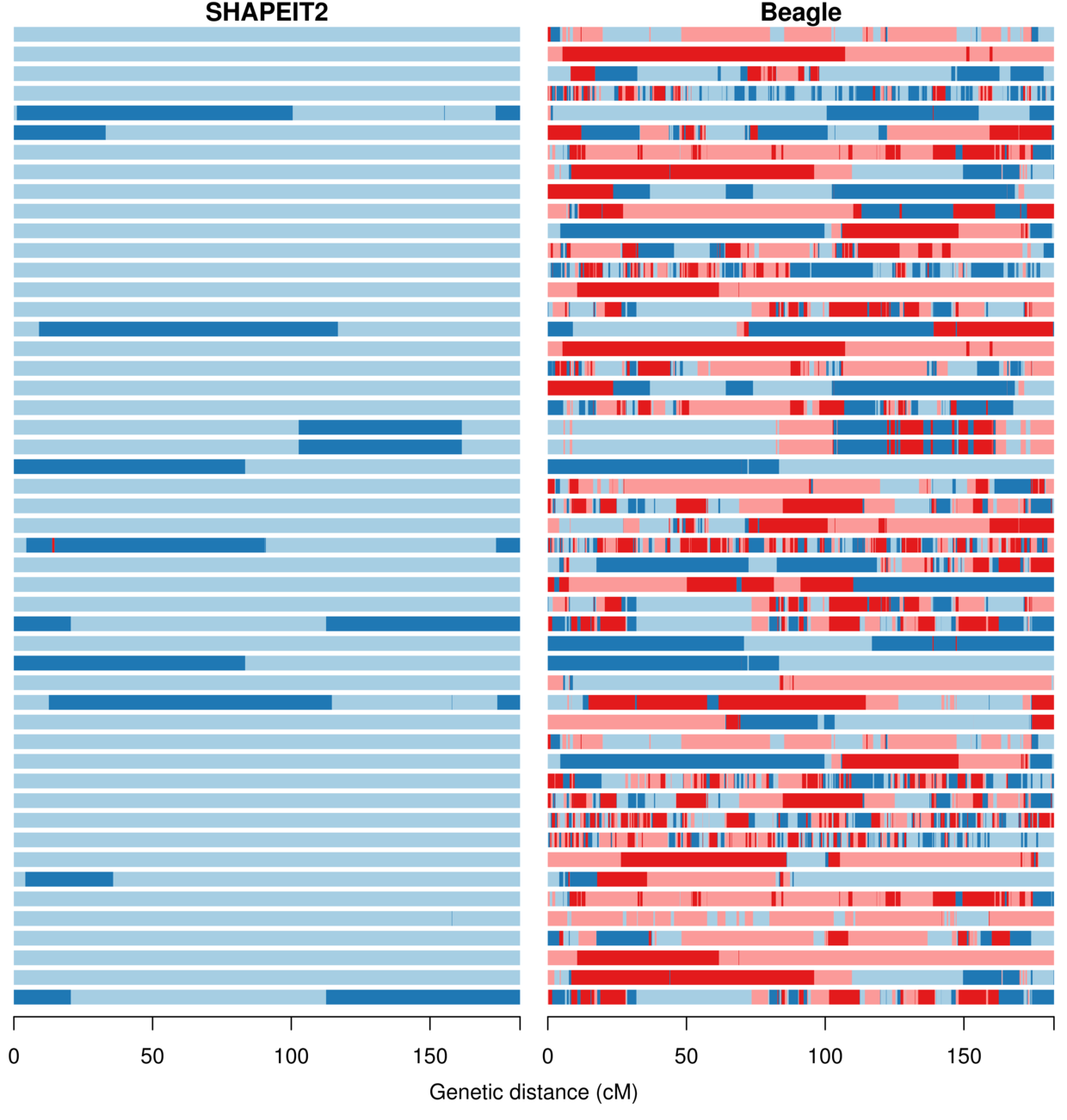

Supplement: Figure S12 — The duo HMM Viterbi paths for 50 father-child duos from the CROATIA-Korcula cohort on chromosome 10. The four possible IBD states (A, B, C, D) are shown using colours pale blue, dark blue, light red and dark red respectively. The left and right panels show the results of the duo HMM applied to the SHAPEIT2 and Beagle haplotypes respectively. Changes between a blue and red colour correspond to a or transition, both of which imply a switch error in the child. Changes of colour between light and dark blue or between light and dark red correspond to transitions, which correspond to a change on IBD state in the parent, and could be caused by a recombination or a switch error in the parent. The x-axis shows the sex-averaged genetic distance across the chromosome in centiMorgans. (TIFF) [file pgen.1004234.s012.tif]

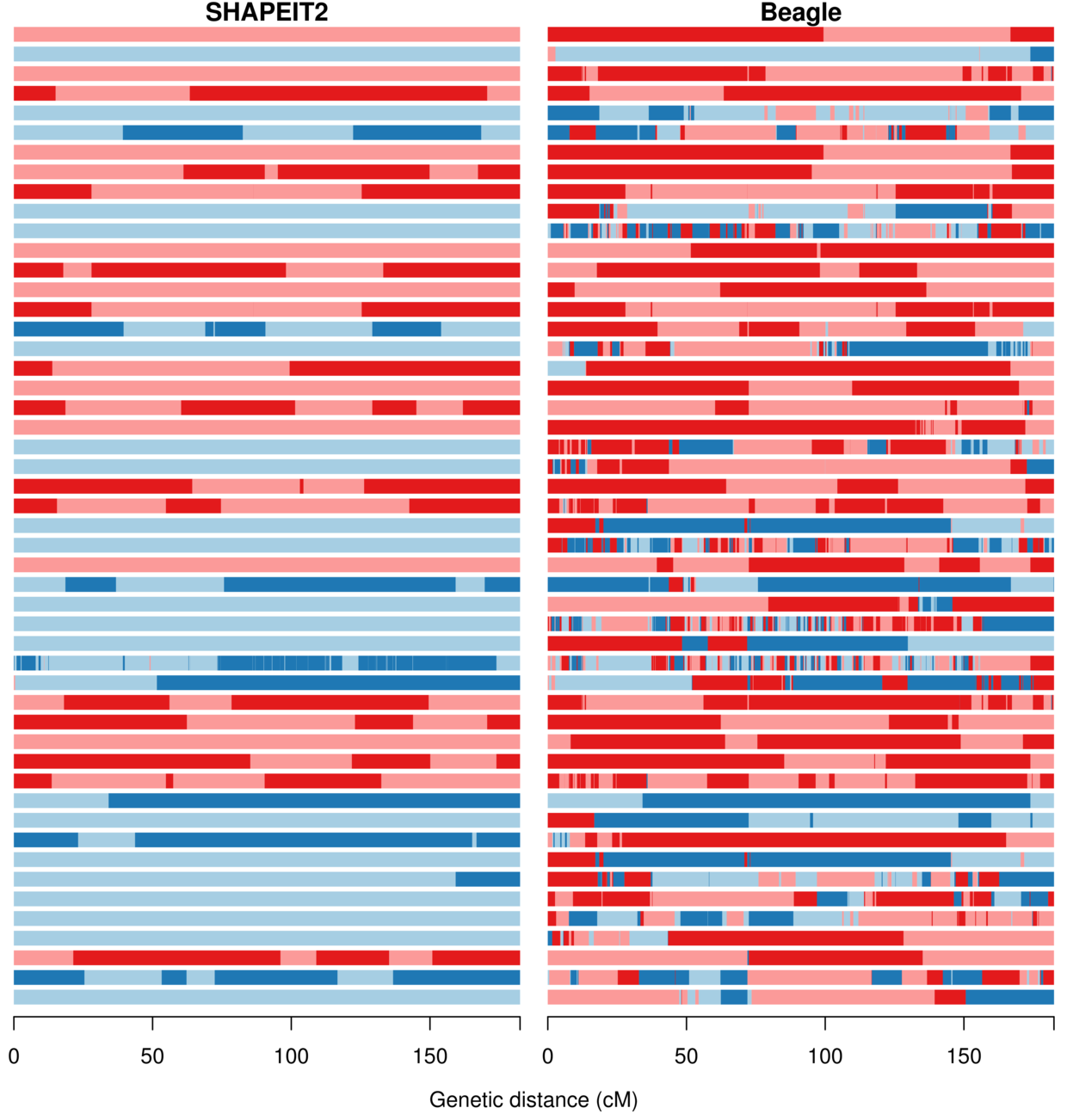

Supplement: Figure S13 — The duo HMM Viterbi paths for 50 mother-child duos from the ORCADES cohort on chromosome 10. The four possible IBD states (A, B, C, D) are shown using colours pale blue, dark blue, light red and dark red respectively. The left and right panels show the results of the duo HMM applied to the SHAPEIT2 and Beagle haplotypes respectively. Changes between a blue and red colour correspond to a or transition, both of which imply a switch error in the child. Changes of colour between light and dark blue or between light and dark red correspond to transitions, which correspond to a change on IBD state in the parent, and could be caused by a recombination or a switch error in the parent. The x-axis shows the sex-averaged genetic distance across the chromosome in centiMorgans. (TIFF) [file pgen.1004234.s013.tif]

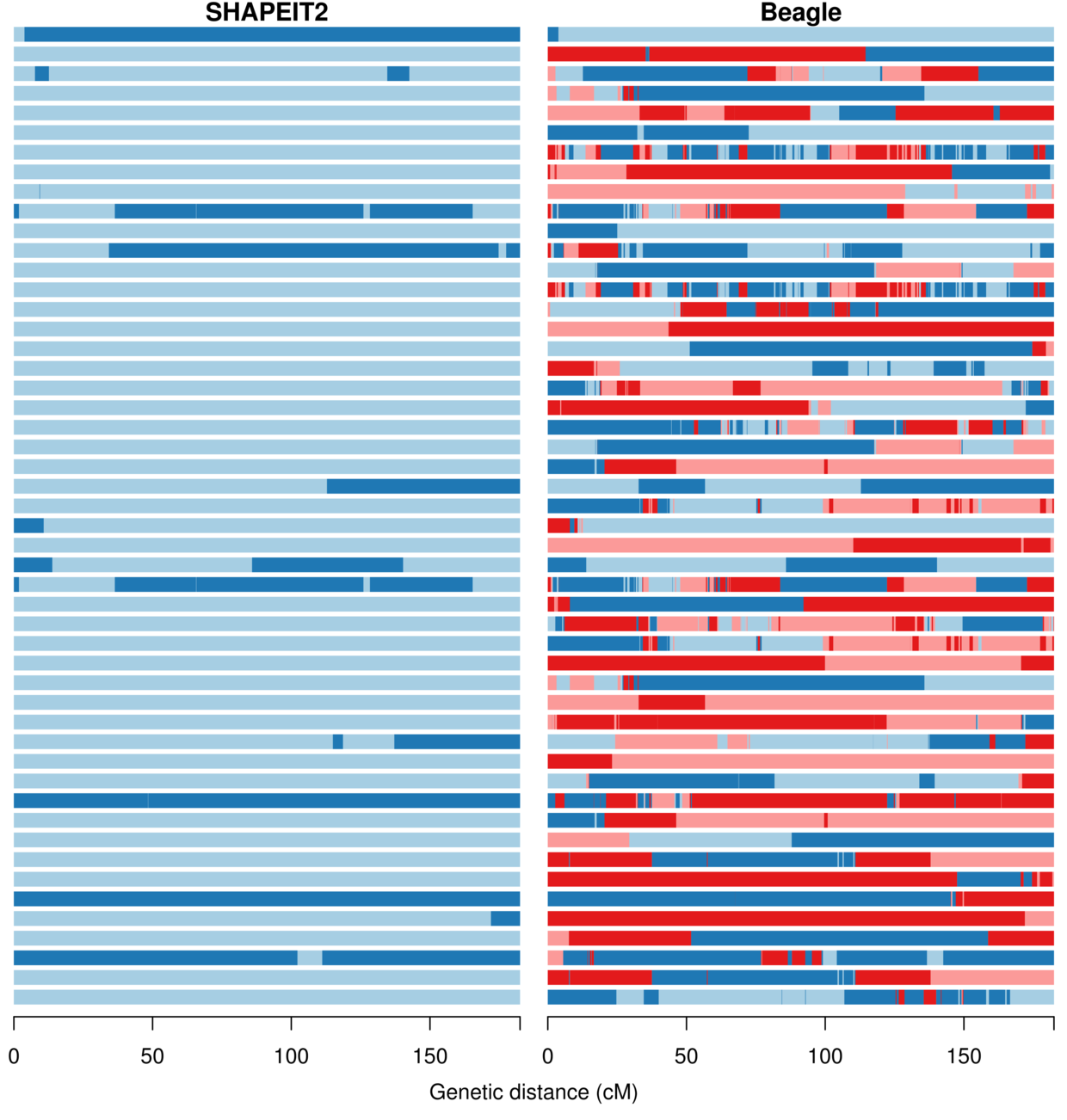

Supplement: Figure S14 — The duo HMM Viterbi paths for 50 father-child duos from the ORCADES cohort on chromosome 10. The four possible IBD states (A, B, C, D) are shown using colours pale blue, dark blue, light red and dark red respectively. The left and right panels show the results of the duo HMM applied to the SHAPEIT2 and Beagle haplotypes respectively. Changes between a blue and red colour correspond to a or transition, both of which imply a switch error in the child. Changes of colour between light and dark blue or between light and dark red correspond to transitions, which correspond to a change on IBD state in the parent, and could be caused by a recombination or a switch error in the parent. The x-axis shows the sex-averaged genetic distance across the chromosome in centiMorgans. (TIFF) [file pgen.1004234.s014.tif]

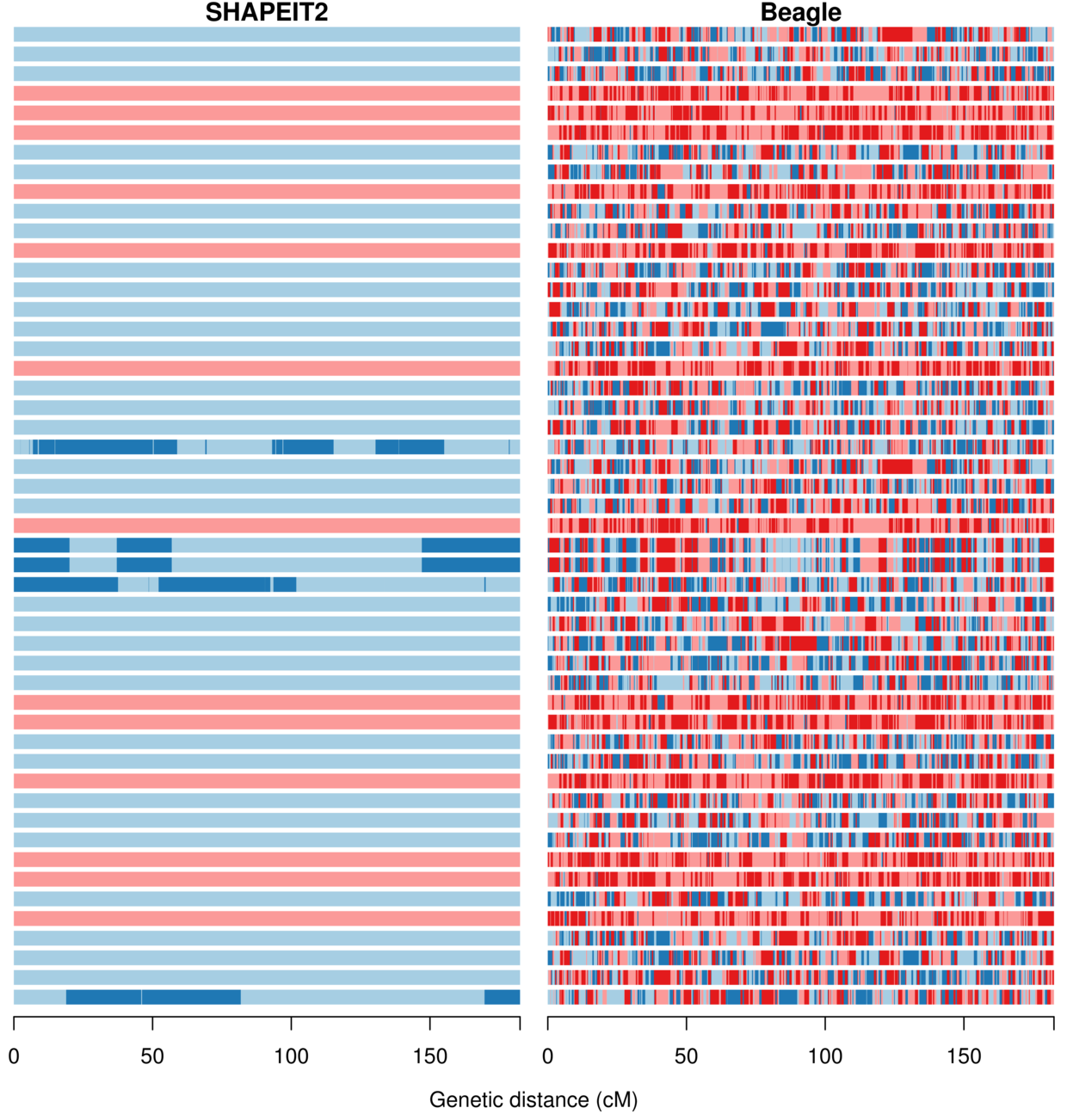

Supplement: Figure S15 — The duo HMM Viterbi paths for 50 mother-child duos from the CROATIA-Split cohort on chromosome 10. The four possible IBD states (A, B, C, D) are shown using colours pale blue, dark blue, light red and dark red respectively. The left and right panels show the results of the duo HMM applied to the SHAPEIT2 and Beagle haplotypes respectively. Changes between a blue and red colour correspond to a or transition, both of which imply a switch error in the child. Changes of colour between light and dark blue or between light and dark red correspond to transitions, which correspond to a change on IBD state in the parent, and could be caused by a recombination or a switch error in the parent. The x-axis shows the sex-averaged genetic distance across the chromosome in centiMorgans. (TIFF) [file pgen.1004234.s015.tif]

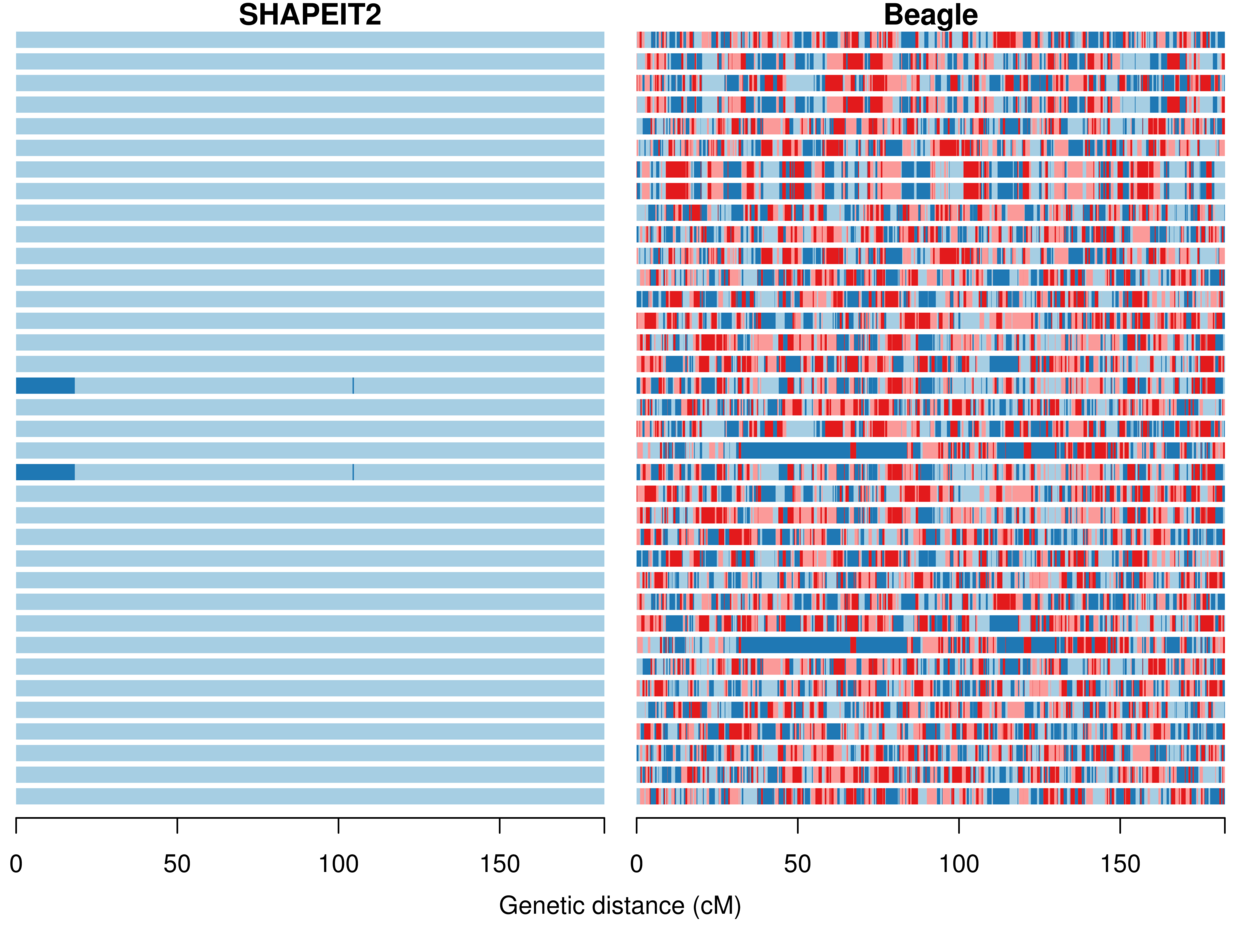

Supplement: Figure S16 — The duo HMM Viterbi paths for 50 father-child duos from the CROATIA-Split cohort on chromosome 10. The four possible IBD states (A, B, C, D) are shown using colours pale blue, dark blue, light red and dark red respectively. The left and right panels show the results of the duo HMM applied to the SHAPEIT2 and Beagle haplotypes respectively. Changes between a blue and red colour correspond to a or transition, both of which imply a switch error in the child. Changes of colour between light and dark blue or between light and dark red correspond to transitions, which correspond to a change on IBD state in the parent, and could be caused by a recombination or a switch error in the parent. The x-axis shows the sex-averaged genetic distance across the chromosome in centiMorgans. (TIFF) [file pgen.1004234.s016.tif]

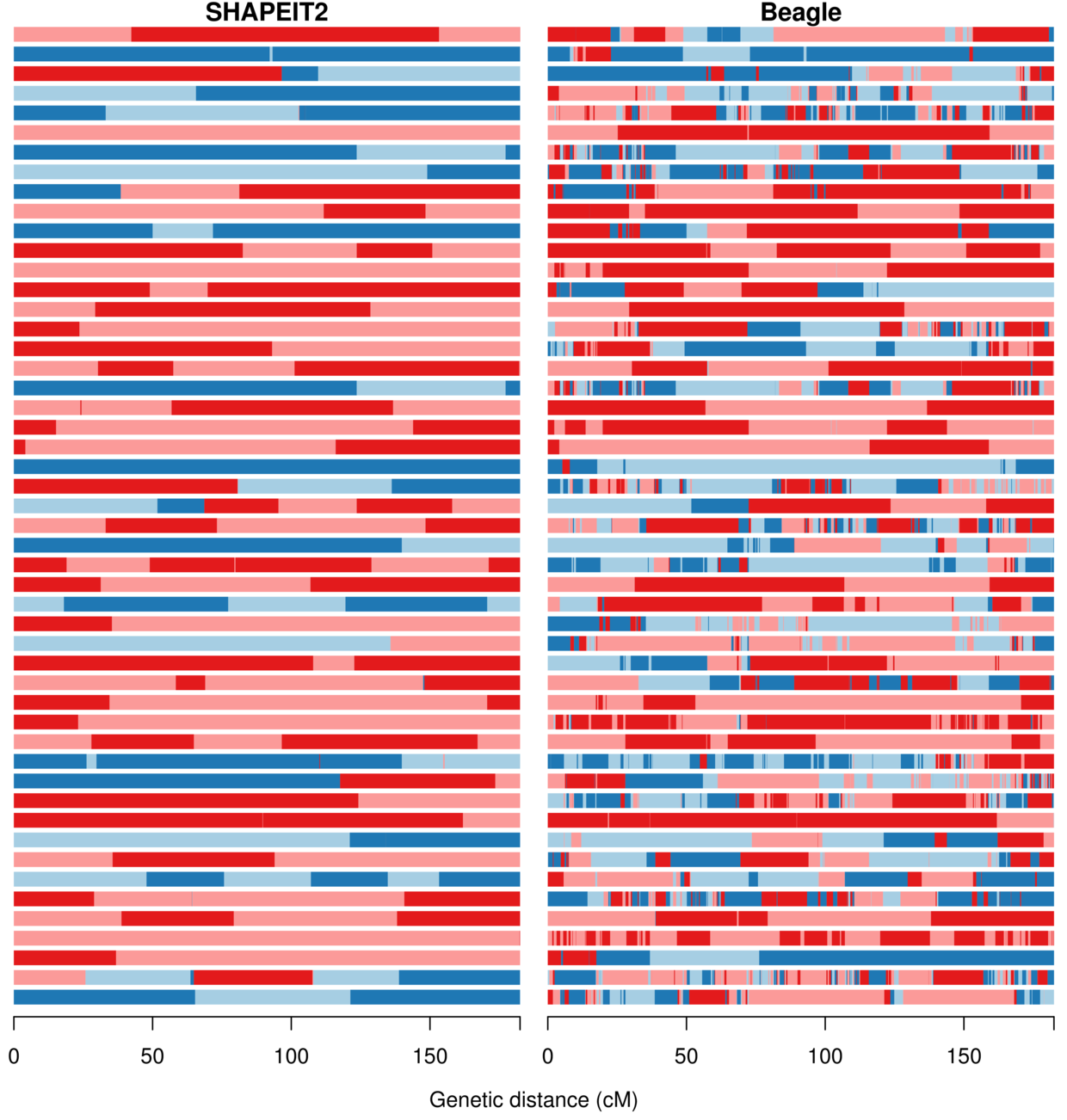

Supplement: Figure S17 — The duo HMM Viterbi paths for 50 mother-child duos from the Val Borbera cohort on chromosome 10. The four possible IBD states (A, B, C, D) are shown using colours pale blue, dark blue, light red and dark red respectively. The left and right panels show the results of the duo HMM applied to the SHAPEIT2 and Beagle haplotypes respectively. Changes between a blue and red colour correspond to a or transition, both of which imply a switch error in the child. Changes of colour between light and dark blue or between light and dark red correspond to transitions, which correspond to a change on IBD state in the parent, and could be caused by a recombination or a switch error in the parent. The x-axis shows the sex-averaged genetic distance across the chromosome in centiMorgans. (TIFF) [file pgen.1004234.s017.tif]

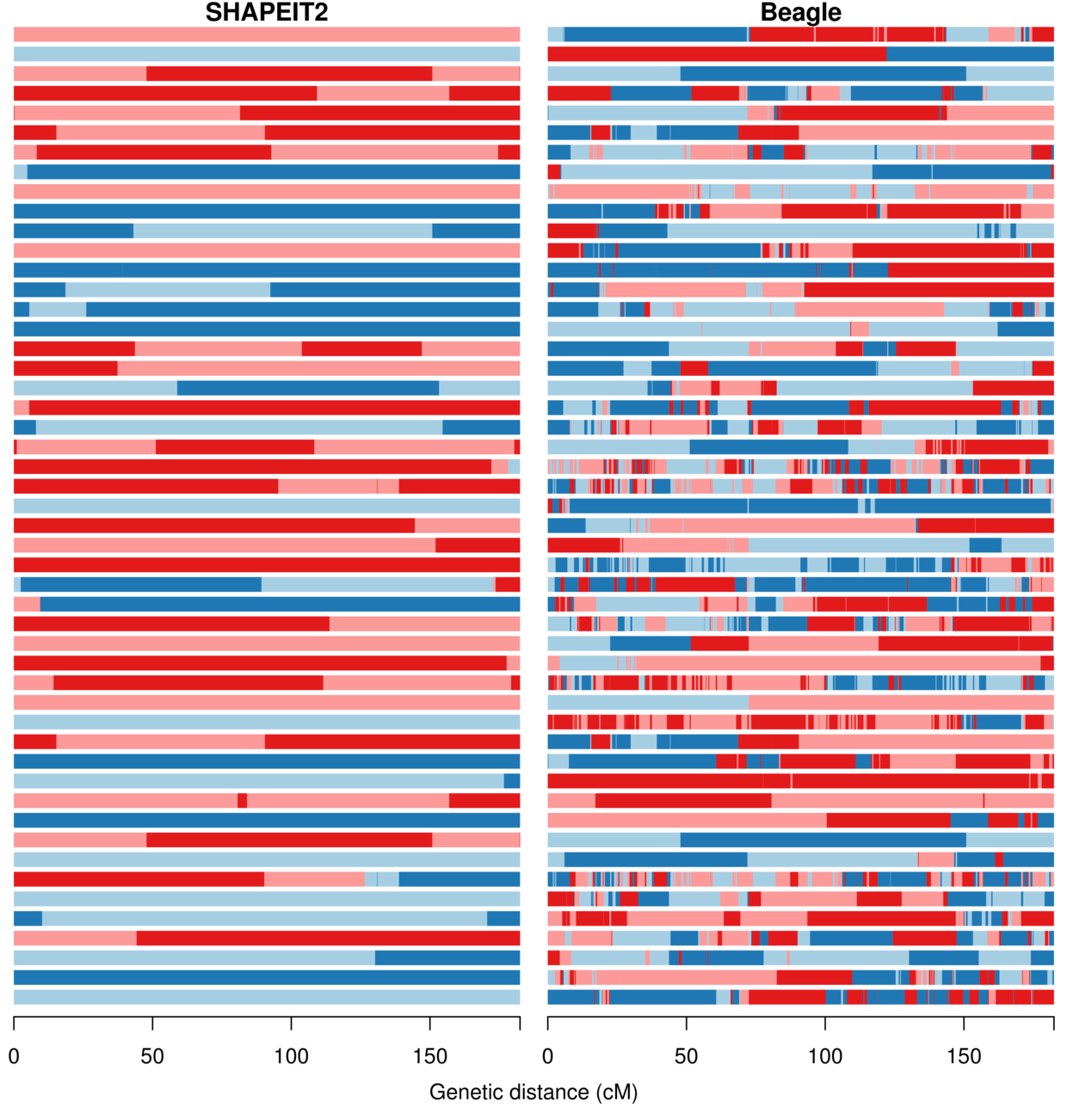

Supplement: Figure S18 — The duo HMM Viterbi paths for 50 father-child duos from the Val Borbera cohort on chromosome 10. The four possible IBD states (A, B, C, D) are shown using colours pale blue, dark blue, light red and dark red respectively. The left and right panels show the results of the duo HMM applied to the SHAPEIT2 and Beagle haplotypes respectively. Changes between a blue and red colour correspond to a or transition, both of which imply a switch error in the child. Changes of colour between light and dark blue or between light and dark red correspond to transitions, which correspond to a change on IBD state in the parent, and could be caused by a recombination or a switch error in the parent. The x-axis shows the sex-averaged genetic distance across the chromosome in centiMorgans. (TIFF) [file pgen.1004234.s018.tif]

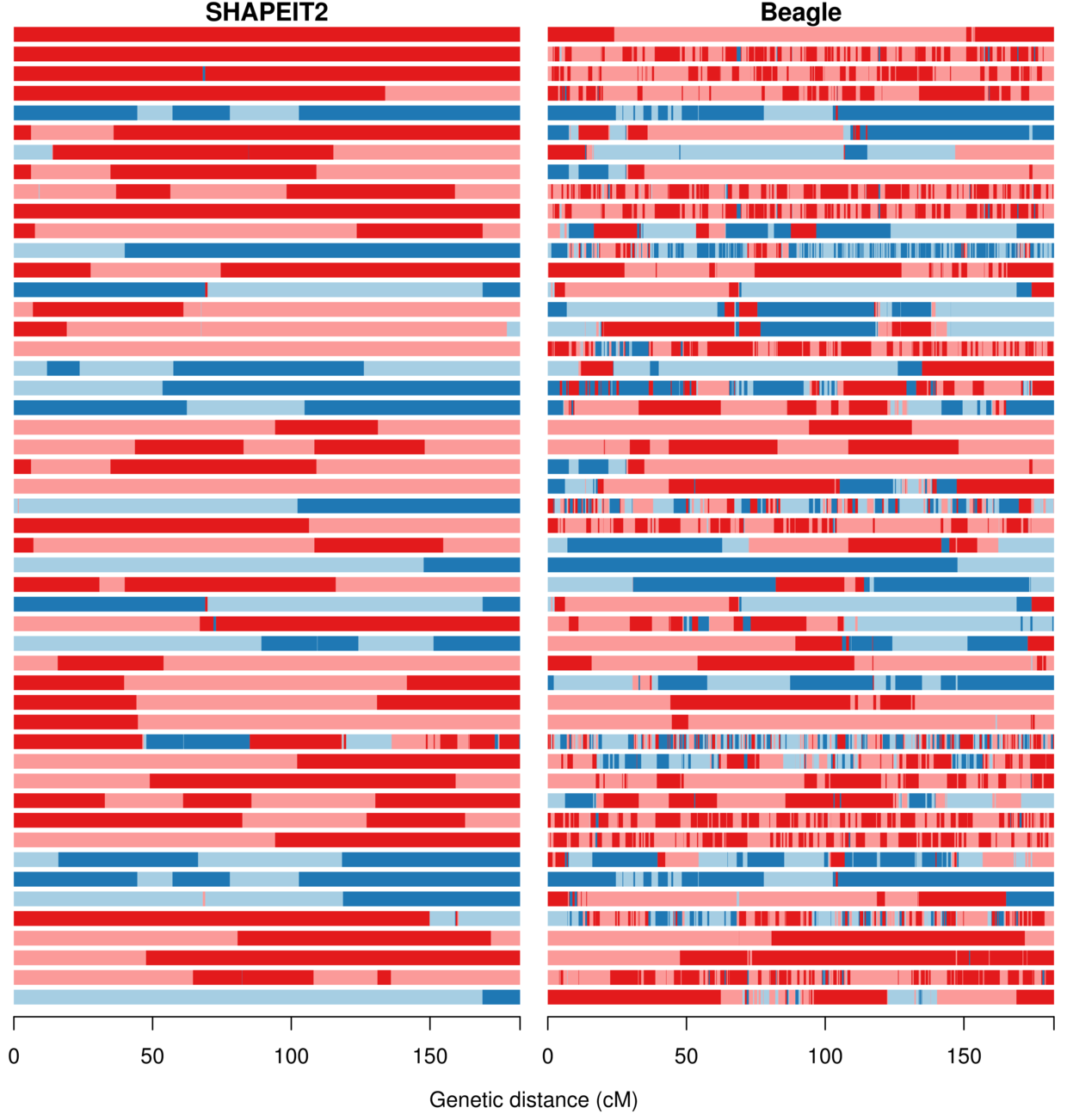

Supplement: Figure S19 — The duo HMM Viterbi paths for 50 mother-child duos from the CROATIA-Vis cohort on chromosome 10. The four possible IBD states (A, B, C, D) are shown using colours pale blue, dark blue, light red and dark red respectively. The left and right panels show the results of the duo HMM applied to the SHAPEIT2 and Beagle haplotypes respectively. Changes between a blue and red colour correspond to a or transition, both of which imply a switch error in the child. Changes of colour between light and dark blue or between light and dark red correspond to transitions, which correspond to a change on IBD state in the parent, and could be caused by a recombination or a switch error in the parent. The x-axis shows the sex-averaged genetic distance across the chromosome in centiMorgans. (TIFF) [file pgen.1004234.s019.tif]

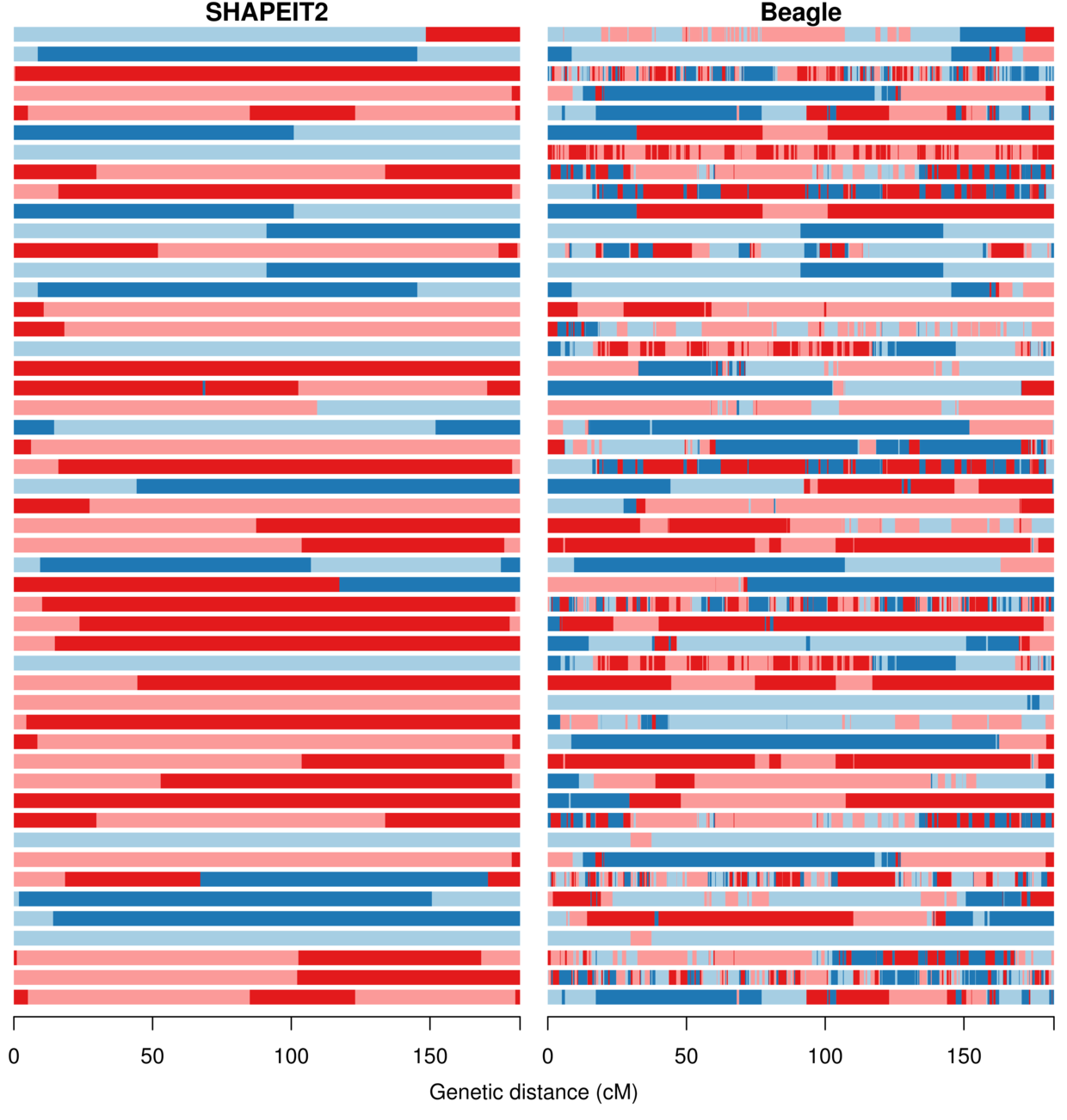

Supplement: Figure S20 — The duo HMM Viterbi paths for 50 father-child duos from the CROATIA-Vis cohort on chromosome 10. The four possible IBD states (A, B, C, D) are shown using colours pale blue, dark blue, light red and dark red respectively. The left and right panels show the results of the duo HMM applied to the SHAPEIT2 and Beagle haplotypes respectively. Changes between a blue and red colour correspond to a or transition, both of which imply a switch error in the child. Changes of colour between light and dark blue or between light and dark red correspond to transitions, which correspond to a change on IBD state in the parent, and could be caused by a recombination or a switch error in the parent. The x-axis shows the sex-averaged genetic distance across the chromosome in centiMorgans. (TIFF) [file pgen.1004234.s020.tif]

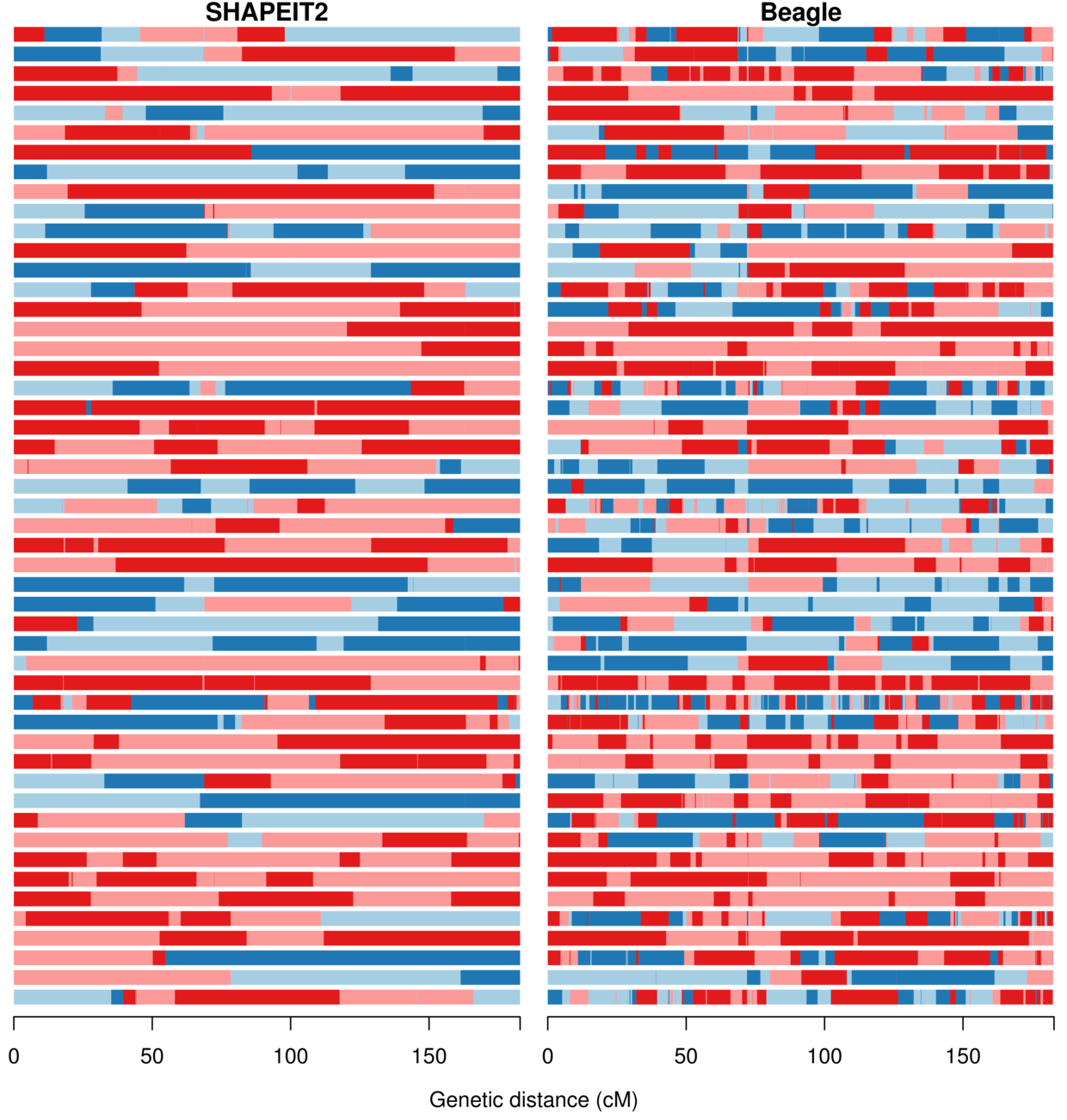

Supplement: Figure S21 — The duo HMM Viterbi paths for 50 mother-child duos from the GPC cohort on chromosome 10. The four possible IBD states (A, B, C, D) are shown using colours pale blue, dark blue, light red and dark red respectively. The left and right panels show the results of the duo HMM applied to the SHAPEIT2 and Beagle haplotypes respectively. Changes between a blue and red colour correspond to a or transition, both of which imply a switch error in the child. Changes of colour between light and dark blue or between light and dark red correspond to transitions, which correspond to a change on IBD state in the parent, and could be caused by a recombination or a switch error in the parent. The x-axis shows the sex-averaged genetic distance across the chromosome in centiMorgans. (TIFF) [file pgen.1004234.s021.tif]

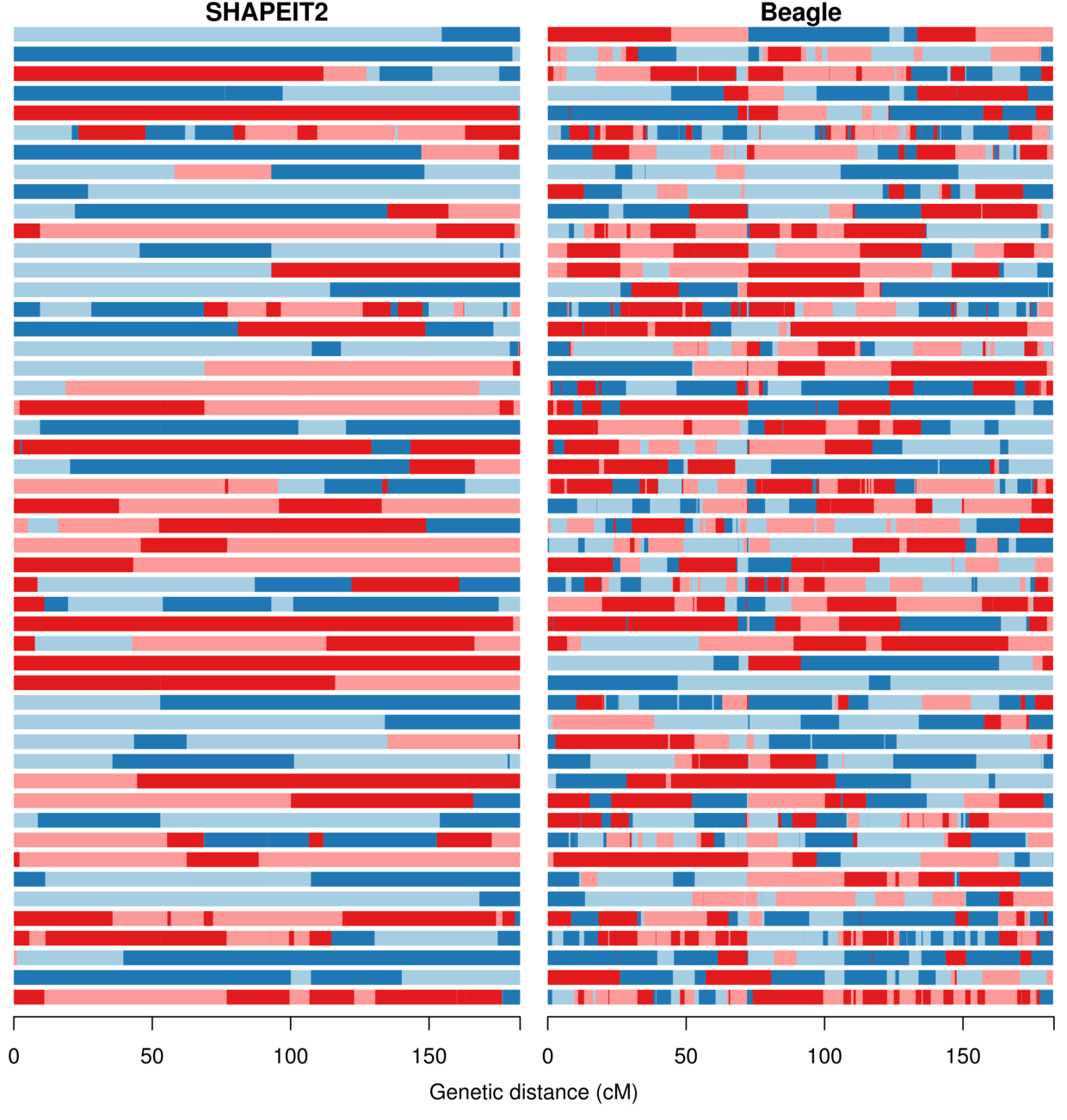

Supplement: Figure S22 — The duo HMM Viterbi paths for 50 father-child duos from the GPC cohort on chromosome 10. The four possible IBD states (A, B, C, D) are shown using colours pale blue, dark blue, light red and dark red respectively. The left and right panels show the results of the duo HMM applied to the SHAPEIT2 and Beagle haplotypes respectively. Changes between a blue and red colour correspond to a or transition, both of which imply a switch error in the child. Changes of colour between light and dark blue or between light and dark red correspond to transitions, which correspond to a change on IBD state in the parent, and could be caused by a recombination or a switch error in the parent. The x-axis shows the sex-averaged genetic distance across the chromosome in centiMorgans. (TIFF) [file pgen.1004234.s022.tif]

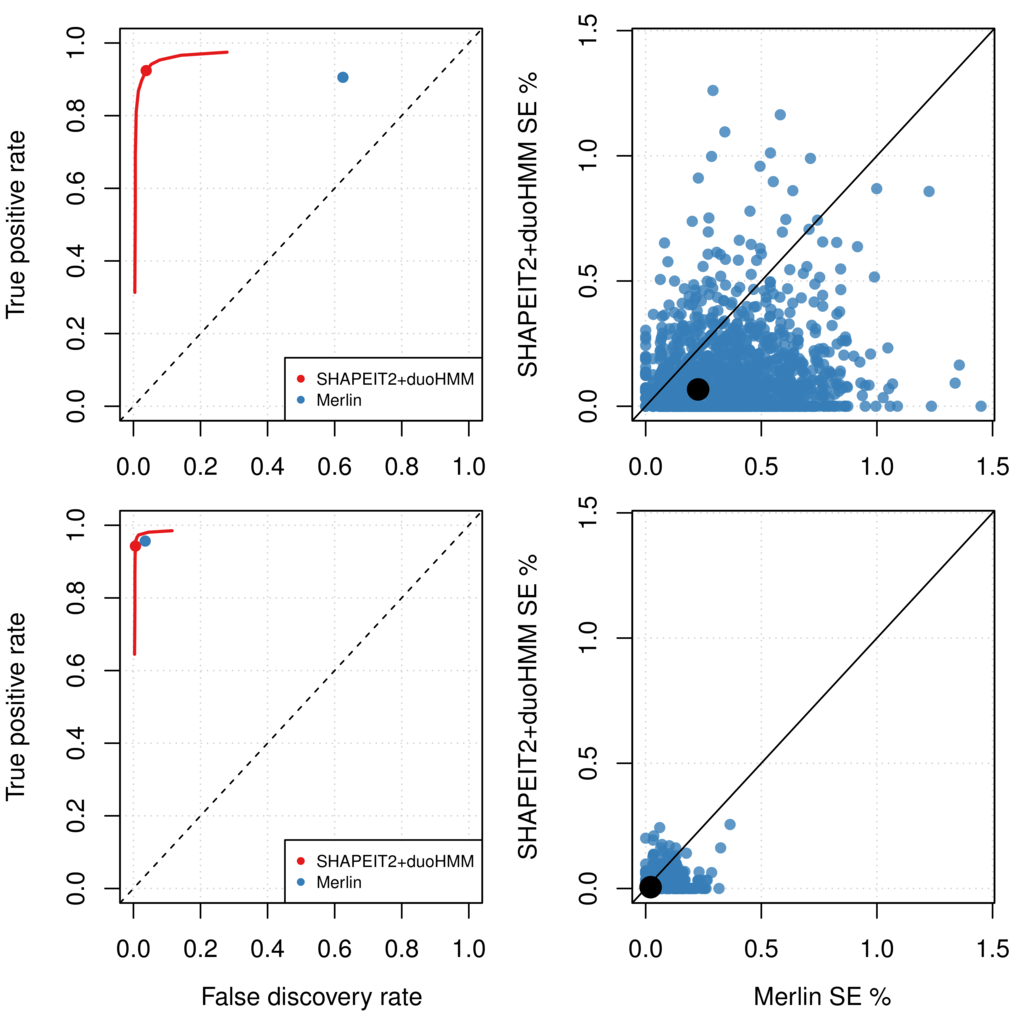

Supplement: Figure S23 — Detection of recombination in simulated extended pedigrees and comparison of SHAPEIT2 and Merlin haplotype accuracy for realistic data (top) and ideal data (bottom). Left: True positive rate (TPR) versus false discovery rate (FDR) for recombination detection using SHAPEIT2+duoHMM (red) versus Merlin (blue). Merlin detects 90.57% of crossovers with a substantial FDR of 62.48% whilst SHAPEIT2+duoHMM detects 92.40% of events with an FDR of 3.78% (the red point at ). On ideal data SHAPEIT2+duoHMM achieve a 95.75% TPR and 0.71% FDR and Merlin has 95.66% and 3.48% respectively. Right: Switch error for SHAPEIT2 (DuoHMM corrected) versus Merlin. The black point is the mean for each method. Merlin had 0.215% (0.021% ideal scenario) switch error while SHAPEIT2 had a rate of 0.033% (0.005% ideal scenario). (TIFF) [file pgen.1004234.s023.tif]

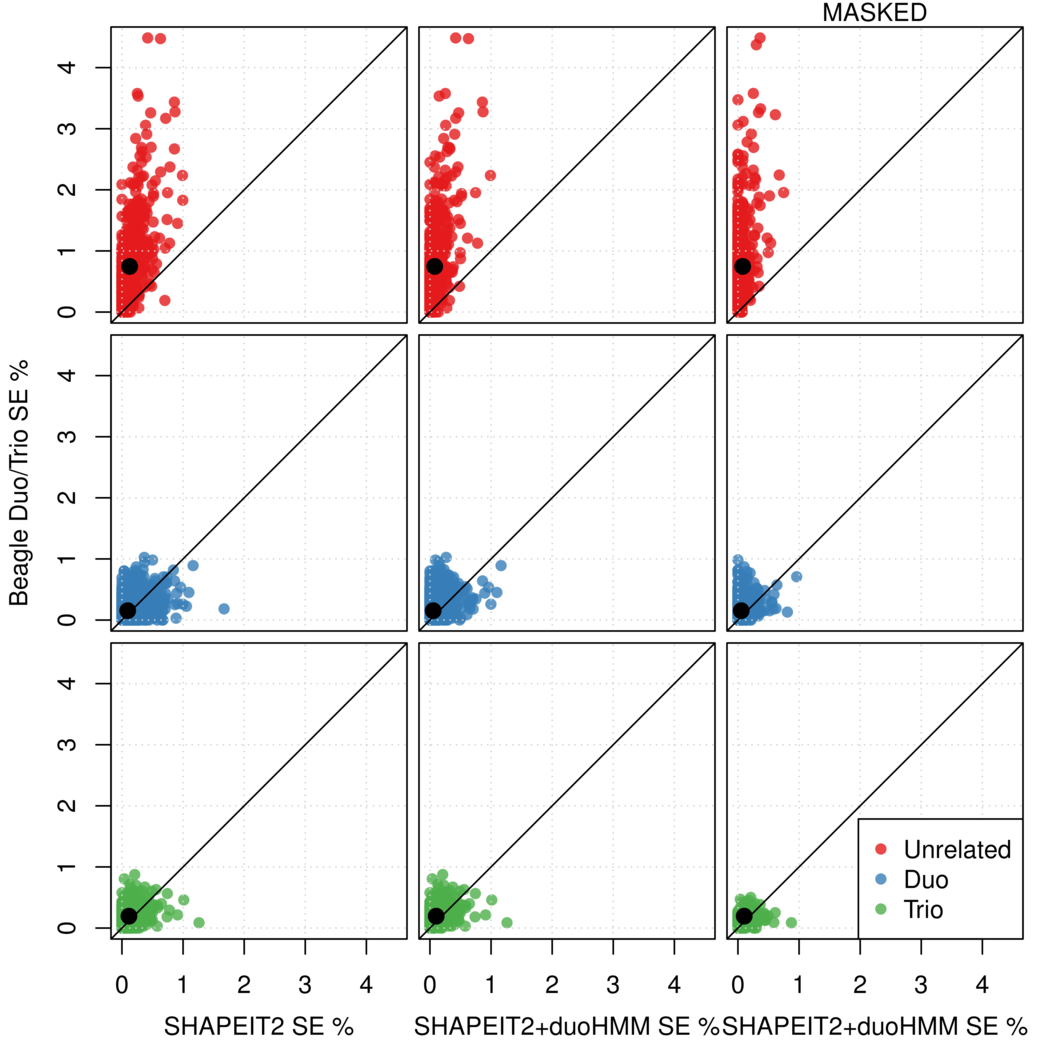

Supplement: Figure S24 — Switch error results on simulated data for extended pedigrees. Points are coloured according to what family information was used by Beagle in duo/trio mode; red meaning an individual could not be included in a duo or trio. Left: Beagle duo/trio switch error rate (0.269% average SE) against switch error rate for SHAPEIT2 when all individuals are treated as unrelated (0.104% average SE). Centre: After applying the duoHMM haplotype corrections to SHAPEIT2 (0.065% average SE). Right: Switch error after masking genotypes flagged as erroneous by the SHAPEIT2+duoHMM, Beagle duo/trio phasing is reduced to 0.231% and SHAPEIT2+duoHMM to 0.034%. (TIFF) [file pgen.1004234.s024.tif]

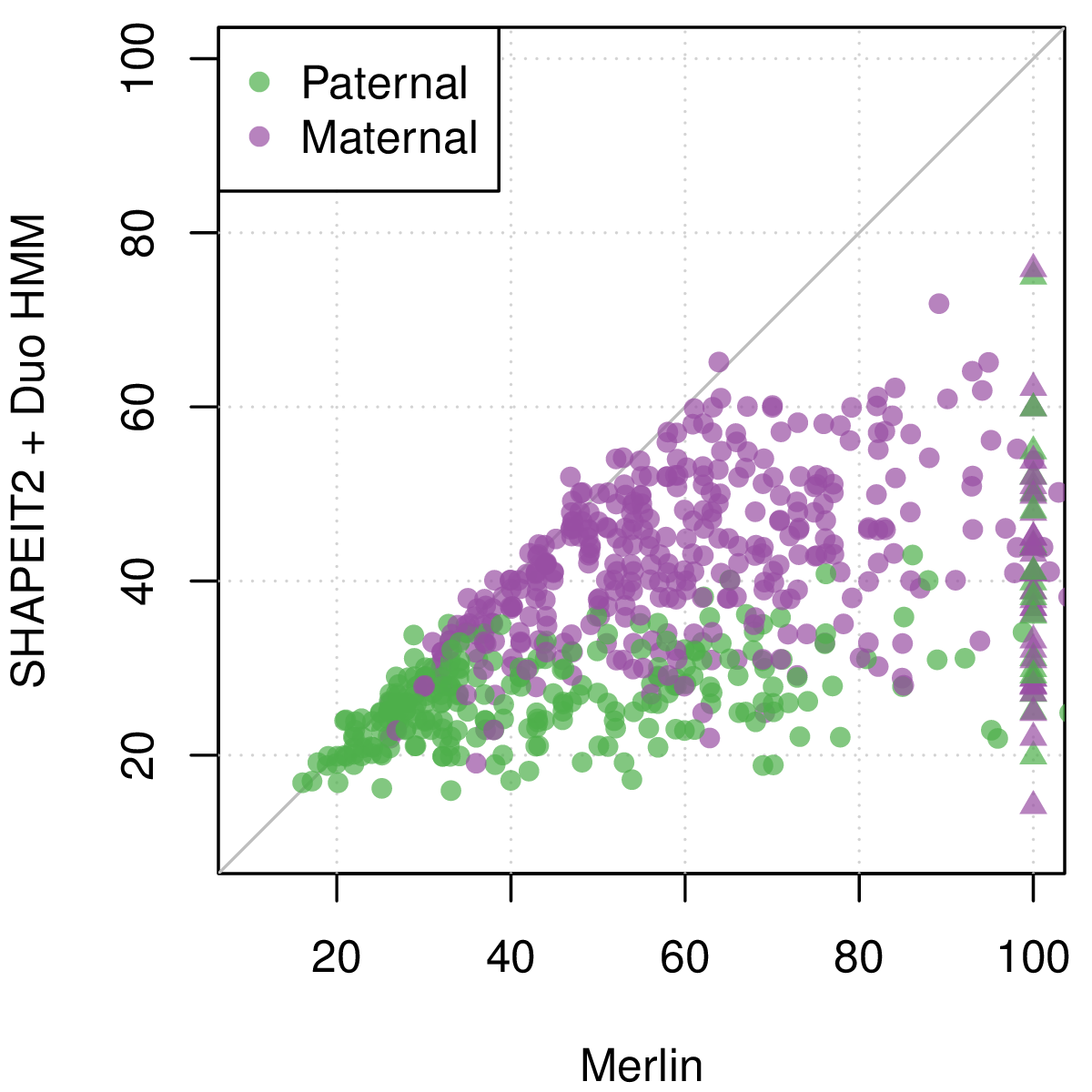

Supplement: Figure S25 — Genome-wide number of recombinations found per individual for SHAPEIT versus Merlin for all informative meioses in real data sets. The number of recombinations found by SHAPEIT2 against Merlin for each of 661 meioses (all informative duos from all cohorts). Maternal are in green and paternal purple. Triangles represent a truncated value. While there is correlation between the two methods, Merlin more frequently finds an implausible number of recombinations. (TIFF) [file pgen.1004234.s025.tif]

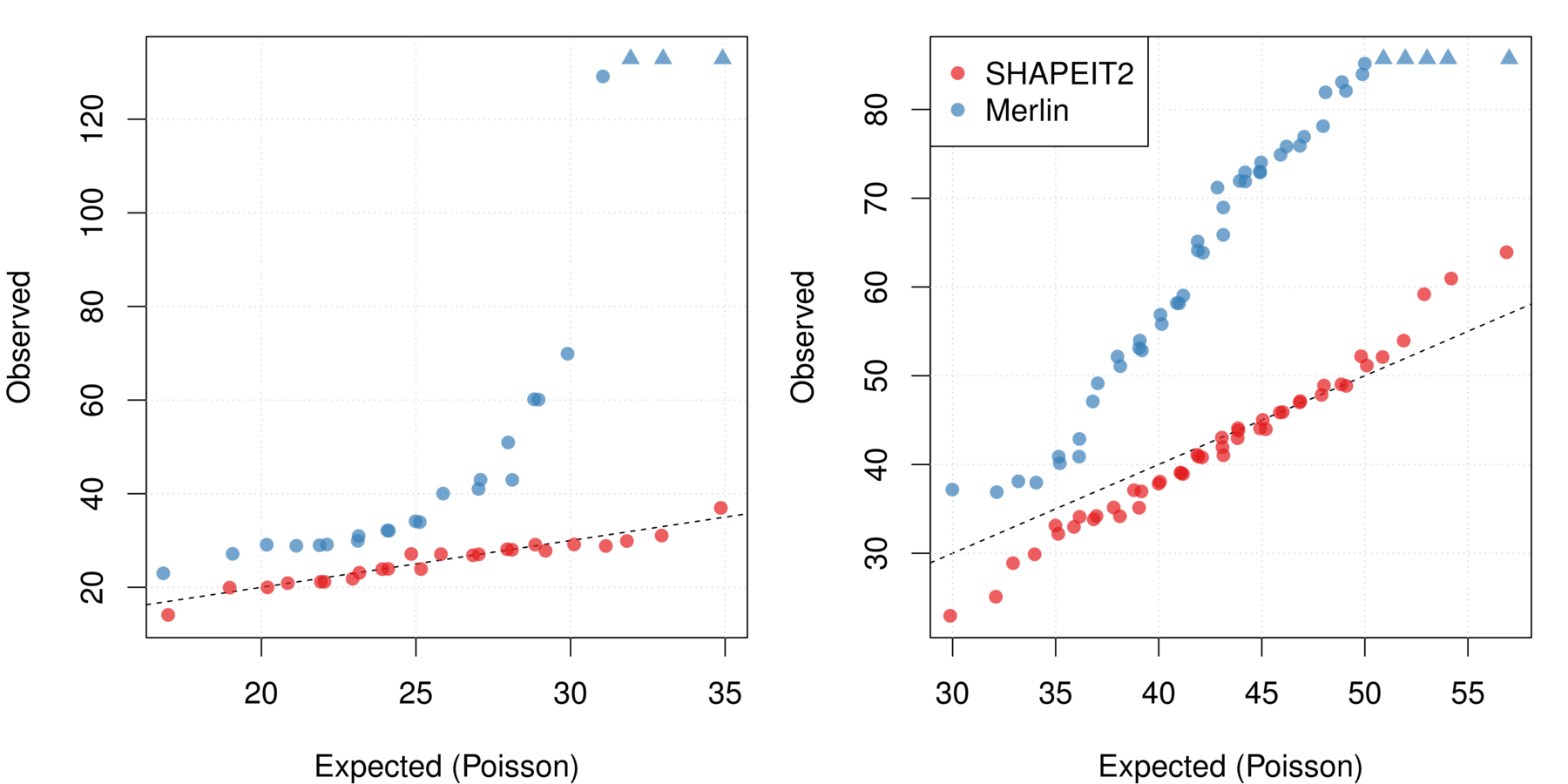

Supplement: Figure S26 — Carlantino cohort recombination distributions. Q-Q plots for the observed against expected number of recombinations estimated by each method for paternal meioses (left) and maternal meioses (right), only duos that were part of an informative pedigree were used. For the expected distribution of recombination rates, a Poisson distribution using the genetic lengths from the 2002 deCODE Map was used (with rate parameter 42.81 and 25.9 for maternal and paternal recombinations respectively.) (TIFF) [file pgen.1004234.s026.tif]

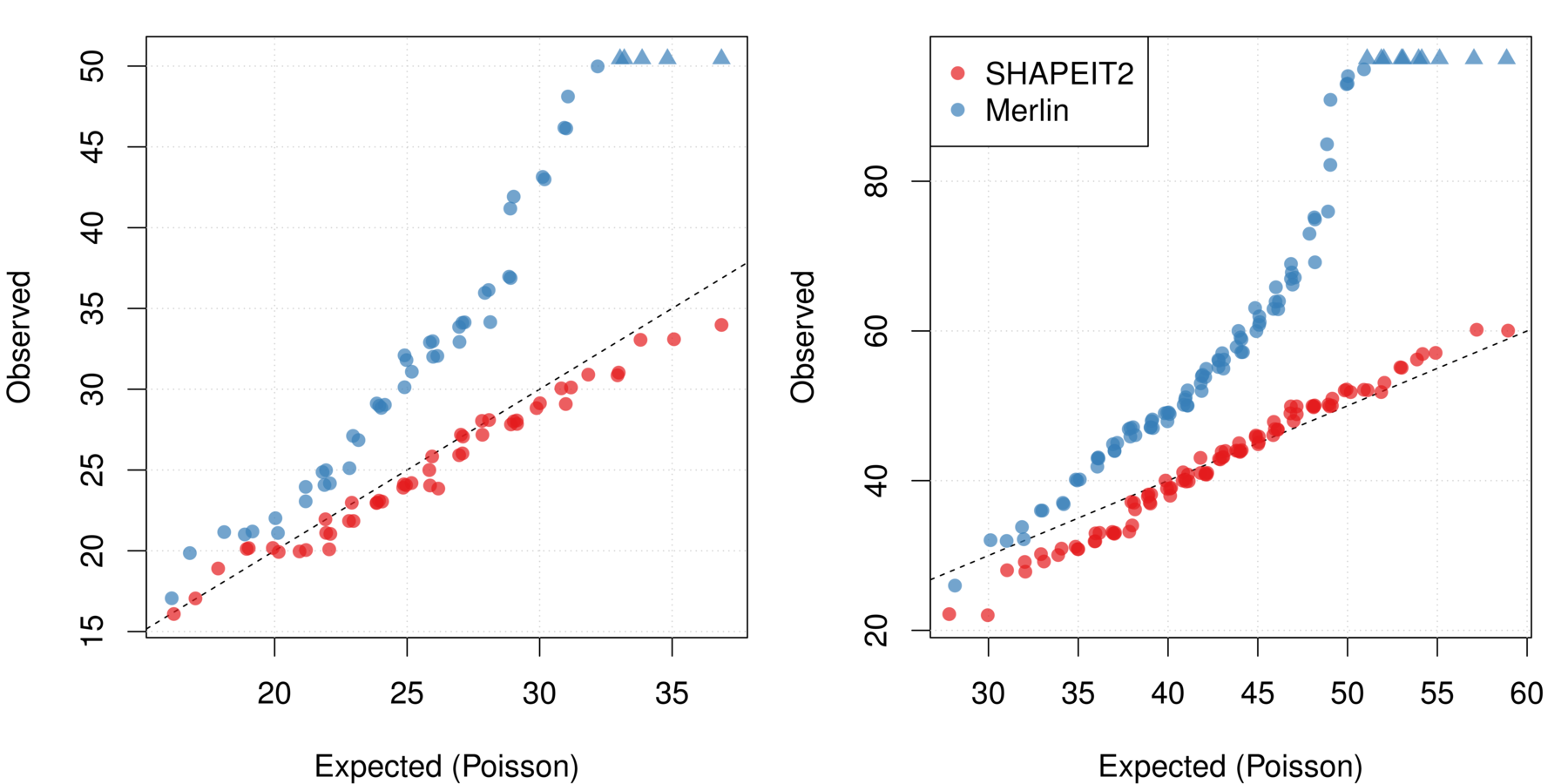

Supplement: Figure S27 — FVG cohort - recombination distributions. Q-Q plots for the observed against expected number of recombinations estimated by each method for paternal meioses (left) and maternal meioses (right), only duos that were part of an informative pedigree were used. For the expected distribution of recombination rates, a Poisson distribution using the genetic lengths from the 2002 deCODE Map was used (with rate parameter 42.81 and 25.9 for maternal and paternal recombinations respectively.) (TIFF) [file pgen.1004234.s027.tif]

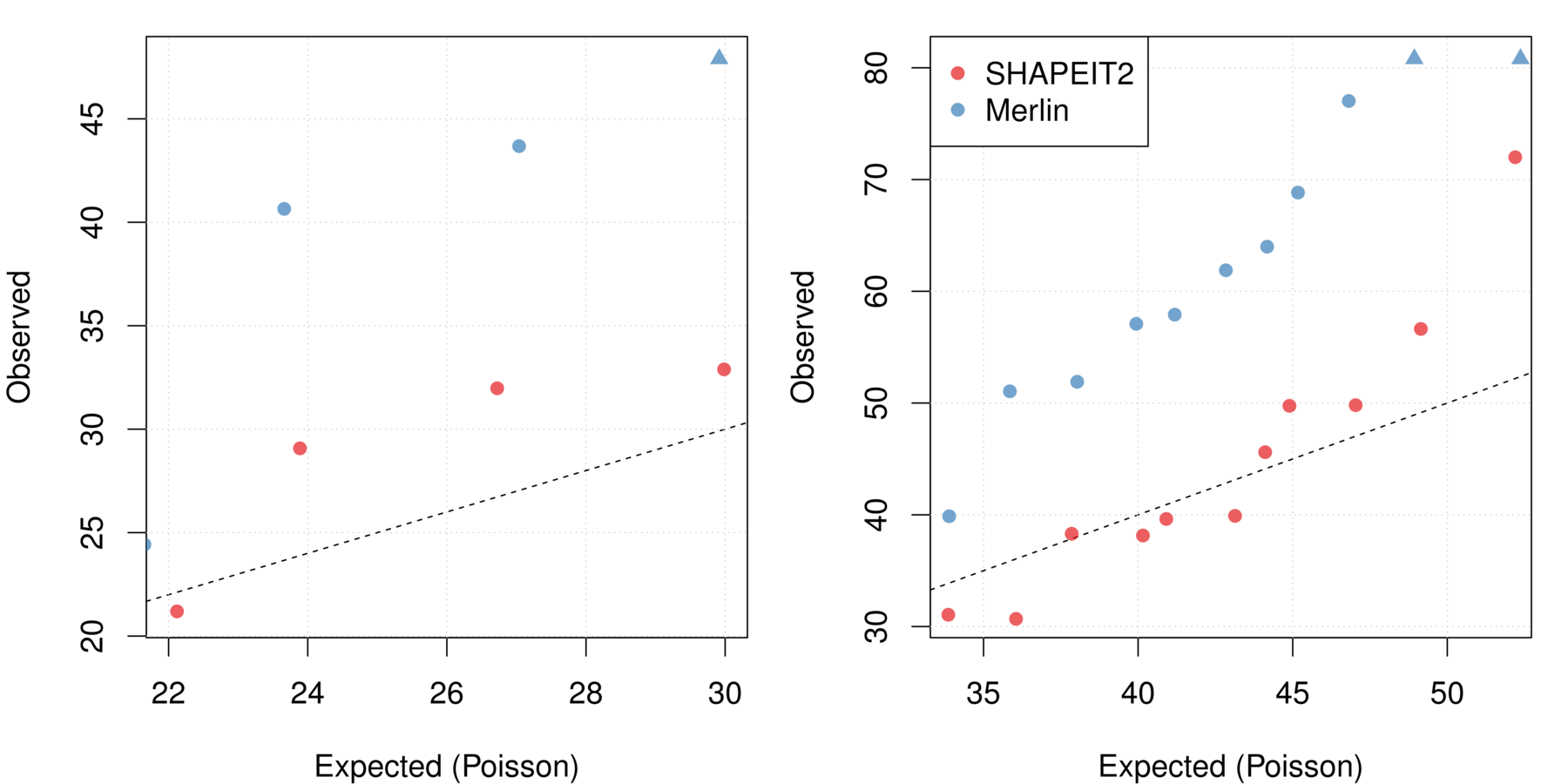

Supplement: Figure S28 — Korcula cohort - recombination distributions. Q-Q plots for the observed against expected number of recombinations estimated by each method for paternal meioses (left) and maternal meioses (right), only duos that were part of an informative pedigree were used. For the expected distribution of recombination rates, a Poisson distribution using the genetic lengths from the 2002 deCODE Map was used (with rate parameter 42.81 and 25.9 for maternal and paternal recombinations respectively.) (TIFF) [file pgen.1004234.s028.tif]

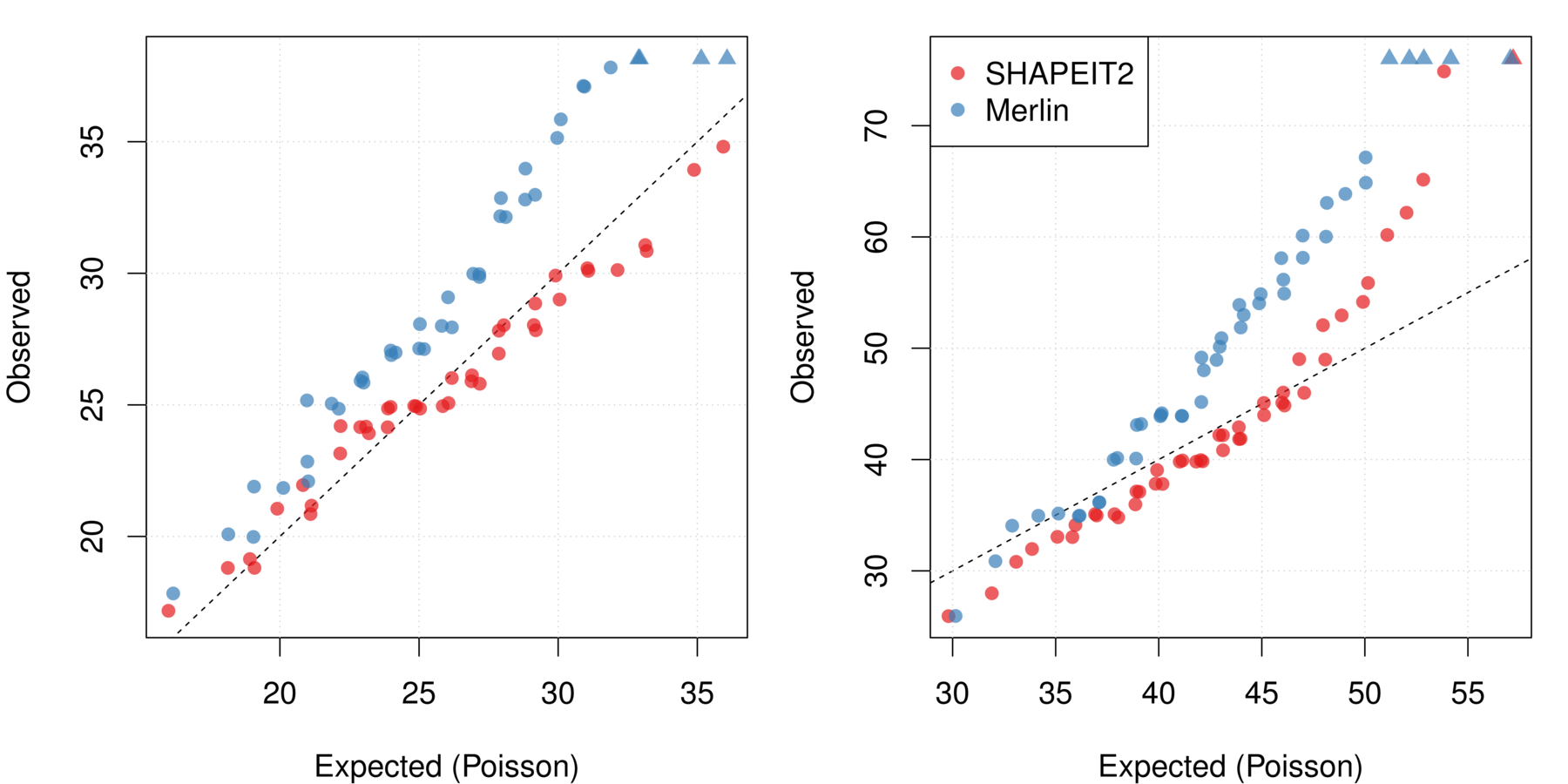

Supplement: Figure S29 — ORCADES cohort - recombination distributions. Q-Q plots for the observed against expected number of recombinations estimated by each method for paternal meioses (left) and maternal meioses (right), only duos that were part of an informative pedigree were used. For the expected distribution of recombination rates, a Poisson distribution using the genetic lengths from the 2002 deCODE Map was used (with rate parameter 42.81 and 25.9 for maternal and paternal recombinations respectively.) (TIFF) [file pgen.1004234.s029.tif]

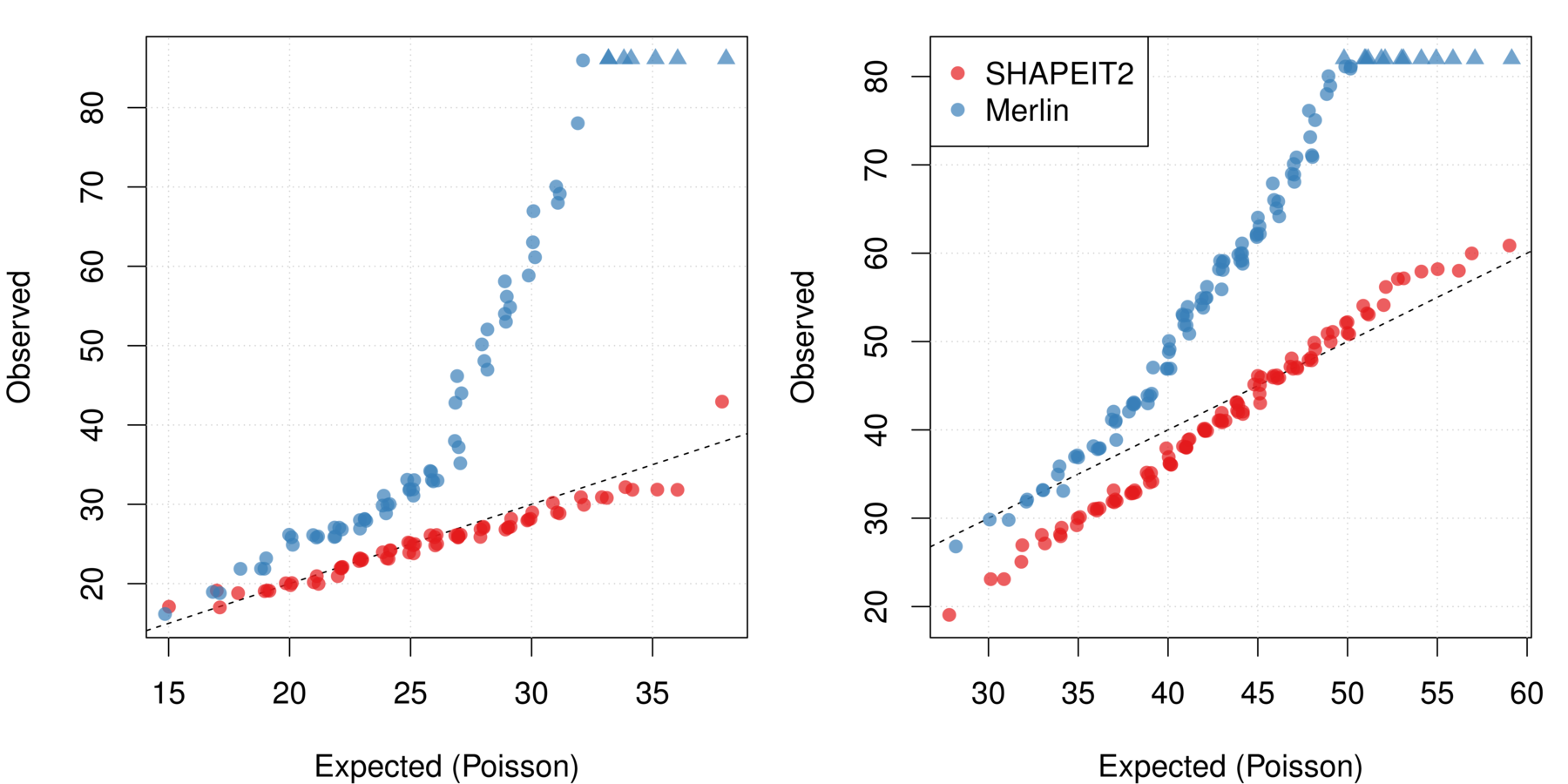

Supplement: Figure S30 — Valborbera cohort - recombination distributions. Q-Q plots for the observed against expected number of recombinations estimated by each method for paternal meioses (left) and maternal meioses (right), only duos that were part of an informative pedigree were used. For the expected distribution of recombination rates, a Poisson distribution using the genetic lengths from the 2002 deCODE Map was used (with rate parameter 42.81 and 25.9 for maternal and paternal recombinations respectively.) (TIFF) [file pgen.1004234.s030.tif]

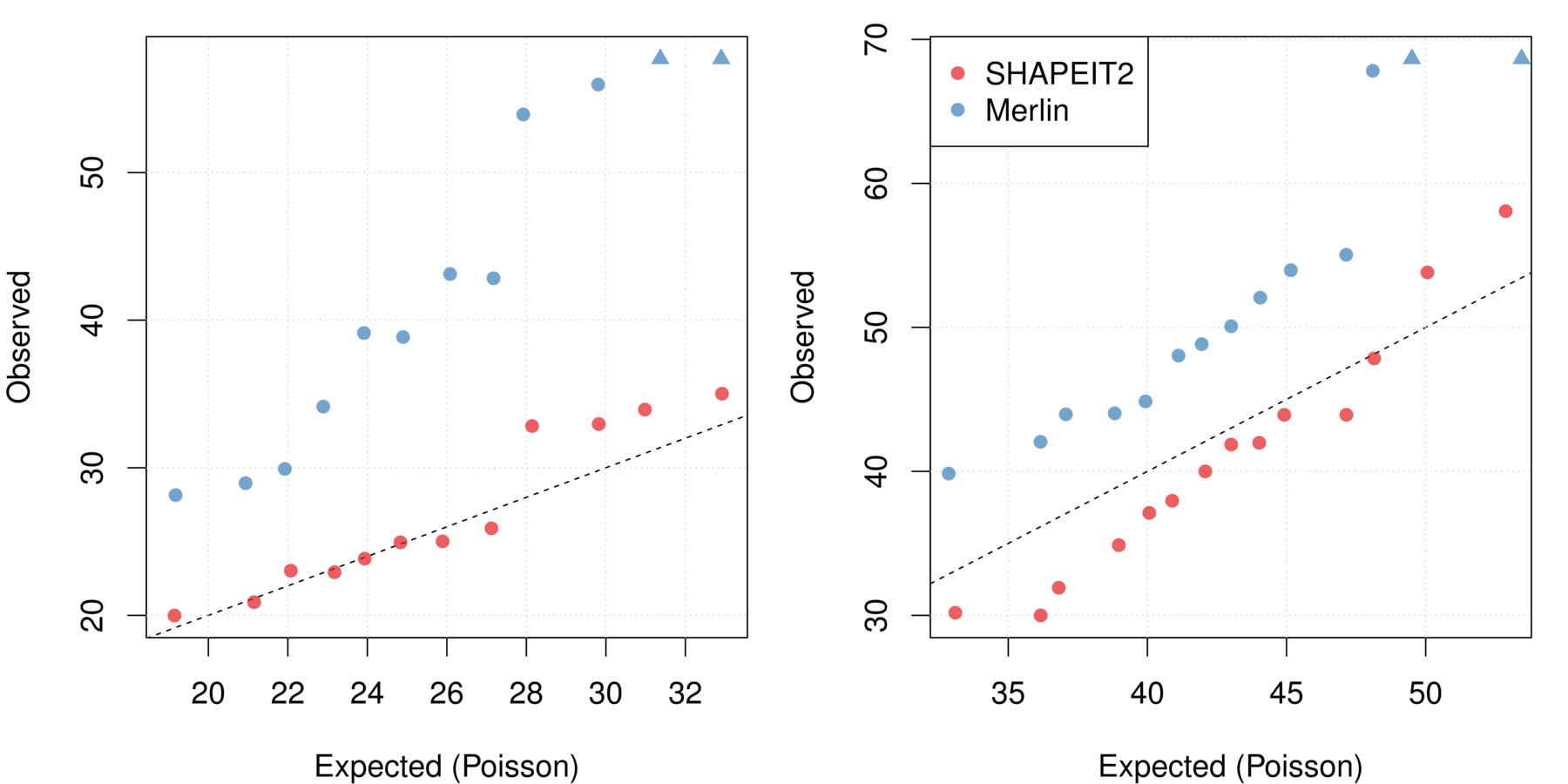

Supplement: Figure S31 — Vis cohort - recombination distributions. Q-Q plots for the observed against expected number of recombinations estimated by each method for paternal meioses (left) and maternal meioses (right), only duos that were part of an informative pedigree were used. For the expected distribution of recombination rates, a Poisson distribution using the genetic lengths from the 2002 deCODE Map was used (with rate parameter 42.81 and 25.9 for maternal and paternal recombinations respectively.) (TIFF) [file pgen.1004234.s031.tif]

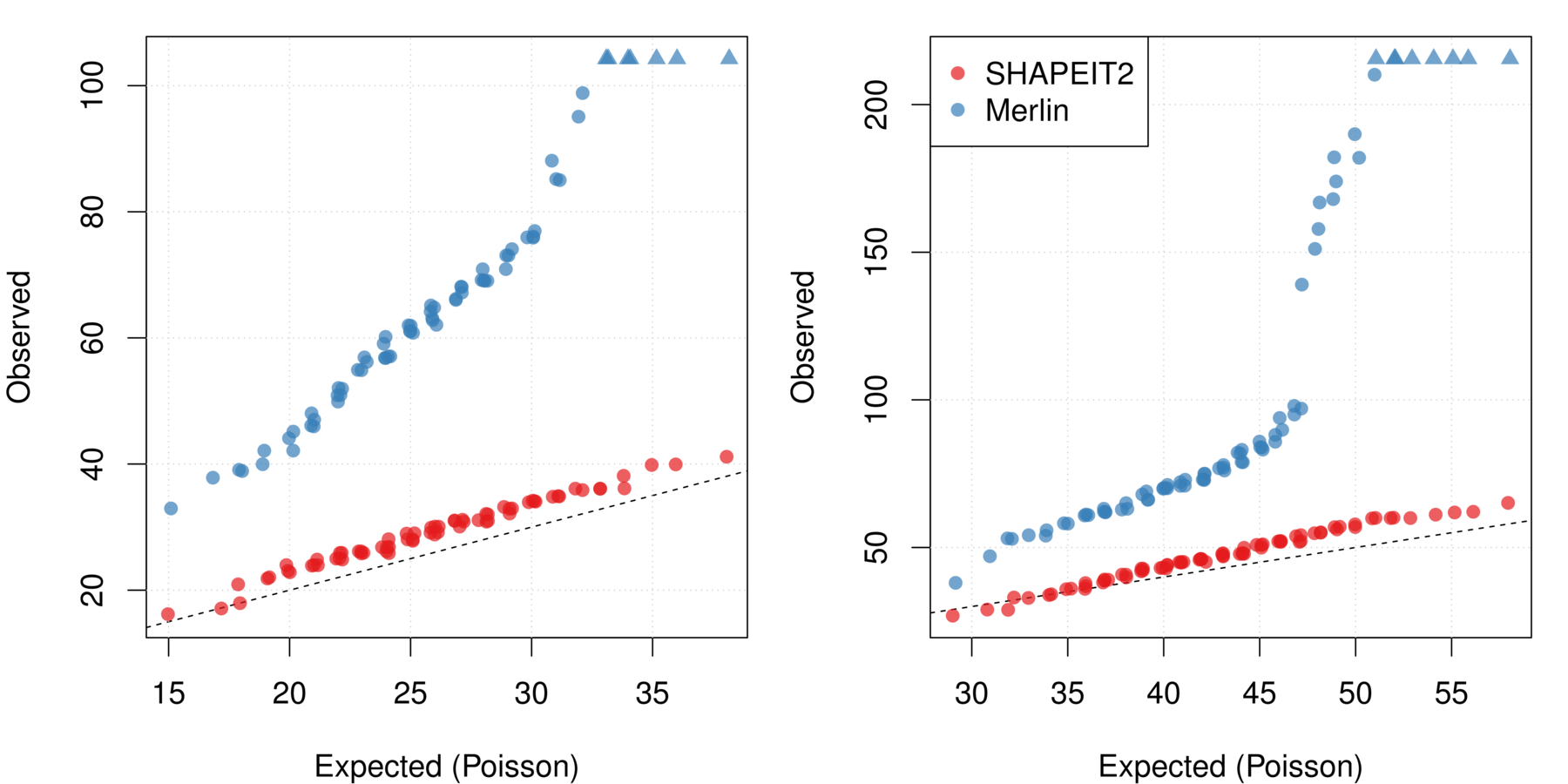

Supplement: Figure S32 — GPC cohort - recombination distributions. Q-Q plots for the observed against expected number of recombinations estimated by each method for paternal meioses (left) and maternal meioses (right), only duos that were part of an informative pedigree were used. For the expected distribution of recombination rates, a Poisson distribution using the genetic lengths from the 2002 deCODE Map was used (with rate parameter 42.81 and 25.9 for maternal and paternal recombinations respectively.) (TIFF) [file pgen.1004234.s032.tif]

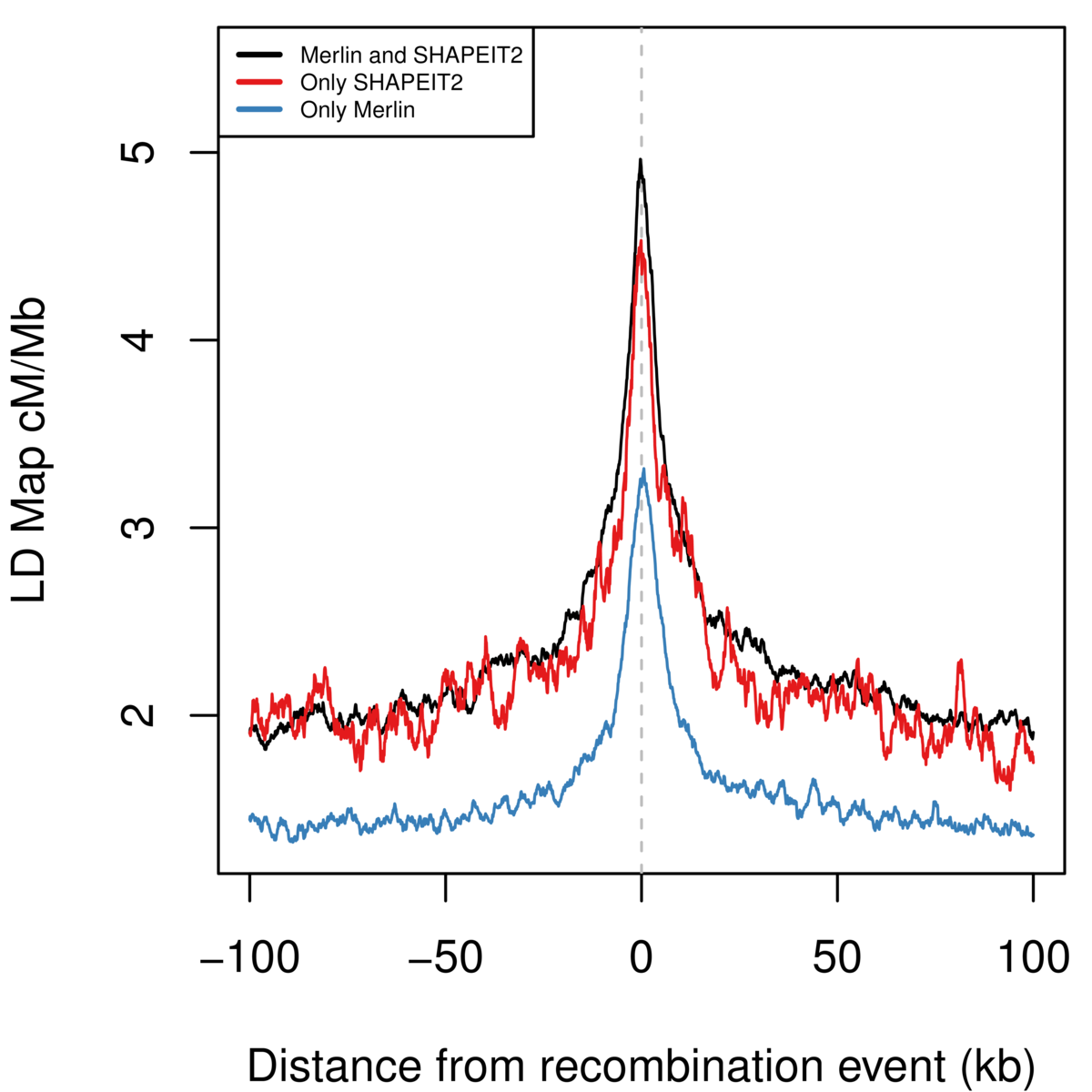

Supplement: Figure S33 — Recombination rates in regions around crossover detections by each method. Average recombination rate (from the HapMap LD map) centred on the location (the average of the two flanking heterozygous positions) of 34344 crossovers found by both SHAPEIT2 and Merlin (black), 4127 crossovers found only by SHAPEIT2 (red) and 32580 crossovers found only by Merlin (blue). Events found only by Merlin are in regions with less recombination on average than those found by by SHAPEIT2 suggesting a higher false detection rate. (TIFF) [file pgen.1004234.s033.tif]

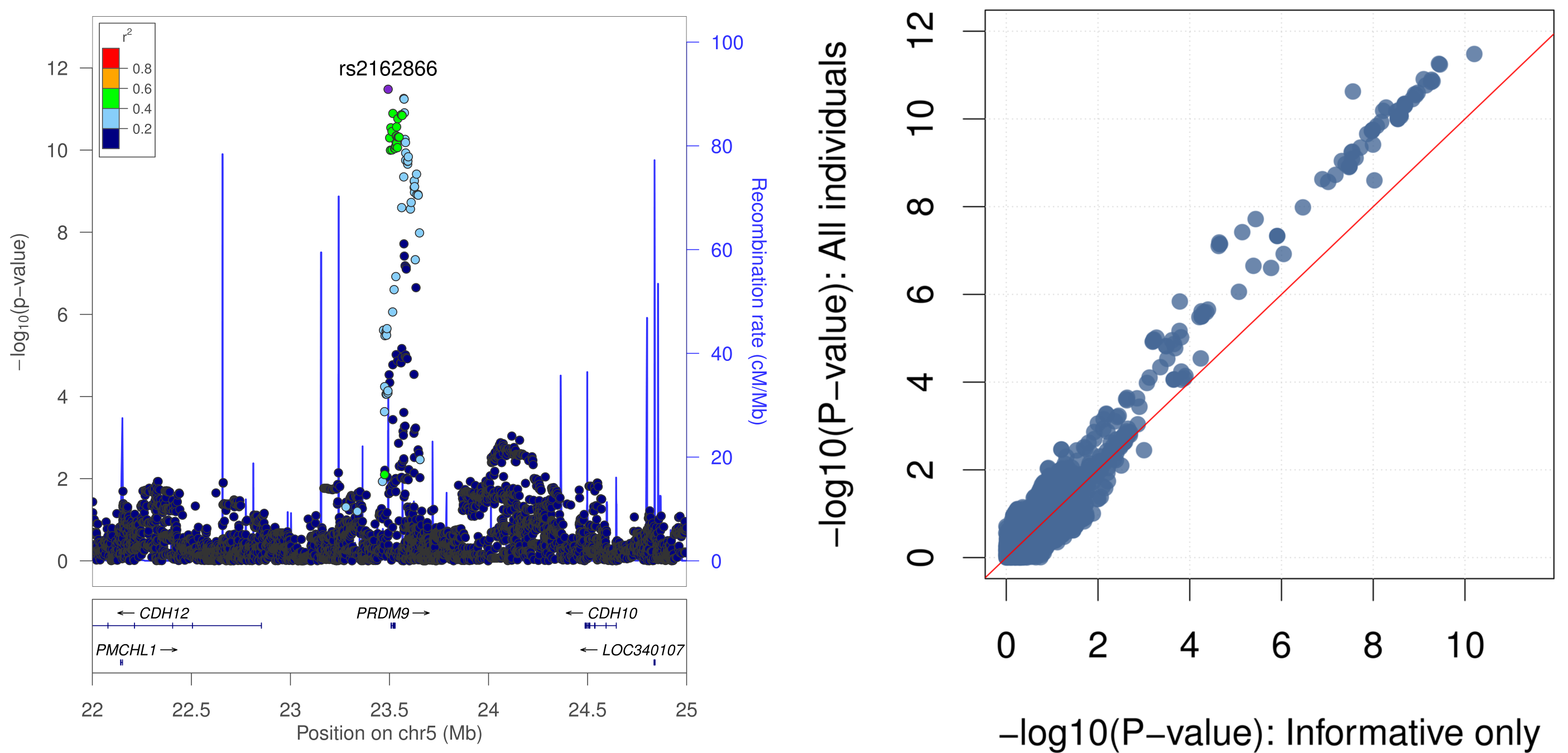

Supplement: Figure S34 — Association testing between PRDM9 region and hotspot usage phenotype for European cohorts. Left: The for the European meta-analysis for association between PRDM9 variants and the ‘hotspot usage’ phenotype. We used 618 informative and 466 uninformative parents in this analysis. Right: The of this analysis plotted against the when only the 618 informative parents are used. The additional samples yield a modest increase in power. (TIFF) [file pgen.1004234.s034.tif]
