# Supplementary material for: A General Approach for Haplotype Phasing across the Full Spectrum of Relatedness
Source: PLoS Genet. 2014 Apr 17;10(4):e1004234. doi: 10.1371/journal.pgen.1004234 (PMC3990520; doi:10.1371/journal.pgen.1004234)
Supplement: Table S1 — Frequencies of different pedigree sizes within each of the cohorts. Pedigrees of size 1 are individuals not part of an explicit pedigree. “Unrelated” is the sum of the number of pedigree founders and the number of individuals not in any pedigree. Note due to unspecified relationships, some of these individuals may still be closely related. (PDF) [file pgen.1004234.s035.pdf]

		Pedigree Size									
Cohort	Total	1	2	3	4	5	6	7	≥8	Founders	Unrelated
CARL	630	327	35	26	15	6	2	1	5	130	457
FVG	1236	612	65	61	20	9	7	4	11	274	886
GPC	2676	1712	156	107	48	19	5	2	0	419	2131
KOR	897	661	50	26	8	1	1	1	1	118	779
ORC	889	439	64	51	20	7	3	1	3	201	640
SPL	500	404	25	14	1	0	0	0	0	50	454
VB	1664	590	142	127	54	14	9	5	4	481	1071
VIS	960	653	67	28	11	4	3	1	0	150	803
