# Supplementary material for: A General Approach for Haplotype Phasing across the Full Spectrum of Relatedness
Source: PLoS Genet. 2014 Apr 17;10(4):e1004234. doi: 10.1371/journal.pgen.1004234 (PMC3990520; doi:10.1371/journal.pgen.1004234)
Supplement: Table S2 — The genotype confusion matrix used to simulate genotyping errors in our simulation studies. This is based on the discordance between Illumina Omni2.5S and Affymetrix Axiom chips on 1000 Genomes individuals. We took the Axiom genotypes as “truth” but halved the discordance and normalised the diagonal appropriately (the missing rate was left unchanged). This is to account for discordance that was actually due to Axiom chip errors. (PDF) [file pgen.1004234.s036.pdf]

		Observed genotype			
		AA	AB	BB	Missing
True genotype	AA	99.69100	0.04764	0.01286	0.24850
	AB	0.24437	99.35964	0.12842	0.26757
	BB	0.04536	0.11429	99.56244	0.27791
