# Supplementary material for: A General Approach for Haplotype Phasing across the Full Spectrum of Relatedness
Source: PLoS Genet. 2014 Apr 17;10(4):e1004234. doi: 10.1371/journal.pgen.1004234 (PMC3990520; doi:10.1371/journal.pgen.1004234)
Supplement: Table S3 — The average number of corrections applied to haplotypes for each method. ‘P’ and ‘M’ denotes when we correct a child's haplotypes using information from the parental (P) or maternal (M) haplotypes, to ensure consistent gene flow. ‘C’ denotes when multiple children were used to find the minimum recombinant parental haplotypes. Very few corrections are required for the SHAPEIT2 haplotypes compared to Beagle and HAPI-UR, this is also evident from the switch error improvements shown in Table 2. Cohort abbreviations: CARL - Carlantino, FVG - Friuli Venezia Giulia, GPC - Ugandan General Population Cohort, KOR - CROATIA-Korcula, ORC - Orkney Complex Disease Study, SPL - CROATIA-Split, VB - Val Borbera. VIS - CROATIA-Vis. (PDF) [file pgen.1004234.s037.pdf]

	SHAPEIT2			Beagle			HAPI-UR 3X		
	P	M	C	P	M	C	P	M	C
CARL	0.010	0.058	0.010	1.505	2.507	0.828	3.461	5.491	1.576
FVG	0.040	0.134	0.028	3.248	3.629	2.203	5.936	8.224	3.818
GPC	0.118	0.291	0.096	0.893	1.769	0.646	1.216	2.442	0.917
KOR	0.156	0.427	0.078	4.516	5.967	3.368	10.305	14.314	6.473
ORC	0.045	0.038	0.008	1.816	1.426	0.531	6.022	5.201	2.490
SPL	0.010	0.103	0.008	1.447	3.350	0.651	2.645	6.667	1.331
VB	0.076	0.143	0.019	2.900	4.367	1.176	6.127	9.112	2.373
VIS	0.058	0.200	0.006	4.378	7.326	1.092	7.264	12.404	1.990
